# Particle Size-Controlled Oxygen Reduction and Evolution Reaction Nanocatalysts Regulate Ru(bpy)_3_^2+^’s Dual-potential Electrochemiluminescence for Sandwich Immunoassay

**DOI:** 10.34133/research.0117

**Published:** 2023-04-14

**Authors:** Shijun Wang, Shu Zhu, Ziqi Kang, Xiangxiu Wang, Zixin Deng, Kun Hu, Jianjun Hu, Xiancheng Liu, Guixue Wang, Guangchao Zang, Yuchan Zhang

**Affiliations:** ^1^ Institute of Life Science and Laboratory of Tissue and Cell Biology, Lab Teaching & Management Center, Chongqing Medical University, Chongqing 400016, China.; ^2^ Key Laboratory for Biorheological Science and Technology of Ministry of Education, State and Local Joint Engineering Laboratory for Vascular Implants, Bioengineering College of Chongqing University, Chongqing 400030, China.; ^3^ Jinfeng Laboratory, Chongqing 401329, China.; ^4^ Department of Pathophysiology, Chongqing Medical University, Chongqing 400016, China.; ^5^ Department of Pathology, Guizhou Provincial People’s Hospital, Guiyang, Guizhou 550002, China.

## Abstract

Multiple signal strategies remarkably improve the accuracy and efficiency of electrochemiluminescence (ECL) immunoassays, but the lack of potential-resolved luminophore pairs and chemical cross talk hinders their development. In this study, we synthesized a series of gold nanoparticles (AuNPs)/reduced graphene oxide (Au/rGO) composites as adjustable oxygen reduction reaction and oxygen evolution reaction catalysts to promote and modulate tris(2,2′-bipyridine) ruthenium(II) (Ru(bpy)_3_^2+^)’s multisignal luminescence. With the increase in the diameter of AuNPs (3 to 30 nm), their ability to promote Ru(bpy)_3_^2+^’s anodic ECL was first impaired and then strengthened, and cathodic ECL was first enhanced and then weakened. Au/rGOs with medium-small and medium-large AuNP diameters remarkably increased Ru(bpy)_3_^2+^’s cathodic and anodic luminescence, respectively. Notably, the stimulation effects of Au/rGOs were superior to those of most existing Ru(bpy)_3_^2+^ co-reactants. Moreover, we proposed a novel ratiometric immunosensor construction strategy using Ru(bpy)_3_^2+^’s luminescence promoter rather than luminophores as tags of antibodies to achieve signal resolution. This method avoids signal cross talk between luminophores and their respective co-reactants, which achieved a good linear range of 10^−7^ to 10^−1^ ng/ml and a limit of detection of 0.33 fg/ml for detecting carcinoembryonic antigen. This study addresses the previous scarcity of the macromolecular co-reactants of Ru(bpy)_3_^2+^, broadening its application in biomaterial detection. Furthermore, the systematic clarification of the detailed mechanisms for converting the potential-resolved luminescence of Ru(bpy)_3_^2+^ could facilitate an in-depth understanding of the ECL process and should inspire new designs of Ru(bpy)_3_^2+^ luminescence enhancers or applications of Au/rGOs to other luminophores. This work removes some impediments to the development of multisignal ECL biodetection systems and provides vitality into their widespread applications.

## Introduction

Electrochemiluminescence immunoassay (ECLIA) is an advanced technology for detecting ultratrace amounts of active substances. It combines high-sensitivity chemiluminescence determination, highly controllable electrochemical analysis, and highly specific immune reactions [1,2]. However, most ECL analysis systems are based on a single signal mode (either “signal-on” or “signal-off” mode), which deteriorates the stability, accuracy, and efficiency of the signal output, as well as the integration and miniaturization of biosensors [3]. Multisignal output strategies achieve better detection efficiency and accuracy of the sensor when incorporated into the application of signal self-calibration or the simultaneous detection of multiple markers [4]. Multiple-signal ECL outputs rely on the introduction of resolvable signal output probes, with the potential-resolved strategy being the most widely used because of its reduced instrument requirements, accelerated test times, and enhanced sample throughput [5]. However, potential-resolved-based multisignal ECL systems are often plagued by the limited choices of luminophore pairs and mutual cross talk in or between co-reactants and luminophores [6,7]. Therefore, the development and simplification of a potentially resolvable ECL system is urgently needed for the further development of multisignal output platforms.

Previous studies showed that many single luminophores can produce dual ECL signals under different potentials in the presence of corresponding co-reactants, which can be utilized to build a dual-signal output ECL system [8]. Relevant research on single-luminophore-based potential-resolved systems can be divided into 2 categories, as reported in our previous study [9]. The first type relies on special chemical reactions between the metallic analytes and luminous systems. Cao et al. [10] used tris(2,2′-bipyridine) ruthenium(II) (Ru(bpy)_3_^2+^) as the only emitter with a cathodic co-reactant, Au-g-C_3_N_4_NSs on electrode, to achieve rapid ratiometric detection of Hg^2+^ by in situ generation of gold (Au) amalgam to produce anodic co-reactant g-C_3_N_4_NSs. The second type of system relies only on a single emitter and a single co-reactant, attaining the simplest ratiometric ECL system, where the ratio of the dual signal with a single-directional change at separate potentials was used as the output signal [11]. However, single-emitter systems with separate signal conversion designed for biomaterials (such as proteins, aptamers, and cells) are rarely reported owing to the high demand for self-resolved emission systems without the assistance of chemical reactions with analytes.

**Fig. 1.**
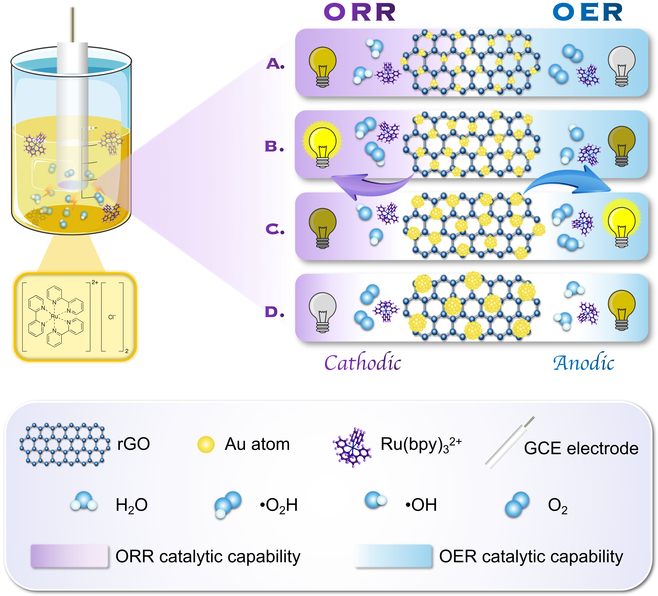
A schematic illustration of the conversion of anodic and cathodic luminescence of Ru(bpy)_3_^2+^ by regulating the particle size of AuNPs in Au/rGO with corresponding ORR/OER catalytic performance.

Hence, we innovatively introduced a series of similar co-reactants to realize the conversion of the anode and cathode signal outputs using Ru(bpy)_3_^2+^ as a single luminophore. Recent reports have revealed that the cathodic process of Ru(bpy)_3_^2+^ is related to the oxygen reduction reaction (ORR), and the generation of reactive oxygen species (ROS) can remarkably enhance luminescence [10]. Evidence has shown that the anodic process can be quenched by O_2_ while being enhanced by •OH, which is the final product and intermediate product of the oxygen evolution reaction (OER) process, the reverse reactions of the ORR [12]. Therefore, Au nanoparticles (AuNPs), acting as an ORR and OER catalyst, where their catalytic performance can be adjusted by their particle size [13–15], will act as an ideal reagent to regulate the anodic and cathodic luminescence of Ru(bpy)_3_^2+^ by producing different ROS (Fig. 1). However, using AuNPs alone can lead to blockage of the electron transfer by capping agents and restricted durability owing to their high surface energy [16–19]. Hence, in situ growth of Au on specific supports can be more desirable. As a robust 2-dimensional material combined with high surface-to-volume ratio, high conductivity, and good stability, reduced graphene oxide (rGO) is an ideal platform for growing or anchoring functional nanomaterials [20–22].

In this study, we proposed a new, facile, and low-cost approach to synthesize a series of Au/rGOs with different AuNP particle sizes and to measure their ORR and OER activities and their effects on the luminescence of Ru(bpy)_3_^2+^. We then systematically clarified the detailed mechanisms of the conversion of the anodic or cathodic luminescence of Ru(bpy)_3_^2+^ by controlling the diameter of AuNPs in Au/rGOs. Furthermore, we selected 2 of the synthesized Au/rGOs, the weak ORR and OER catalysts, with specific Au particle sizes as the ideal anodic and cathodic promoters of Ru(bpy)_3_^2+^, and introduced them into a simple and eco-friendly potential-resolved ratiometric immunoassay for carcinoembryonic antigen (CEA) detection.

## Results and Discussion

### Oxygen effect on the Au/rGO’s regulation of Ru(bpy)_3_^2+^ luminescence

#### ORR catalytic effect of Au/rGO on Ru(bpy)_3_^2+^ cathodic luminescence

**Fig. 2.**
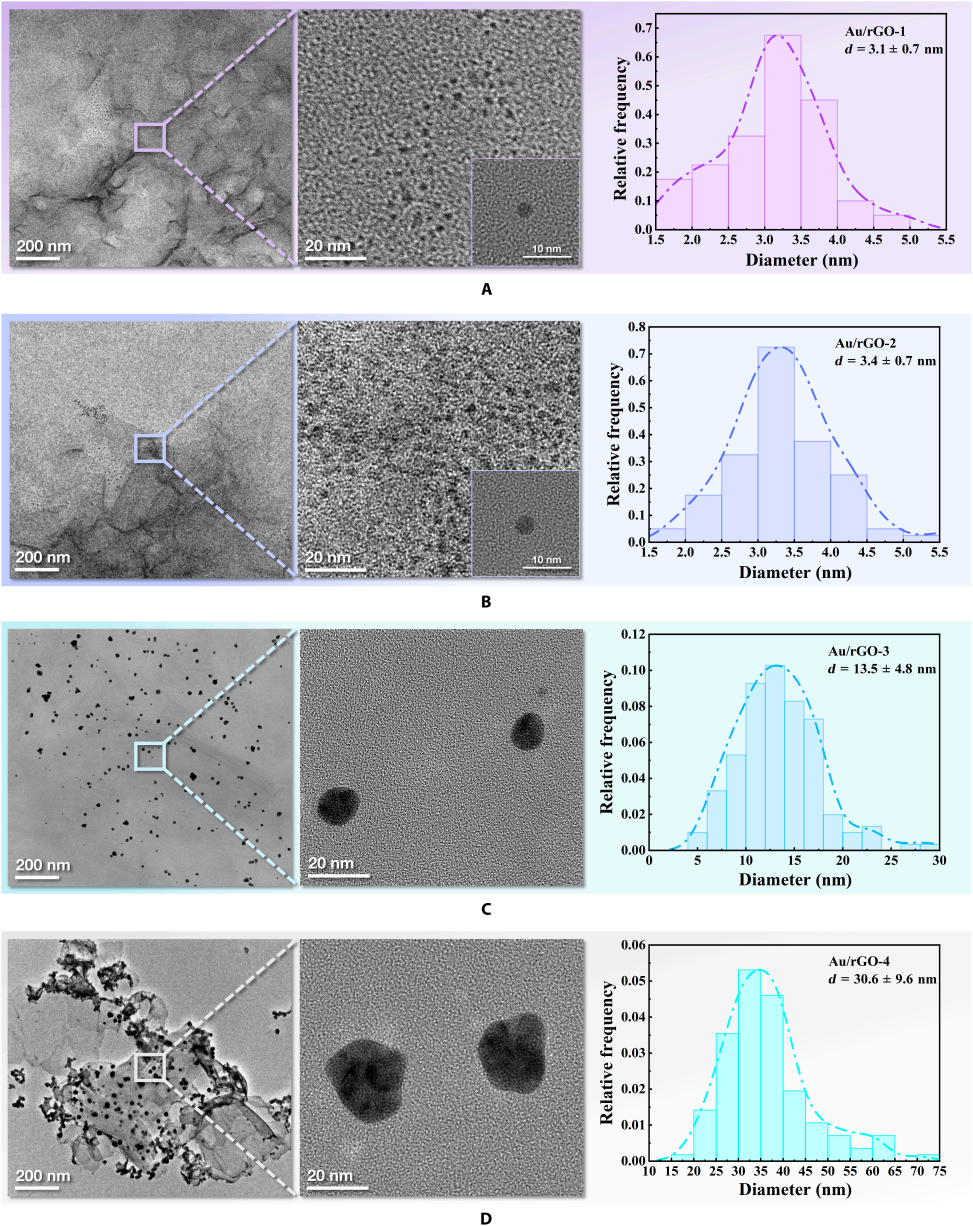
The characteristics of the particle size of Au/rGOs. Transmission electron microscopy image and the corresponding size histograms of (A) Au/rGO-1, (B) Au/rGO-2, (C) Au/rGO-3, and (D) Au/rGO-4.

**Fig. 3.**
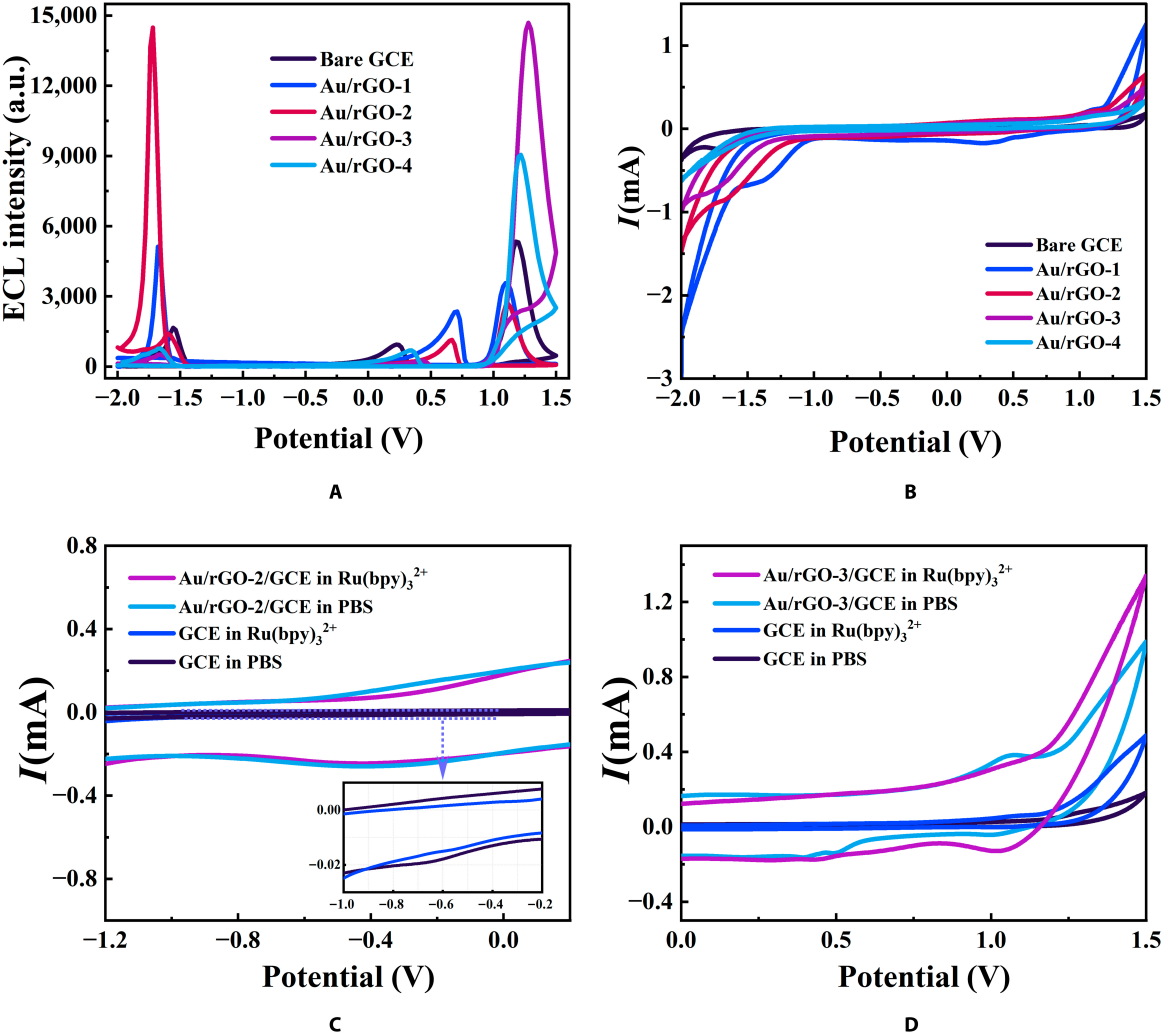
The CV and ECL performance of Au/rGOs. The comparison of (A) ECL and (B) CV performance of Au/rGO-1, Au/rGO-2, Au/rGO-3, and Au/rGO-4. a.u., arbitrary units. The comparison of the CV performance of Au/rGO-2 (C) and Au/rGO-3 (D) on GCE in Ru(bpy)_3_^2+^ (purple line) and PBS (light blue line) as well as bare GCE in Ru(bpy)_3_^2+^ (dark blue line) and PBS (black line) at a scan rate of 0.1 V/s.

The final product of the ORR is water, but higher Ru(bpy)_3_^2+^ cathodic ECL demands more ROS. Therefore, an appropriate luminescence accelerator should be an ORR catalyst that can generate adequate ROS and prevent a full reaction from generating excessive water. Fortunately, ORR catalyst Au/rGO has an adjustable catalytic performance that can be enhanced by downsizing AuNPs and vice versa [13]. Meanwhile, the d-band theory indicates that larger AuNPs possess higher d-band center positions because more of their surface atoms have low coordination numbers, making it easier for intermediates to break off the surface and increase ROS generation [23]. Hence, Au/rGO with medium AuNPs size should have the greatest effect on promoting Ru(bpy)_3_^2+^ cathodic ECL, as it not only has the capacity to rapidly catalyze O_2_ reduction but it also can result in the retention of more ROS products. Therefore, we synthesized a series of Au/rGO with the diameter of AuNPs gradually increased from 3 to 30 nm as the concentration of ydrogen tetrachloroaurate trihydrate (HAuCl_4_) increased from 30 to 300 mM (Fig. 2) [24]. As shown in Fig. 3A, a relatively strong cathodic luminescence of Ru(bpy)_3_^2+^ reacting with Au/rGO-1 (minimal diameter (3.1 nm) of AuNPs) was observed at −1.7 V. Au/rGO-2 with medium-sized AuNPs (3.4 nm) promoted a super strong cathodic ECL of Ru(bpy)_3_^2+^, which is higher than the corresponding peak of Au/rGO-1. The cathodic peaks of Ru(bpy)_3_^2+^ with the large- (13 nm) and maximal-sized (30 nm) AuNPs loaded on rGO (Au/rGO-3 and Au/rGO-4) were even lower than with a bare glassy carbon electrode (GCE). Therefore, the ECL result verified that Au/rGO exhibited robust cathodic co-reactant characteristics of Ru(bpy)_3_^2+^ at intermediate AuNP particle sizes.

Cyclic voltammograms (CVs) were recorded to investigate the electrode reactions occurring in the potential scan range. Except for the reversible wave of the oxidation–reduction reaction of Ru(bpy)_3_^2+^ (1.13 and 1.05 V) and the reduction–oxidation reaction of Ru(bpy)_3_^2+^ (−1.65 and −1.60 V) (Fig. 3B), the small peak at −0.65 V observed on GCE in phosphate-buffered saline (PBS) and Ru(bpy)_3_^2+^/PBS is attributed to the ORR (inset in Fig. 3C). The peak is greatly increased and redshifted to −0.4 V after the embellishment of Au/rGO-2 on GCE, which indicates its catalytic performance in the ORR process (Fig. 3C).

**Fig. 4.**
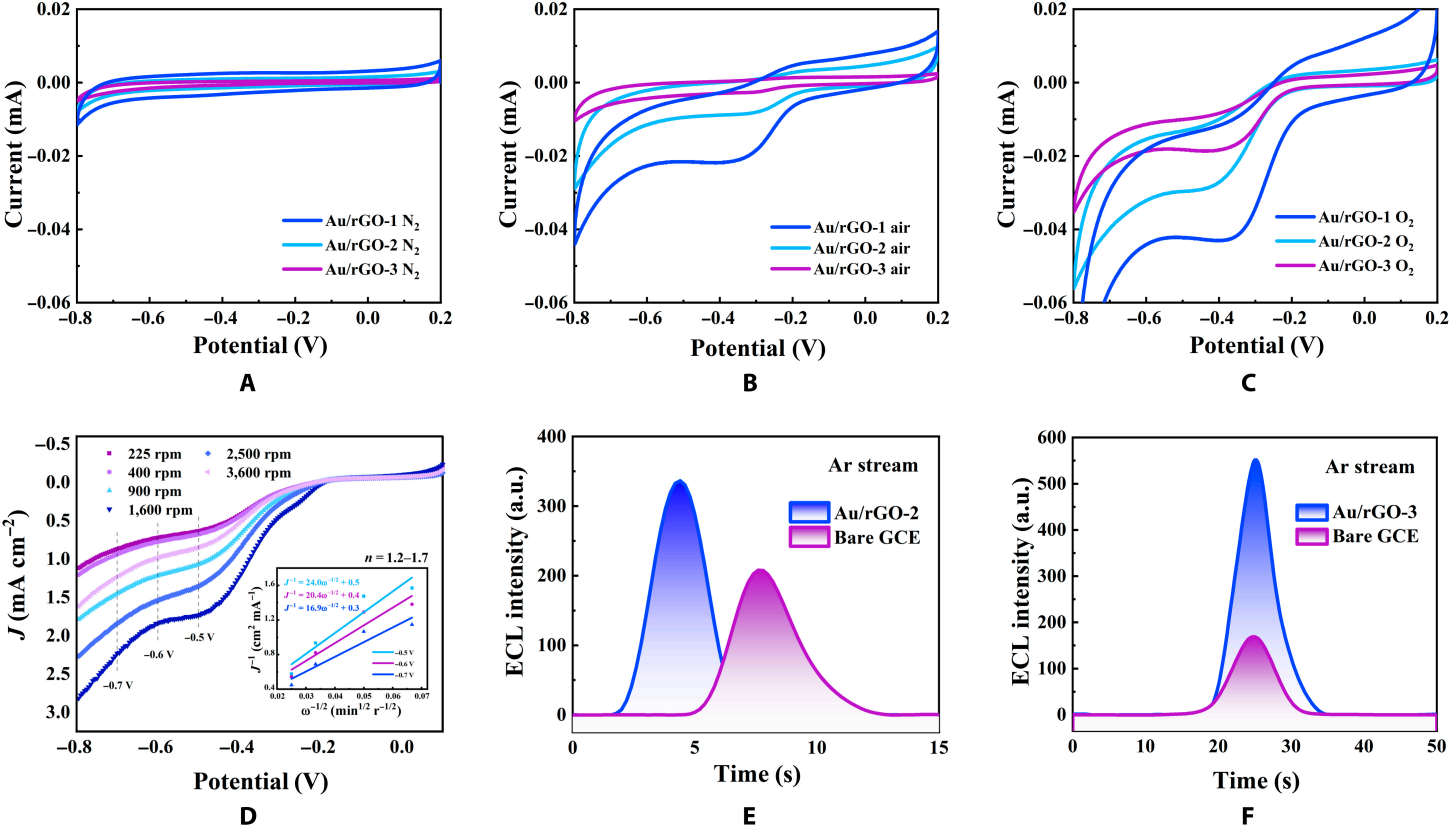
The ORR catalytic performance and the co-reactant capacity of the Au/rGOs. The comparison of the CV lines of Au/rGO-1 (dark blue line), Au/rGO-2 (light blue line), and Au/rGO-3 (purple line) on GCE in (A) N_2_-, (B) air-, and (C) O_2_-saturated 0.1 M KOH. (D) RDE lines of Au/rGO-2 in O_2_-saturated 0.1 M KOH at various rotation rates (ω) ranging from 225 to 3,600 rpm. Inset presents the K−L plots derived from the RDE measurements. All the scanning rates were 50 mV/s. ECL lines of bare GCE and Au/rGO-2 (−1.7 to −1.8 V versus Ag wire, scan rate of 0.1 V/s) (E) and Au/rGO-3 (F) in 1 mM Ru(bpy)_3_^2+^ acetonitrile solution (1.25 to 1.5 V versus Ag wire, scan rate of 0.1 V/s).

CV curve of oxygen reduction for series of Au/rGOs are recorded in O_2_-, air-, or N_2_-saturated 0.1 M KOH aqueous solutions to validate the existence of the ORR process and compare their catalytic capabilities. As shown in Fig. 4B, the ORR onset potential is around −0.20 V (versus Ag/AgCl) with a reduction peak around −0.35 V (versus Ag/AgCl), which is consistent with the findings of Yin et al. [25]. The inhibition and enhancement of the reduction peak in N_2_ and O_2_ atmospheres illustrate the participation of oxygen in the reaction, which confirms the ORR process (Fig. 4A to C). The adjustable catalytic performance of Au/rGO is exhibited under the same atmosphere by CV (Fig. 4A to C) and linear sweep voltammetry (LSV) (Fig. S8). Specifically, Au/rGO-1 with the smallest AuNP particle size exhibits the best ORR catalytic performance, while as the particle size increased, Au/rGO’s ORR catalytic ability deteriorated. The regulation of Au/rGO’s ORR catalytic activity by varying AuNP’s particle size may be attributed to the fact that smaller in situ growth of AuNPs on the rGO has less coordination, resulting in reduced electrophilicity of AuNPs, which decreases the activation energy of dissociative chemisorption of O_2_ on AuNPs, further facilitating the O_2_ reduction reaction and gaining [26]. It is proved by LSV that other noble metal NP (Ag and Pt)/rGO can also catalyze ORR (Fig. S9A). Among these, Au/rGO possesses the most potent properties for promoting Ru(bpy)_3_^2+^’s cathodic luminescence (Fig. S9B).

The oxygen reduction electron number was calculated on the basis of the LSV curves recorded on a rotating disk electrode (RDE) at different rotation rates (ω) to further clarify the major reaction process and products of the Au/rGO-2-catalyzed ORR reaction (Fig. 4D). The inset of Fig. 4D shows the Koutecky–Levich (K–L) plots (*J*^−1^ versus ω^−1/2^) at various electrode potentials. Calculated by the K–L equation [27], the transferred electron number (*n*) per oxygen molecule catalyzed by Au/rGO-2 is derived to be 1.2 to 1.7 across the potential range of −0.5 to −0.7 V. This result demonstrates that the 1-electron and 2-electron processes dominate O_2_’s reduction on Au/rGO-2, and the precise ROS produced by the ORR process is primarily O^2−•^ and H_2_O_2_, which play the dominant role in enhancing the cathodic ECL of Ru(bpy)_3_^2+^.

#### OER catalytic effect of Au/rGO on Ru(bpy)_3_^2+^ anodic luminescence

In the anodic ECL of Ru(bpy)_3_^2+^, O_2_-related reactions are also involved. As mentioned in many reports, •OH plays an important role in promoting the anodic luminescence of Ru(bpy)_3_^2+^ [28]. Additionally, oxygen has been reported to be toxic to anodic ECL because it can quench the excited-state Ru(bpy)_3_^2+^ to undergo the non-radiation pathway [12]. Therefore, the superiority of Au/rGOs as a controlled Ru(bpy)_3_^2+^ cathode or anode accelerator is better highlighted by the fact that they likewise exhibit OER catalytic activity and the same meso-size-adjustable OER catalytic capacity, where the OER catalytic performance of Au/rGO increases as the AuNPs particle size decreases [29]. Given that the result of the OER reaction is oxygen, a suitable anode promoter should not only catalyze the loss of one electron from H_2_O to create a high quantity of •OH but also avoid excessive O_2_ generation. Therefore, a weaker catalyst for the OER reaction is required to shift the reaction toward Eq. 1 rather than Eq. 2. Moreover, considering the d-band theory, Au/rGO with larger particle-size AuNPs is expected.


$$H_{2}O-e^{-}\to \text{HO}•+H^{+}$$ (1)

$$O_{2}{}^{-}-e^{-}\to O_{2}$$ (2)

As shown in Figs. 2C and D and 3A, the anodic ECL of Ru(bpy)_3_^2+^ at 1.1 V is substantially amplified when Au/rGO-3 and Au/rGO-4 with medium-large-sized (13 nm) and maximum (30 nm) AuNPs is loaded on the electrode. In addition, the anodic ECL of Ru(bpy)_3_^2+^ with Au/rGO-1 or Au/rGO-2 is weaker than that of the bare GCE, which can be due to the quenching effect of the OER-produced O_2_. The anodic ECL of Ru(bpy)_3_^2+^ with Au/rGO-3 is superior to that of Au/rGO-4 because the OER catalytic capability of Au/rGO-4 with too large AuNP particle size is extremely weak to create sufficient •OH, as shown in the CV diagram (Fig. 3D). Under anodic scanning of the bare GCE in Ru(bpy)_3_^2+^ solution, the CV current increased from the background current at 1.0 V owing to the oxidation of Ru(bpy)_3_^2+^, then grew more quickly because of the oxidation of water, and reached its peak at 1.5 V. The peak current value at 1.5 V was appropriately regarded as a symbol of OER degree because the oxidation of Ru(bpy)_3_^2+^ was supposed to result in a potential mass depletion of Ru(bpy)_3_^2+^ close to the electrode, and the shape of the summit current line at 1.5 V resembled a typical OER current [30]. In accordance with this notion, we employed CV to assess the OER catalytic performance of a series of synthesized Au/rGO catalysts. Figure 3B demonstrates that the OER catalytic capacity of Au/rGO-1 to Au/rGO-4 progressively decreased. This result confirm our inference that the anodic accelerator Au/rGO-3 with medium and weak OER catalytic properties can delay the OER reaction, prolonging the period during which the product •OH remains on the surface of the catalyst. Furthermore, the larger particle size of AuNPs allows •OH to have a greater propensity to desorb from Au/rGO-3, resulting in the accumulation of many •OH radicals in the vicinity of the electrode, which is conducive to strong ECL.

To confirm whether the ECL performance of Ru(bpy)_3_^2+^ modulated by altering the AuNP particle size of Au/rGOs was due to variable ORR and OER catalysis, we characterized the ECL enhancement effect of cathodic and anodic Au/rGOs under N_2_ and O_2_ atmospheres. As illustrated in Fig. S10A, the promotion effect of the cathode promoter Au/rGO-2 on (blue line) Ru(bpy)_3_^2+^ cathodic ECL was diminished in the N_2_ environment owing to the removal of dissolved oxygen in the system by N_2_, which inhibited the occurrence of ORR reactions. The enhancement of anodic luminescence was due to the disappearance of the quenching effect of dissolved oxygen on Ru(bpy)_3_^2+*^. By contrast, the ORR process was stimulated in an O_2_ environment (black line), resulting in increased Ru(bpy)_3_^2+^ cathodic ECL. Figure S10B shows the influence of the anode promoter Au/rGO-3 on the luminescence of Ru(bpy)_3_^2+^ in various atmospheres. Under N_2_ atmosphere (black line), the anode luminescence of Ru(bpy)_3_^2+^ was remarkably enhanced. This finding may be attributed to the fact that the Au/rGO-3-catalyzed OER reaction produces the intermediate product •OH and other ROS to promote anode luminescence, whereas the by-products O_2_ and dissolved O_2_ are removed by the N_2_ gas stream. The anodoluminescence of Ru(bpy)_3_^2+^ was remarkably diminished, and the cathodic ECL was barely enhanced after the introduction of O_2_. This finding is because Au/rGO-3 lacks a potent ORR catalytic capacity, and the increased dissolved O_2_ in the electrolyte has a strong quenching effect on the anodic luminescence of Ru(bpy)_3_^2+^. The preceding results demonstrate that the ORR catalytic capacity of Au/rGO-2 can affect in promoting Ru(bpy)_3_^2+^ cathodic ECL, whereas dissolved O_2_ in the solution and O_2_ created by the OER reaction on the electrode surface suppress Ru(bpy)_3_^2+^ anode luminescence. However, the exact components produced by the Au/rGO-2 catalytic ORR that promote Ru(bpy)_3_^2+^ cathodic ECL, as well as the independent role of •OH or other ROS components produced by Au/rGO-3 catalyzing OER that contribute to Ru(bpy)_3_^2+^ anodic luminescence, remain unknown. Therefore, we introduced superoxide dismutase (SOD) and isopropanol, inhibitors of •OH, as well as benzoquinone (BQ), inhibitor of O^2−•^, to independently reflect the effects of various catalyzed ROS components on Ru(bpy)_3_^2+^ cathodic and anodic luminescence. The results are shown in Fig. S11: SOD and isopropanol can reduce the Au/rGO-2-promoted cathodic ECL by approximately 55% and 90%, respectively, and BQ can reduce it by about 99%, indicating that Au/rGO-2 mainly fosters Ru(bpy)_3_^2+^’s cathodic ECL by catalyzing O_2_ reduction to O^2−•^, which is consistent with the results of the electron transfer number calculated by the K–L equation. For the anodic promoter Au/rGO-3 modified on the electrode, the anodic luminescence of Ru(bpy)_3_^2+^ decreased by approximately 20% when BQ was added, whereas SOD and isopropanol suppressed the anodic luminescence by about 50% and 70%, respectively, confirming that Au/rGO-3 was successfully manufactured as a weak OER catalyst, thereby implementing the concept of creating •OH as the primary OER product on the electrode surface to promote anodic luminescence.

### Co-reactant and other pathways for Au/rGO’s promotion of Ru(bpy)_3_^2+^ luminescence

Given the versatile properties of rGO, such as good catalytic ability, excellent electrical conductivity, and an abundance of functional groups [31], other mechanisms for rGO promoting Ru(bpy)_3_^2+^ luminescence should be investigated. For instance, rGO can participate in the co-reactant luminescence pathway of Ru(bpy)_3_^2+^ by leveraging its hydroxyl, epoxy, carbonyl, and carboxyl functional groups. Alternatively, rGO can directly promote luminescence by increasing the electron transfer efficiency of the reaction.

Acetonitrile was chosen as the appropriate ECL solvent to evaluate the co-reactant performance of Au/rGO, allowing all ORR and OER reactions involving O_2_, dissolved O_2_, and O atoms in H_2_O to be avoided [12]. The results are shown in Fig. 4E and F. In comparison to an empty GCE, the electrode modified with Au/rGO-2 has a weak promotion effect on Ru(bpy)_3_^2+^ cathodic ECL, whereas Au/rGO-3 can remarkably promote Ru(bpy)_3_^2+^ anode emission, which indicates that there could be other pathways for Au/rGO-3 to promote Ru(bpy)_3_^2+^ emission.

To further identify whether the mechanism of Au/rGO-3’s oxygen-free participation in Ru(bpy)_3_^2+^ anodic luminescence promoting is through direct catalysis or co-reactant pathway, we performed CV detection of Au/rGO-3 in PBS. The CV diagram in Fig. 3D exhibits a substantial reduction peak at 1.1 V, which may be attributed to the oxygen-containing groups (carboxyl and hydroxyl groups) on the surface of rGO directly gaining and losing electrons with the electrode, indicating that Au/rGO-3 may act as a co-reactant to undergo reduction–oxidation and oxidation–reduction reactions with Ru(bpy)_3_^2+^. Three tiny redox peaks emerging at 0.3, 0.4, and 0.5 V can be attributed to the electrode reduction of AuNPs. The anode peak of Ru(bpy)_3_^2+^ shifted to the left after the modification of Au/rGO-3 on the electrode, implying that Au/rGO-3 can perform as a catalyst to accelerate the electron transfer of Ru(bpy)_3_^2+^ [32].

In the process of •OH enhancing Ru(bpy)_3_^2+^’s anodic luminescence, Au/rGO-3 acts as an OER catalyst and provides the carboxyl group to promote Ru(bpy)_3_^2+^’s luminescence through the co-reactant pathway simultaneously. Carboxylic acid might be oxidized by the electrogenerated •OH to yield strong reducing intermediates CO_2_^−•^ that can function as a co-reactant and react with Ru(bpy)_3_^3+^ to emit light [28]. When Au/rGO-3 was electrochemically reduced at −1.0 V for 20 min on GCE, the anodic ECL fell remarkably, indicating that oxygenated groups are required for the anodic ECL (Figure S12). The reduction in the fraction of oxygen atoms after Au/rGO-3/GCE reacted with Ru(bpy)_3_^2+^ confirmed the aforesaid result (Fig. S13). Therefore, Au/rGO-3-catalyzed OER reaction to improve anodic luminescence is essentially achieved by accelerating the co-reaction reaction between Ru(bpy)_3_^2+^ and carboxyl groups under the anodic scan.

### Possible ECL mechanism of Au/rGOs regulating the conversion of the dual-potential ECL of Ru(bpy)_3_^2+^

To determine how AuNPs, GO, and rGO interact with Ru(bpy)_3_^2+^, we examined the co-reactant characteristics of AuNPs, GO, rGO, and their combinations in a Ru(bpy)_3_^2+^ solution. As shown in Fig. S14A, AuNPs with smaller particle sizes demonstrated a superior cathode promotion effect because of their preferable ORR electrocatalytic activity. This is because smaller AuNPs have more surface atoms that can absorb and activate O_2_ molecules than larger AuNPs [33]. Moreover, the degree of rGO reduction also has an impact on its co-reactant properties. As shown in Fig. S14B, GO possesses a certain anodic stimulation property that can be altered after the reduction of rGO by hydrazine hydrate, which has a greater cathodic ECL promotion capacity than GO. Considering that the elemental ratio characterized by x-ray photoelectron spectroscopy (XPS) shows a much lower ratio of NH_2_^−^ (2.68% of N1s) than oxygen-containing functional groups (28.16% of O1s; Table S2), the moderately poor anodic co-reactant property of rGO may be due to the anode promotion functions of amino groups on rGO being less significant, than the anodic luminescence degradation effect caused by the reduction of oxygen-containing functional groups. The effect of the degree of rGO reduction on the co-reactant performance of Au/rGO was further investigated by measuring the ratio of the cathodic and anodic luminescence (*I_c_*/*I_a_*) of Ru(bpy)_3_^2+^ co-reacted with various Au/rGO under different rGO reduction times. As shown in Fig. S15, the redshift of the ultraviolet-visible spectra indicates an increasing reduction degree of rGO, and *lg*(*I_c_*/*I_a_*) reaches its maximum at a reduction time of 6 h before decreasing with the extension of the reduction time. The optimum *I_c_*/*I_a_* of Ru(bpy)_3_^2+^ catalyzed by Au/rGO with partially reduced rGO can be attributed to the fact that the defective graphene substrate formed by the incomplete reduction reaction can accelerate charge transfer from AuNPs to O_2_, which lowers the energy barrier of the rate-limiting steps by destabilizing the intermediate species of ORR and decreasing the dissociation activation energy of O_2_, leading to increased ROS production [34]. However, the presence of either Au or rGO alone had a remarkably lower promoting effect on the Ru(bpy)_3_^2+^ cathode and anode luminescence than the 2 combinations, showing that the Au/rGO complex exerts its co-reactant feature through the interaction of Au and rGO as a whole (Fig. S14). This finding can be explained by few possible reasons, one of which is that the hybridization enhances the stability of AuNPs. Another potential explanation is that hybridizing AuNPs with rGO at graphene defects enhances their ORR catalytic activity by increasing charge transfer from the metal to the substrates.

On the basis of the discussed results, the principle of cathodic luminescence has been clarified clearly (Scheme S1). As Au/rGO-2 primarily catalyzes the 1e^−^ to 2e^−^ ORR, the major reactants in Ru(bpy)_3_^2+^’s cathodic luminescence are •O_2_H and H_2_O_2_, according to the electron transfer number (*n*) calculated using the K−L equation. Therefore, for the cathodic ECL reaction, Ru(bpy)_3_^2+^ is initially reduced at negative potentials to transform into Ru(bpy)_3_^+^. Then, the highly oxidative •O_2_H may directly oxidize Ru(bpy)_3_^+^ to Ru(bpy)_3_^2+*^ or oxidize Au/rGO to generate [Au/rGO]_ox_^•^, which indirectly oxidize Ru(bpy)_3_^+^ (pathway 1). Meanwhile, H_2_O_2_ can be electro-reduced to •OH, which also possesses strong oxidation properties. Similarly, •OH can oxidize Ru(bpy)_3_^2+^ to create a high-energy Ru(bpy)_3_^2+*^ state, thereby triggering cathode luminescence (pathway 2). Given that ROS has high oxidation potentials, •O_2_H and •OH may directly oxidize Ru(bpy)_3_^2+^ to create Ru(bpy)_3_^3+^, which can then react with Ru(bpy)_3_^+^ to release light via an annihilation reaction (pathway 3).

$$\mathrm{Ru}(\mathrm{bpy}{)}_{3}{}^{2+}+e^{-}\to \mathrm{Ru}(\mathrm{bpy}{)}_{3}{}^{+}$$ (3)

**Pathway 1:**

$$O_{2}+e^{-}+H^{+}\to •O_{2}H$$ (4)

$$\begin{array}{c} \begin{array}{c} \mathrm{Ru}(\mathrm{bpy}{)}_{3}{}^{+}+•O_{2}H+H^{+}\to \mathrm{Ru}(\mathrm{bpy}{)}_{3}{}^{2+*}+\\ H_{2}O_{2}\to \mathrm{Ru}(\mathrm{bpy}{)}_{3}{}^{2+}+hv \end{array} \end{array}$$ (5)

$$\mathrm{Au}/\mathrm{rGO}+•O_{2}H\to {{\left[\mathrm{Au}/\mathrm{rGO}\right]}_{\mathrm{ox}}}^{•}+H_{2}O_{2}$$ (6)

$$\begin{array}{c} \begin{array}{c} \mathrm{Ru}(\mathrm{bpy}{)}_{3}{}^{+}+{{\left[\mathrm{Au}/\mathrm{rGO}\right]}_{\mathrm{ox}}}^{•}+H^{+}\to \mathrm{Ru}(\mathrm{bpy}{)}_{3}{}^{2+*}+\\ \mathrm{Au}/\mathrm{rGO}\to \mathrm{Ru}(\mathrm{bpy}{)}_{3}{}^{2+}+hv \end{array} \end{array}$$ (7)

**Pathway 2:**

$$H_{2}O_{2}+H^{+}+e^{-}\to H_{2}O+•\mathrm{OH}$$ (8)

$$\begin{array}{c} \begin{array}{c} \mathrm{Ru}(\mathrm{bpy}{)}_{3}{}^{+}+•\mathrm{OH}+H^{+}\to \mathrm{Ru}(\mathrm{bpy}{)}_{3}{}^{2+*}+\\ H_{2}O\to \mathrm{Ru}(\mathrm{bpy}{)}_{3}{}^{2+}+hv \end{array} \end{array}$$ (9)

$$\mathrm{Au}/\mathrm{rGO}+•\mathrm{OH}\to {{\left[\mathrm{Au}/\mathrm{rGO}\right]}_{\mathrm{ox}}}^{•}+H_{2}O$$ (10)

$$\begin{array}{c} \begin{array}{c} \mathrm{Ru}(\mathrm{bpy}{)}_{3}{}^{+}+{{\left[\mathrm{Au}/\mathrm{rGO}\right]}_{\mathrm{ox}}}^{•}+H^{+}\to \mathrm{Ru}(\mathrm{bpy}{)}_{3}{}^{2+*}+\\ \mathrm{Au}/\mathrm{rGO}\to \mathrm{Ru}(\mathrm{bpy}{)}_{3}{}^{2+}+hv \end{array} \end{array}$$ (11)

**Pathway 3:**

$$\mathrm{Ru}(\mathrm{bpy}{)}_{3}{}^{2+}+•O_{2}H+H^{+}\to \mathrm{Ru}(\mathrm{bpy}{)}_{3}{}^{3+}+H_{2}O_{2}$$ (12)

$$\mathrm{Ru}(\mathrm{bpy}{)}_{3}{}^{2+}+•\mathrm{OH}+H^{+}\to \mathrm{Ru}(\mathrm{bpy}{)}_{3}{}^{3+}+H_{2}O$$ (13)

$$\begin{array}{c} \begin{array}{c} \mathrm{Ru}(\mathrm{bpy}{)}_{3}{}^{+}+\mathrm{Ru}(\mathrm{bpy}{)}_{3}{}^{3+}\to \mathrm{Ru}(\mathrm{bpy}{)}_{3}{}^{2+*}+\\ \mathrm{Ru}(\mathrm{bpy}{)}_{3}{}^{2+}\to \mathrm{Ru}(\mathrm{bpy}{)}_{3}{}^{2+}+hv \end{array} \end{array}$$ (14)

Because of the high oxidation potential of •O_2_H (*E*°(•O_2_H/H_2_O_2_) = 1.51 V versus SHE), •O_2_H is a sufficiently robust oxidizing agent to convert Ru(bpy)_3_^+^ into Ru(bpy)_3_^2+*^. To validate this feature, we used the following equation to calculate the energy (−Δ*H*°) accessible in Eq. 5.

$$-\Delta H{}^{\circ}\approx E{}^{\circ}\left(•O_{2}H/H_{2}O_{2}\right)-E{}^{\circ}(\mathrm{Ru}(\mathrm{bpy}{)}_{3}{}^{1+/2+})-0.1\;\mathrm{eV}$$ (15)

in which *E* (Ru(bpy)_3_^1+/2+^) = −1.41 V versus SHE, and 0.1 eV is an estimate of the entropy term (*T*∆*S*°) at 25 °C. −Δ*H*° is calculated to be 2.82 eV, and the energy required to produce Ru(bpy)_3_^2+*^ is 2.12 eV, indicating that the reaction has sufficient energy to produce Ru(bpy)_3_^2+*^, making Eq. 5 an energy-rich system. Given that the hydroxyl radical (•OH) has a higher oxidation potential (*E*°(H_2_O_2_/•OH) = 1.77–1.91 V versus SHE) than •O_2_H, the enthalpy of reaction Eq. 9 is at least 2.95 V (2.95 to 3.09 eV), making Eq. 9 theoretically possible.

For anode reactions, Au/rGO-3 can be employed as a co-reactant and an OER catalyst to enhance the luminescence of Ru(bpy)_3_^2+^. As the anodic co-reactant, Au/rGO-3 reacts with Ru(bpy)_3_^2+^ mainly through its surface oxygen-containing groups (carboxyl and hydroxyl) and amino groups to convert Ru(bpy)_3_^2+^ to the excited state, during which the amino groups perform similarly to tripropylamine [10,35], and the hydroxyl groups functions mechanistically comparable to alcohols and its derivatives [28,36–38] as the anodic co-reactant of Ru(bpy)_3_^2+^. In addition, as a weak catalyst for the OER process, Au/rGO-3 may catalyze the oxidation of water to •OH. Then, •OH enhances the decarboxylation of Au/rGO-COO^−^ to yield Au/rGO•, which can transfer electrons to Ru(bpy)_3_^3+^ to produce Ru(bpy)_3_^2+*^. Moreover, the interaction of a small amount of •OH with Au/rGO-COO^−^ might create CO_2_^−•^ with substantial reductivity, which can subsequently reduce Ru(bpy)_3_^2+^ to Ru(bpy)_3_^+^, followed by its interaction with Ru(bpy)_3_^3+^ in an annihilation-type luminescence process. Moreover, Au/rGO-3 could also accelerate the electron transfer as a catalyst to improve Ru(bpy)_3_^2+^’s luminescence efficiency. The specific equations are as follows:

$$\mathrm{Ru}(\mathrm{bpy}{)}_{3}{}^{2+}-e^{-}\to \mathrm{Ru}(\mathrm{bpy}{)}_{3}{}^{3+}$$ (16)

**Pathway 1:**

$$\begin{array}{c} \mathrm{Au}/\mathrm{rGO}(\text{ArCHOHR})-e^{-}\to [\mathrm{Au}/\mathrm{rGO}(\text{ArCHOHR}){]}^{+•}\to \\ {}[\mathrm{Au}/\mathrm{rGO}(\text{ArCOHR}){]}^{•} \end{array}$$ (17)

$$\begin{array}{c} \begin{array}{c} {\left[\mathrm{Au}/\mathrm{rGO}\left(\text{ArCOHR}\right)\right]}^{•}+\mathrm{Ru}(\mathrm{bpy}{)}_{3}{}^{3+}\to \mathrm{Ru}(\mathrm{bpy}{)}_{3}{}^{2+*}+\\ \mathrm{Au}/\mathrm{rGO}\left(\text{ArCOR}\right)\to \mathrm{Ru}(\mathrm{bpy}{)}_{3}{}^{2+}+hv \end{array} \end{array}$$ (18)

**Pathway 2:**

$$\mathrm{Au}/\mathrm{rGO}\left({\text{ArNRCH}}_{2}R^{\prime }\right)-e^{-}\to {\left[\mathrm{Au}/\mathrm{rGO}\left({\text{ArNRCH}}_{2}R^{\prime }\right)\right]}^{+•}$$ (19)

$${\left[\mathrm{Au}/\mathrm{rGO}\left({\text{ArNRCH}}_{2}R^{\prime }\right)\right]}^{+•}-H^{+}\to {\left[\mathrm{Au}/\mathrm{rGO}\left(\text{ArNRCH}R^{\prime }\right)\right]}^{•}$$ (20)

$$\begin{array}{c} \begin{array}{c} {\left[\mathrm{Au}/\mathrm{rGO}\left(\text{ArNRCH}R^{\prime }\right)\right]}^{•}+\mathrm{Ru}(\mathrm{bpy}{)}_{3}{}^{3+}\to \\ {\left[\mathrm{Au}/\mathrm{rGO}\left(\text{ArRN}=\mathrm{CH}R^{\prime }\right)\right]}^{+}+\mathrm{Ru}(\mathrm{bpy}{)}_{3}{}^{2+*}\to \\ \mathrm{Ru}(\mathrm{bpy}{)}_{3}{}^{2+}+hv \end{array} \end{array}$$ (21)

$$\begin{array}{c} {\left[\mathrm{Au}/\mathrm{rGO}\left(\text{ArRN}=\mathrm{CH}R^{\prime }\right)\right]}^{+}+H_{2}O-H^{+}\to \\ \mathrm{Au}/\mathrm{rGO}\left(\text{ArNRH}\right)+R^{\prime }\mathrm{CHO} \end{array}$$ (22)

**Pathway 3:**

$$H_{2}O-e^{-}\to H^{+}+•\mathrm{OH}$$ (23)

$$\begin{array}{c} \mathrm{Au}/\mathrm{rGO}-{\mathrm{COO}}^{-}+•\mathrm{OH}+H^{+}\to \mathrm{Au}/\mathrm{rGO}-{\mathrm{COO}}^{•}+\\ H_{2}O\to \mathrm{Au}/{\mathrm{rGO}}^{•}+{\mathrm{CO}}_{2}+H_{2}O \end{array}$$ (24)

$$\mathrm{Ru}(\mathrm{bpy}{)}_{3}{}^{2+}-e^{-}\to \mathrm{Ru}(\mathrm{bpy}{)}_{3}{}^{3+}$$ (25)

$$\begin{array}{c} \begin{array}{c} \mathrm{Au}/{\mathrm{rGO}}^{•}+\mathrm{Ru}(\mathrm{bpy}{)}_{3}{}^{3+}\to \mathrm{Au}/{\mathrm{rGO}}^{+}+\\ \mathrm{Ru}(\mathrm{bpy}{)}_{3}{}^{2+*}\to \mathrm{Ru}(\mathrm{bpy}{)}_{3}{}^{2+}+hv \end{array} \end{array}$$ (26)

Therefore, the transformation of Au/rGO from Ru(bpy)_3_^2+^’s anodic promoter to its cathodic promoter by increasing the particle size of AuNPs may be due to the following reasons. First, with the increase in the particle size of AuNPs, the capability of Au/rGO to catalyze the ORR and OER gradually decreases, resulting in an insufficient production of ROS, which weakens cathodic luminescence. Meanwhile, a relatively high ratio of •OH to O_2_ is produced in the OER process, which facilitates the anodic process. Second, with the increase in the size of the single AuNP particles covalently bonded on the surface of rGO, the distribution density of AuNPs decreased, thereby diminishing their masking effect on the reactive sites of the anodic co-reactants (amino groups, oxygen-containing groups, etc.) on the surface of rGO, leading to an enhancement of anodic luminescence.

### Construction and characterization of the single luminophore ratiometric ECL sandwich immunoassay

**Fig. 5.**
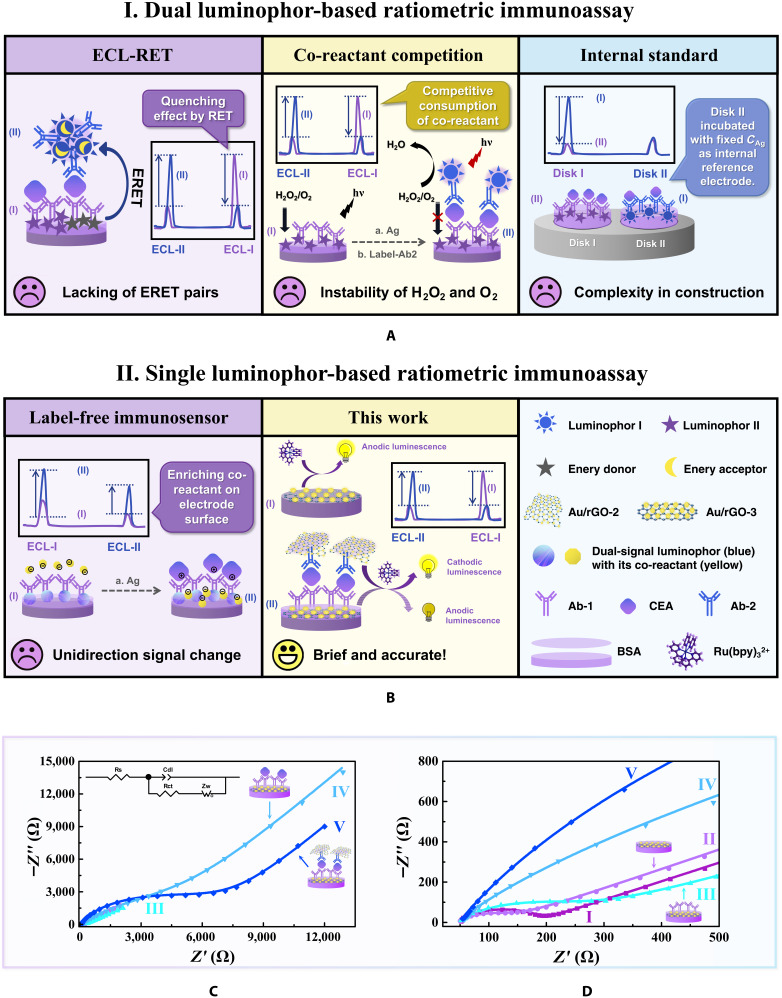
The schematic illustrations of various construction strategies of ratiometric immunosensor and the construction of the potential-resolved single-luminophore ratiometric ECL immunoassay. (A) Traditional building strategies for ratiometric immunosensor are mostly based on dual luminophores with their corresponding co-reactant, such as the ECL-RET strategy, competitive co-reactant-consuming strategy, and internal standard strategy. ERET, electrochemiluminescence RET. (B) Single luminophore with potential-resolved dual signal is introduced for building the ratiometric immunoassay to simplify the platform, which is usually applied in the label-free immunosensor. In this work, we innovatively implied the anodic and cathodic co-reactants of Ru(bpy)_3_^2+^ to be the substrate and label of the sandwich immunosensor for competitive consumption of Ru(bpy)_3_^2+^ dissolved in the electrolyte, which achieved the bidirectional signal change and accurate detection in a simple system. EIS (C) and the enlarged picture from 0 to 500 Ω (D) of (I) bare GCEs, (II) Au/rGO-3/GCE, (III) BSA/anti-CEA-1/Au/rGO-3/GCE, (IV) CEA/BSA/anti-CEA-1/Au/rGO-3/GCE, and (V) anti-CEA-2/CEA/BSA/anti-CEA-1/Au/rGO-3/GCE. EIS was measured in 0.1 mol l^−1^ of KCl containing 5.0 mmol l^−1^ of K_3_[Fe(CN)_6_]/K_4_[Fe(CN)_6_] (1:1). Rs, solution resistance; Cdl, double-layer capacitance; Rct, charge transfer resistance; Zw: Warburg impedance.

Ratiometric ECL assays have been widely applied in biosensing because they can provide more accurate results in complex environments by eliminating background signals and interferences through the self-calibration of the 2 emissions [39]. Although many reported ratiometric ECL systems based on ECL-resonance energy transfer (ECL-RET), competitive strategies, or enzymatic reactions have provided reliable and sensitive sensing signals, they mainly require 2 elaborately picked potential-resolved luminophores and complex combinations of co-reactants. Moreover, the multiple assembly steps and labeling courses could cause the chemical cross talk or energy transmission between ECL materials. Additionally, the existing single luminophore system has limited detection sensitivity for label-free construction models and unidirectional signal change output (Fig. 5A and B). Thus, if employing emission promoters rather than conventional luminophores as the substrate and label in an immunosensor and with the luminophore in the electrolyte, then the platform can be substantially simplified by utilizing the potential-resolved single-luminophore ECL system.

Therefore, we selected the most favorable cathodic and anodic promoters Au/rGO-2 and Au/rGO-3 to modify Ab_2_ and GCE for the construction of immunosensor for CEA detection, respectively. Electrochemical impedance spectroscopy (EIS) was conducted to track the stepwise assembly process of the electrode by using [Fe(CN)_6_]^3−/4−^ as the redox probe (Fig. 5C and D). Compared with the bare GCE (curve I), a smaller impedance value was observed for the Au/rGO-3-modified GCE (curve II) because of the good conductivity of rGO. The impedance values gradually increased after the electrode was assembled with Ab_1_ and bovine serum albumin (BSA) (curve III), CEA (curve IV), and Au/rGO-2-Ab_2_ bioconjugates (curve V) successively because the Ab_1_ and BSA, CEA, and Au/rGO-2-Ab_2_ bioconjugates acted as mass-transfer blocking layers that impeded electron transport. Importantly, these results indicated that the biosensor was successfully fabricated.

### Optimization of detection conditions

The selection of optimal test conditions is particularly important to achieve excellent performance in the ECL CEA assay [40]. In this system, we optimized 3 necessary factors: pH, concentration ratio of Au/rGO-2 and Au/rGO-3 as the label and substrate of the immunosensor, and concentration of Ru(bpy)_3_^2+^ (Fig. S16). The optimal pH value of the detection environment was controlled at 6 because the ECL ratio of the cathode to the anode (*I_c_*/*I_a_*) of the Au/rGO-2/GCE-Ru(bpy)_3_^2+^ system reached the maximum under cathode scanning, and Au/rGO-3 showed the lowest *I_c_*/*I_a_* at pH 6 owing to the prominent emission under anodic scanning. This indicates that both the cathodic and anodic luminescence promoters perform optimally at pH 6.

The appropriate label-to-substrate concentration ratio in a ratiometric immunoassay can ensure that the cathode and anode signals of the ECL could clearly invert with a change in the antigen concentration of the ratiometric sensor. The Ab_2_ tag Au/rGO-2 flips the anode-to-cathode signal with the increase in the antigen concentration by competing with the reaction of Ru(bpy)_3_^2+^ and Au/rGO-3 modified on the electrode. Therefore, if the concentration ratio of Au/rGO-2 to Au/rGO-3 is extremely low or high, then the ECL anode luminescence will be slow to change or the cathodic luminescence will always dominate, resulting in no significant change in the ratio of cathodic to anodic ECL intensity (*I_c_*/*I_a_*), which is disadvantageous to the detection sensitivity. Therefore, we optimized the Au/rGO-2-to-Au/rGO-3 ratio to 1:1 when the value of *I_c_*/*I_a_* reached its apex. Concurrently, *I_c_*/*I_a_* attained its maximum value at a Ru(bpy)_3_^2+^ concentration of approximately 1 mM. When an excess of Ru(bpy)_3_^2+^ was present in the solution, the addition of Au/rGO-2 had a minimal effect on the reaction of Au/rGO-3 with Ru(bpy)_3_^2+^, resulting in negligible inversion of the anode-to-cathode signal with the increase in the antigen concentration.

### Analytical performance of the ratiometric ECL biosensor

**Fig. 6.**
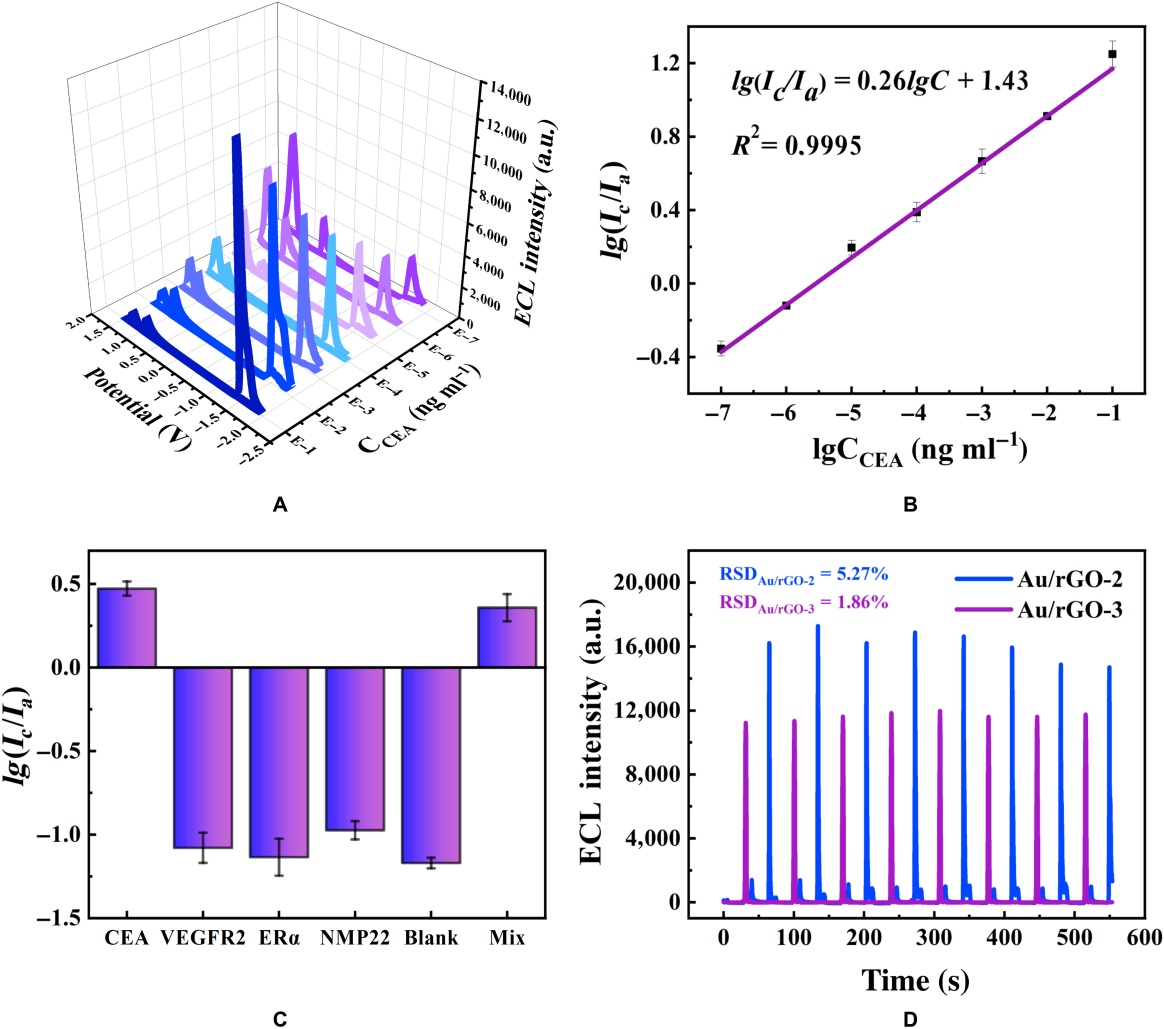
Detection performance of the ratiometric ECL immunosensor. (A) ECL responses of the ratiometric biosensor and (B) calibration plot of the ECL intensity with different concentrations of CEA from 10^−7^ to 10^−1^ ng/ml in 1 mM Ru(bpy)_3_^2+^. (C) The selectivity of the proposed ratiometric ECL biosensor. (D) Stability of ECL emission of Au/rGO-2 (blue line) and Au/rGO-3 (purple line) in Ru(bpy)_3_^2+^ for the detection of 10^−5^ ng/ml of CEA

With the increase in the CEA concentrations, the anodic intensity decreased, whereas the cathodic ECL intensity increased (Fig. 6A). Figure 6B demonstrates a strong linear association between the ECL intensity ratio of anodic-to-cathodic ECL intensity (*I_c_*/*I_a_*) and the logarithmic concentration of CEA in the range of 10^−7^ to 10^−1^ ng/ml. The regression equation was *lg*(*I_c_*/*I_a_*) = 0.26*lgC* + 1.43 with a correlation coefficient of *R*^2^ = 0.9995. Therefore, the suggested ratiometric biosensor for the detection of CEA had an outstanding analytical performance and had a low limit of detection of 0.33 fg/ml (signal-to-noise ratio = 3), which was superior to other strategies indicated in Table S1. The comparison of Au/rGO-2 with some traditional Ru(bpy)_3_^2+^ cathodic co-reactants shows its excellent cathodic ECL promotion performance (Fig. S17).

### Selectivity and stability

Different tumor markers (vascular endothelial growth factor receptor 2 (VEGFR2), estrogen receptor alpha (ERα), and nuclear matrix protein 22 (NMP22)) were used as interfering compounds at 10^−2^ ng/ml to evaluate the selectivity of the immunosensor. As shown in Fig. 6C, VEGFR2, ERα, and NMP22 have lower *lg*(*I_c_*/*I_a_*) compared with CEA. Because the interference groups only have Ab_1_ and no Ab_2_, its anode emission is extremely strong, and its cathode emission is modest. The detection value of *lg*(*I_c_*/*I_a_*) for a mixture of 10^−4^ ng/ml of CEA, 10^−2^ ng/ml of VEGFR2, 10^−2^ ng/ml of ERα, and 10^−2^ ng/ml of NMP22 is only slightly lower than 10^−4^ ng/ml of CEA, demonstrating the good selectivity of the proposed immunosensor. Figure 6D shows the stability of the ECL ratiometric approach at 10^−5^ ng/ml. The anodic and cathodic RSDs are 1.86% and 5.27%, respectively, indicating a robust biosensor stability.

### Detection of CEA in real human serum samples

To evaluate the analytical reliability and potential applicability of the constructed ratiometric immunosensor for testing real samples, we collected 10 human serum specimens with various CEA concentrations from the Guizhou Provincial People’s Hospital in accordance with the rules of the local ethical committee. Before measurement, serum samples were diluted appropriately in a stepwise manner to be within the linear range of the immune device using 0.01 M PBS and then monitored using the developed ECL immunoassay. The results were compared with those obtained using the commercialized ECLIA method (Electrochemiluminescent Analyzer, Roche E801, Switzerland). The results are listed in Table with the relative errors ranged from −7.54% to 9.25%. No significant difference was observed between the results obtained by 2 methods. Therefore, the ratiometric ECL biosensing system constructed in this work can be reasonably applied in the clinical determination of CEA in human plasma.

**Table.** Immunoassay of CEA in clinical serum samples using our method^a^ and the reference method^b^.

Sample	Proposed method (ng/ml)	Reference method (ng/ml)	Relative error (%)
1	269.13	283.0	−4.90
2	90.52	97.9	−7.54
3	31.90	29.2	9.25
4	15.30	14.9	2.68
5	1.35	1.24	8.87
6	0.82	0.8	2.50

^a^Provided as the mean of 3 repeated tests. In our approach, the sera were diluted 10,000-fold with PBS (pH = 7.4). RE, relative error.

^b^The reference method was the commercialized ECLIA that uses the Elecsys CEA, conducted on an electrochemiluminescent analyzer in the hospital.

## Conclusion

In this study, we proposed a simplified multisignal ECL system using only a single luminophore, Ru(bpy)_3_^2+^, and a similar co-reactant, Au/rGOs. The cathodic process of Ru(bpy)_3_^2+^ is related to the ROS produced in the ORR process, and the anodic process can be quenched by O_2_ and enhanced by •OH, the final product and the intermediate product of the OER. We synthesized a series of Au/rGOs with different AuNP diameters to investigate the relationship between their ORR and OER activities and their effects on the luminescence of Ru(bpy)_3_^2+^. The results demonstrate that Au/rGO with medium- to small-sized AuNPs mainly fosters cathodic ECL of Ru(bpy)_3_^2+^ by catalyzing the ORR to produce ample ROS while avoiding the complete reaction that generates excessive water. The weak OER catalyzer, Au/rGO with medium-large AuNPs, showed an evident promotion effect on the anodic luminescence of Ru(bpy)_3_^2+^ by generating •OH as the primary OER product and avoiding O_2_. This work solved the deficiency of the macromolecular co-reactant of Ru(bpy)_3_^2+^, which holds great promise for multisignal bioassay development. Moreover, the systematic clarification of the detailed mechanisms of converting the anodic or cathodic luminescence of Ru(bpy)_3_^2+^ by controlling the diameter of AuNPs in Au/rGOs should inspire further development of other luminescence enhancers of Ru(bpy)_3_^2+^ with excellent catalytic activity, such as single-atom catalysts, and can possibly be extended to other ECL luminophores using ROS as the co-reactants. Furthermore, the comparison of various ratiometric immunosensors and the new design of a single-luminophore co-reactant-based double anti-sandwich ratiometric immunosensors should provide new meaning for the dual-potential signal output luminophores, such as Ru(bpy)_3_^2+^, and pave the way for the development of more advanced multiple-signal strategies.

## Materials and Methods

### Materials

HAuCl_4_, silver nitrate (AgNO_3_), chloroplatinic acid (HPtCl_6_), palladium chloride (PdCl_2_), and sodium citrate were obtained from Aladdin Reagent Company. Ru(bpy)_3_^2+^ was acquired from Suna Tech Inc. Natural graphite powder (325 mesh) was purchased from Nanjing XFNANO Materials Tech Co. Ltd. Sodium borohydride (NaBH_4_), ethanethiol, ascorbic acid, and isopropanol were obtained from Sinopharm Chemical Reagent Co. Ltd. N-Hydroxy succinimide (NHS; Guaranteed Reagent (GR)) and 1-ethyl-3-(3-di-methylaminopropyl) carbodiimide hydrochloride (EDC; GR) were acquired from Shanghai Medpep Co. Ltd. (Shanghai, China). BSA (96% to 99%, GR) was purchased from Biss Inc. (Beijing, China). CEA and antibodies were supplied by Beyotime Biotechnology Co. Ltd. (Shanghai, China). Human serum sample were provided by ChongQing Medical University. PBS (0.1 M (pH 7.4)) containing KH_2_PO_4_ and Na_2_HPO_4_ in an appropriate ratio with KCl (0.1 M) was used for ECL detection. All other chemicals were of analytical grade and used as received without further purification. All the aqueous solutions were freshly prepared and diluted with ultrapure water (≥18 MΩ; Milli-Q, Millipore).

### Experimental design

#### Preparation of GO

GO was synthesized by the oxidation of natural graphite powder employing Hummers’ method, with a few modifications [41]. Briefly, graphite powder (3.0 g) was added to concentrated H_2_SO_4_ (70 ml) and was vigorously agitated in an ice bath. Then, 9.0 g of KMnO_4_ was gently added to maintain the suspension temperature below 20 °C. The reaction system was successively transferred to a 40 °C oil bath and aggressively agitated for approximately 30 min. After that, 150 ml of water was added, and the solution was agitated for 15 min at 95 °C. A further 500 ml of water was added, followed by a steady addition of 15 ml of H_2_O_2_ (30%), which changed the color of the solution from dark brown to yellow. To remove metal ions, the mixture was filtered and washed with 250 ml of 1:10 HCl aqueous solution. The resultant solid was dried in air and diluted to 600 ml to create an aqueous graphite oxide aqueous dispersion. Finally, it was dialysis-purified for 1 week using a dialysis membrane (Beijing Chemical Reagent Co., China) with a molecular weight cutoff of 8,000 to 14,000 g·mol^−1^ to eliminate the residual metal species. The resulting graphite oxide aqueous dispersion was then diluted to 1.2 l, agitated overnight, and sonicated for 30 min to exfoliate it to GO. The GO dispersion was then centrifuged at 3,000 rpm for 40 min to remove the unexfoliated graphite.

#### Preparation of rGO

To reduce GO to rGO, 2 mg of GO was dissolved in 2 ml of ultrapure water, and the resulting 2 mg/ml of GO and 2 ml of hydrazine hydrate were placed separately in 2 clean small beakers, with a large beaker covering both. After 6 h of stirring, the color of the GO fabric had changed from light brown to black, indicating the formation of rGO.

#### Preparation of Ab_2_ bioconjugates

Initially, the as-prepared Au/rGO-2 was sonicated for 45 min before use. After that, 120 μl of the 2 mg/ml of Au/rGO-2 was dispersed in newly prepared EDC (0.01 mol/l)/NHS (0.002 mol/l) solution. A total of 120 ml of 10 μg·ml^−1^ of Ab_2_ solution was added to the above solution and incubated at 4 °C for 10 h under constant temperature shaking conditions. After centrifugation (5000 rpm, 15 min), 120 ml of 1% BSA solution was added to block the nonspecific sites and oscillated at 4 °C for 2 h. The mixture was then centrifugated at 4000 rpm for 10 min. Finally, the obtained Au/rGO-2-Ab_2_ was redistributed into 120 ml of pH 7.4 PBS solution and stored at 4 °C until use.

#### Fabrication procedure for the ratiometric ECL biosensor

The fabrication process of the ratiometric ECL immunosensor was shown in Fig. 5B. Before modification, the bare GCE was cleaned by physical polishing and chemical/electrochemical washing and checked in the reversible electrode reaction of the Fe(CN)_6_^3−^/Fe(CN)_6_^4−^ pair. Then, 5 μl of 2 mg/ml of Au/rGO-3 was dropped onto the cleaned GCE and dried at room temperature. Subsequently, 5 μl of the EDC/NHS solution was used to activate the carboxyl group of Au/rGO-3 for 30 min at 37 °C. After washing with ultrapure water, 5 μl of 20 μg/ml of antibody (Ab_1_) was incubated on the modified electrode for 1 h at 37 °C. After removing the excess reagent, 5 μl of 1.0 wt% of BSA was dipped onto the modified electrode to block the nonspecific adsorption sites. Subsequently, 5 μl of CEA with different concentrations (10^−1^, 10^−2^, 10^−3^, 10^−4^, 10^−5^, 10^−6^, and 10^−7^ ng/ml) was added on the decorated electrode and incubated for 1 h at 37 °C. After washing with ultrapure water, a 5-μl Ab_2_ bio-conjugate was incubated on the modified electrode for 1 h at 37 °C to construct the ratiometric ECL immunosensor.

### Application of the ratiometric sensing platform for bioanalysis

CEA was detected on a double antibody sandwich-modified electrode in a solution of 100 mM pH 6.0 PBS containing 1 mM Ru(bpy)_3_^2+^ at room temperature using an ECL method with 600 V of bias and a potential scan spanning from +1.5 to −2 V.

### Electrochemical measurement

#### Cyclic voltammetry (CV)

CV was performed using a computer-controlled potentiostat (CHI660D) in a standard 3-electrode cell using Ag/AgCl (KCl saturated) electrode, a platinum wire as the counter electrode, and a GCE as the working electrode. Five microliters of Au/rGOs suspension was dropped onto a GCE with a diameter of 3 mm. The 3 electrodes were put into 0.1 M KOH solution. A flow of O_2_ or N_2_ was maintained for 40 min to achieve O_2_-saturated (maximum concentration of dissolved oxygen) or O_2_-free (substantial dissolved oxygen removal). The working electrode was cycled at least 5 times in the potential range from −2.0 to 1.5 V at a scanning rate of 100 mV/s.

#### Rotating disk electrode (RDE) measurements

The working electrode was prepared with the same method as that for CV. Before the measurement, a 30-min O_2_ flow was utilized to saturate the electrolyte in the cell with oxygen. The LSV experiments were conducted on RDE at varying speeds ranging from 225 to 3,600 rpm in O_2_-saturated 0.1 M KOH at room temperature. The K−L plots (scatterplot of *J*^−1^ versus ω^−1/2^) were drawn on the basis of the different rotational speeds (ω) and their corresponding current density (*J*) at the same voltage (Fig. 4D). Then, the scatterplots obtained at different voltages were linearly fitted; the slop of which is *k* (inset in Fig. 4D). The electron transfer number (*n*) can be calculated using the K–L equation:

$$\frac{1}{J}=\frac{1}{J_{L}}+\frac{1}{J_{K}}=\frac{1}{B\omega^{1/2}}+\frac{1}{J_{K}}$$

$$B=0.62\mathrm{nF}C_{O_{2}}D_{2}^{2/3}\nu^{-1/6}$$

where *J_K_* and *J_L_* are the kinetic and diffusion-limiting current densities, respectively; *n* is the number of electrons transferred in the reduction; and ω is the angular velocity. When the rotational speed (ω) unit is taking as revolutions per minute (r/min = rpm), $B=0.2\mathrm{nF}C_{O_{2}}D_{2}^{2/3}\nu^{-1/6}$. After substituting all constants (*F* is the Faraday constant (96,485 C mol^−1^), C_O_2__is the concentration of O_2_ in the solution (1.2 × 10^−6^ mol cm^−3^), *D*_O_2__ is the diffusion coefficient of O_2_ in solution (1.9 × 10^−5^ cm^2^ s^−1^), and *ν* is the kinematic viscosity of KOH (0.01 cm^2^ s^−1^)) [42], the result is *B* = 0.035*n*. Because the K–L equation can be transformed to *J*^−1^ = *k*ω^−1/2^ + *J_K_*^−1^(*k* = *B*^−1^), *k* is equivalent to the slope of the K−L plots’ fitted straight line, which can be derived from the plot. Then, substituting *B* = 0.035*n* into *k* = *B*^−1^ yields the electron transfer number *n*.

## Data Availability

Data supporting the findings of this study are available in the main text or the Supplementary Materials. Additional data related to this paper may be requested from the authors.

## Supplementary Materials

Supplementary 1 Fig. S1. (A) TEM image, (B) HAADF image, and (C) SEM image of Au/rGO. Fig. S2. DPV lines of rGO and Au/rGO on GCE in PBS. Fig. S3. EDS mappings of Au/rGO. Fig. S4. XRD patterns of rGO and Au/rGOs. Fig. S5. Zeta potential of GO, rGO, and Au/rGO. Fig. S6. XPS survey spectra of rGO (A), Au/rGO (B), C1s deconvolution spectrum N1s peak (C), and Au4f region of Au/rGO (D). Fig. S7. Raman spectrum of rGO and Au/rGOs. Fig. S8. RDE lines of Au/rGO-1, Au/rGO-2, and Au/rGO-3 in O_2_-saturated 0.1 M KOH. Fig. S9. (A) RDE lines in O_2_-saturated 0.1 M KOH at 1,600 rpm and (B) ECL line in 0.1 M Ru(bpy)_3_^2+^ of Au/rGO, Ag/rGO, and Pt/rGO. Fig. S10. ECL performance of Au/rGO-2 and Au/rGO-3 under O_2_, air, and N_2_ atmospheres. Fig. S11. ECL responses of Au/rGO-2 and Au/rGO-3 in Ru(bpy)_3_^2+^ with ROS inhibitor BQ, SOD, and isopropanol. Fig. S12. ECL curves of Au/rGO-3/GCE in Ru(bpy)_3_^2+^ before and after it was electrochemically reduced. Fig. S13. Oxygen and carbon atoms ratio of (A) Au/rGO-2 and (B) Au/rGO-3 before and after the reaction with Ru(bpy)_3_^2+^. Fig. S14. ECL performance of AuNPs with different diameters (A), GO and rGO (B), and Au/GO-1 and Au/GO-2 as well as Au/rGO-1 and Au/rGO-2 (C) in Ru(bpy)_3_^2+^. Fig. S15. Ultraviolet-visible absorption spectra of Au/rGO with different GO reduction degrees (A). The logarithm of the anodic to cathodic ECL luminescence intensity of Ru(bpy)_3_^2+^ reacting with Au/rGO with different rGO reduction times (B). Fig. S16. The effect of (A) pH, (B) *C*_[Au/rGO-2]_/*C*_[Au/rGO-3]_, and (C) Ru(bpy)_3_^2+^’s concentration on *lg*(*I_c_*/*I_a_*) signal output of the immunosensor. Fig. S17. The comparison of Au/rGO-2 with traditional Ru(bpy)_3_^2+^’s cathodic co-reactant GSH, K_2_S_2_O_8_, and H_2_O_2_. Scheme S1. The schematic illustration for cathodic and anodic ECL reaction pathways. Table S1. Comparison of the different potential-resolved platforms for ratiometric ECL immunoassay. Table S2. The XPS atomic of C1s, N1s, O1s, and Au4f on Au/rGO synthesized at different concentrations of HAuCl_4_
