# Supplementary material for: Particle Size-Controlled Oxygen Reduction and Evolution Reaction Nanocatalysts Regulate Ru(bpy)32+’s Dual-potential Electrochemiluminescence for Sandwich Immunoassay
Source: Research (Wash D C). 2023 Apr 14;6:0117. doi: 10.34133/research.0117 (PMC10243198; doi:10.34133/research.0117)
Supplement: Supplementary 1 — Fig. S1. (A) TEM image, (B) HAADF image, and (C) SEM image of Au/rGO. Fig. S2. DPV lines of rGO and Au/rGO on GCE in PBS. Fig. S3. EDS mappings of Au/rGO. Fig. S4. XRD patterns of rGO and Au/rGOs. Fig. S5. Zeta potential of GO, rGO, and Au/rGO. Fig. S6. XPS survey spectra of rGO (A), Au/rGO (B), C1s deconvolution spectrum N1s peak (C), and Au4f region of Au/rGO (D). Fig. S7. Raman spectrum of rGO and Au/rGOs. Fig. S8. RDE lines of Au/rGO-1, Au/rGO-2, and Au/rGO-3 in O2-saturated 0.1 M KOH. Fig. S9. (A) RDE lines in O2-saturated 0.1 M KOH at 1,600 rpm and (B) ECL line in 0.1 M Ru(bpy)32+ of Au/rGO, Ag/rGO, and Pt/rGO. Fig. S10. ECL performance of Au/rGO-2 and Au/rGO-3 under O2, air, and N2 atmospheres. Fig. S11. ECL responses of Au/rGO-2 and Au/rGO-3 in Ru(bpy)32+ with ROS inhibitor BQ, SOD, and isopropanol. Fig. S12. ECL curves of Au/rGO-3/GCE in Ru(bpy)32+ before and after it was electrochemically reduced. Fig. S13. Oxygen and carbon atoms ratio of (A) Au/rGO-2 and (B) Au/rGO-3 before and after the reaction with Ru(bpy)32+. Fig. S14. ECL performance of AuNPs with different diameters (A), GO and rGO (B), and Au/GO-1 and Au/GO-2 as well as Au/rGO-1 and Au/rGO-2 (C) in Ru(bpy)32+. Fig. S15. Ultraviolet-visible absorption spectra of Au/rGO with different GO reduction degrees (A). The logarithm of the anodic to cathodic ECL luminescence intensity of Ru(bpy)32+ reacting with Au/rGO with different rGO reduction times (B). Fig. S16. The effect of (A) pH, (B) C[Au/rGO-2]/C[Au/rGO-3], and (C) Ru(bpy)32+’s concentration on lg(Ic/Ia) signal output of the immunosensor. Fig. S17. The comparison of Au/rGO-2 with traditional Ru(bpy)32+’s cathodic co-reactant GSH, K2S2O8, and H2O2. Scheme S1. The schematic illustration for cathodic and anodic ECL reaction pathways. Table S1. Comparison of the different potential-resolved platforms for ratiometric ECL immunoassay. Table S2. The XPS atomic of C1s, N1s, O1s, and Au4f on Au/rGO synthesized at different concentrations of HAuCl4 [file research.0117.f1.zip › Certificate of English Editing.pdf]

Certificate of English Editing

To whom it may concern:

This memo certifies that one of our clients has contracted our academic editing service for the following file.

Order Number:
P-202302140851goh
Word Count:
5660 words
Date of the review:
02/14/23 (MM/DD/YY)

The English review was conducted using a two-stage process, in which a junior editor first reviewed the file, and then a senior editor conducted a final and more thorough review. All of our editors are native English-speaking professionals.

Documents receiving this certification should be English-ready for publication; however, the author has the ability to accept or reject our suggestions and changes.

We would like to emphasize that our service targets grammar and language edits. We do not rewrite the documents from scratch. If you are dissatisfied with specific revisions, please contact service@essaystar.com.

Essaystar Group

+1-208-975-4235
EssayStar, 93 S Jackson St, Seattle, WA 98104
