# Supplementary material for: Particle Size-Controlled Oxygen Reduction and Evolution Reaction Nanocatalysts Regulate Ru(bpy)32+’s Dual-potential Electrochemiluminescence for Sandwich Immunoassay
Source: Research (Wash D C). 2023 Apr 14;6:0117. doi: 10.34133/research.0117 (PMC10243198; doi:10.34133/research.0117)
Supplement: Supplementary 1 — Fig. S1. (A) TEM image, (B) HAADF image, and (C) SEM image of Au/rGO. Fig. S2. DPV lines of rGO and Au/rGO on GCE in PBS. Fig. S3. EDS mappings of Au/rGO. Fig. S4. XRD patterns of rGO and Au/rGOs. Fig. S5. Zeta potential of GO, rGO, and Au/rGO. Fig. S6. XPS survey spectra of rGO (A), Au/rGO (B), C1s deconvolution spectrum N1s peak (C), and Au4f region of Au/rGO (D). Fig. S7. Raman spectrum of rGO and Au/rGOs. Fig. S8. RDE lines of Au/rGO-1, Au/rGO-2, and Au/rGO-3 in O2-saturated 0.1 M KOH. Fig. S9. (A) RDE lines in O2-saturated 0.1 M KOH at 1,600 rpm and (B) ECL line in 0.1 M Ru(bpy)32+ of Au/rGO, Ag/rGO, and Pt/rGO. Fig. S10. ECL performance of Au/rGO-2 and Au/rGO-3 under O2, air, and N2 atmospheres. Fig. S11. ECL responses of Au/rGO-2 and Au/rGO-3 in Ru(bpy)32+ with ROS inhibitor BQ, SOD, and isopropanol. Fig. S12. ECL curves of Au/rGO-3/GCE in Ru(bpy)32+ before and after it was electrochemically reduced. Fig. S13. Oxygen and carbon atoms ratio of (A) Au/rGO-2 and (B) Au/rGO-3 before and after the reaction with Ru(bpy)32+. Fig. S14. ECL performance of AuNPs with different diameters (A), GO and rGO (B), and Au/GO-1 and Au/GO-2 as well as Au/rGO-1 and Au/rGO-2 (C) in Ru(bpy)32+. Fig. S15. Ultraviolet-visible absorption spectra of Au/rGO with different GO reduction degrees (A). The logarithm of the anodic to cathodic ECL luminescence intensity of Ru(bpy)32+ reacting with Au/rGO with different rGO reduction times (B). Fig. S16. The effect of (A) pH, (B) C[Au/rGO-2]/C[Au/rGO-3], and (C) Ru(bpy)32+’s concentration on lg(Ic/Ia) signal output of the immunosensor. Fig. S17. The comparison of Au/rGO-2 with traditional Ru(bpy)32+’s cathodic co-reactant GSH, K2S2O8, and H2O2. Scheme S1. The schematic illustration for cathodic and anodic ECL reaction pathways. Table S1. Comparison of the different potential-resolved platforms for ratiometric ECL immunoassay. Table S2. The XPS atomic of C1s, N1s, O1s, and Au4f on Au/rGO synthesized at different concentrations of HAuCl4 [file research.0117.f1.zip › Supporting Information.pdf]

Supporting Information

# Particle Size Controlled Oxygen Reduction and Evolution Reaction Nano-Catalysts Regulate $Ru(bpy)_3^{2+}$'s Dual-potential Electrochemiluminescence for Sandwich Immunoassay

Shijun Wang[1]†, Shu Zhu[1]†, Ziqi Kang[1]†, Xiangxiu Wang[2], Zixin Deng[1], Kun Hu[1], Jianjun Hu[5], Xiancheng Liu[2], Guixue Wang[2,3]*, Guangchao Zang[1,3,4]*, Yuchan Zhang[1,3]*

## Experimental Section

**Apparatus and Characterizations.** The ECL measurements were performed on a MPI-E multifunctional electrochemiluminescence analyzer (Xi'an Remex Analytical Instrument Ltd. Co., China). The three-electrode ECL cell was consisted of a modified glassy carbon as the working electrode (φ = 3 mm), an Ag/AgCl (KCl saturated) electrode as the reference, and a platinum wire as the counter electrode. The photomultiplier tube (PMT) was biased at 600 V, and the scan voltage was from 1.5 to -2 V with the scan rate of 100 mV/s. Ultraviolet−visible light (UV-vis) absorption spectra and fluorescence spectra were obtained with a spectrophotometer (Model UV2450, Shimadzu, Japan) and a spectrophotometer (Model F-7000, Hitachi, Japan), respectively. X-ray photoelectron spectroscopy (XPS) characterizations were measured by a VG Multilab 2000X instrument (Thermal Electron, USA). Fourier transform infrared spectra (FT-IR) were observed on a FT-IR spectrometer (ALPHA, Bruker). Transmission electron microscope (TEM) images were obtained using a JEM-2010 transmission electron microscopy (JEOL, Japan). Cyclic voltammograms (CVs) and electrochemical impedance spectroscopy (EIS) were carried out on an electrochemical workstation (Ivium, Netherlands). CVs were recorded in a potential range between −0.2 V and +0.6 V at a scan rate of 100 mV/s in a solution of 5 mM $K_3[Fe(CN)_6]/K_4[Fe(CN)_6]$ containing 0.1 M KCl. EIS measurements were performed by applying a voltage of 5 mV amplitude in 0.01 Hz to 106 Hz frequency range.

**Preparation of Au/rGOs, Ag/rGO and Pt/rGO.** Au/rGO-1,2,3 and 4 were prepared according to the literature with a slight modification.[1] $HAuCl_4$ (1.25uL; 2.5uL; 7.5uL of 2% $HAuCl_4$ was diluted to 10uL with ultrapure water for Au-rGO-1, 2 and 3, respectively; 10uL of 2% $HAuCl_4$ was used without dilution for Au-rGO-4,) was added to 2 mL rGO solution with agitation , sonicated for 10 minutes, and then stirred at room temperature for 2 hours, repeating the procedure three times. After 10 minutes of ultrasound, 25ul 0.01M freshly prepared $NaBH_4$ was swiftly added to the solution, swirled for 15 minutes, and then ultrasound for ten minutes; then 10uL 0.01M sodium citrate was dropped into the solution, stirred for 25 minutes, and then ultrasound for ten minutes. Centrifuge for 45 minutes at 15,000 rpm to remove excess $NaBH_4$ and sodium citrate, washed thoroughly with water, and finally redispersed in 2 mL of ultrapure water. Then centrifuge the resuspended solution at 12,000 rpm for 20 minutes to collect the unprecipitated component of the solution for further use and characterization. The synthesis process of Ag/rGO and Pt/rGO are identical to that of Au/rGO-2 by changing $HAuCl_4$ to $AgNO_3$ and $H_2PtCl_6 \cdot 6H_2O$, respectively.

**Characterization of Au/rGO.** The nanostructure of Au/rGO composite can be verified by the morphology analysis. As seen from Figure S1c, the typical wrinkled texture of Au/rGO film are exhibited in the scanning electron microscopy (SEM) image illustrating the presence of flexible and ultrathin rGO sheets. Besides, the spherical AuNPs particles which were clearly observed as the dark spots in TEM image and bright spots in high-angle annular dark-field (HAADF) image distributed in the background rGO sheets, which verifies the in-situ growth of AuNPs on the rGO sheet (Figure S1a, b).

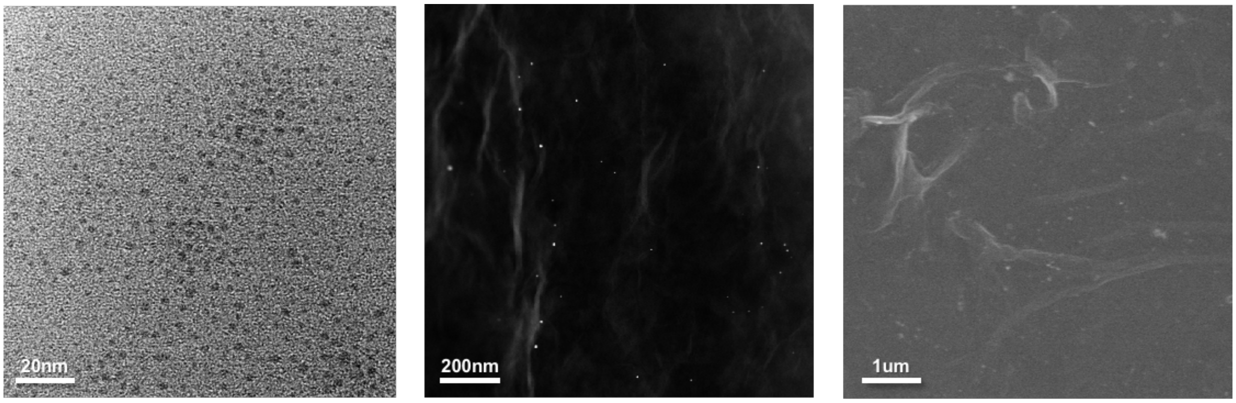


(a) (b) (c)

**Figure S1.** (a) TEM image, (b) HAADF image and (c) SEM image of Au/rGO

The particle size of AuNPs was verified by its oxidation peak potential (0.3V) according to Plieth equation:[2]

$$E_{AuNPs} = \left(-\frac{2\gamma V_m}{ZF}\right)\left(\frac{2}{d}\right) + E_{bulk}$$

Thereinto, $E_{bulk}$ is the oxidation potential of bulk metal (taken as 1.15V), $\gamma$ is the surface tension (1880 $erg \cdot cm^{-2}$), $V_m$ is the molar volume (10.21 $cm^3 \cdot mol^{-1}$), Z is the number of electrons (1), F is Faraday constant, and d is the NP diameter (3.4 nm). Therefore, the peak oxidation potential of AuNPs ($E_{AuNPs}$) (Au(0) to Au(I)) in Au/rGO-2 was calculated to be 0.92V (*vs*. SHE). $E_{AuNPs}$ versus Ag/AgCl electrode is converted according to the Nernst equation:[3]

$$E^{\circ}\text{vs Ag/AgCl} = E^{\circ}\text{vs SHE} - E^{\circ}_{Ag/AgCl} - (RT/(zF \cdot lge))pH$$

where $E^{\circ}$ of AuNPs is 0.92V *vs* SHE, and $E^{\circ}_{Ag/AgCl}$ is 0.22V *vs* SHE. The pH of the system is 6. So, $E^{\circ}$ AuNPs *vs* Ag/AgCl is calculated to be 0.35eV, which is in accordance with the CV peak of AuNPs (Au(0) to Au(I)) at 0.37V when 2 mol/L Au/rGO-2 is loading on the electrode, comparing to 2 mol/L rGO.

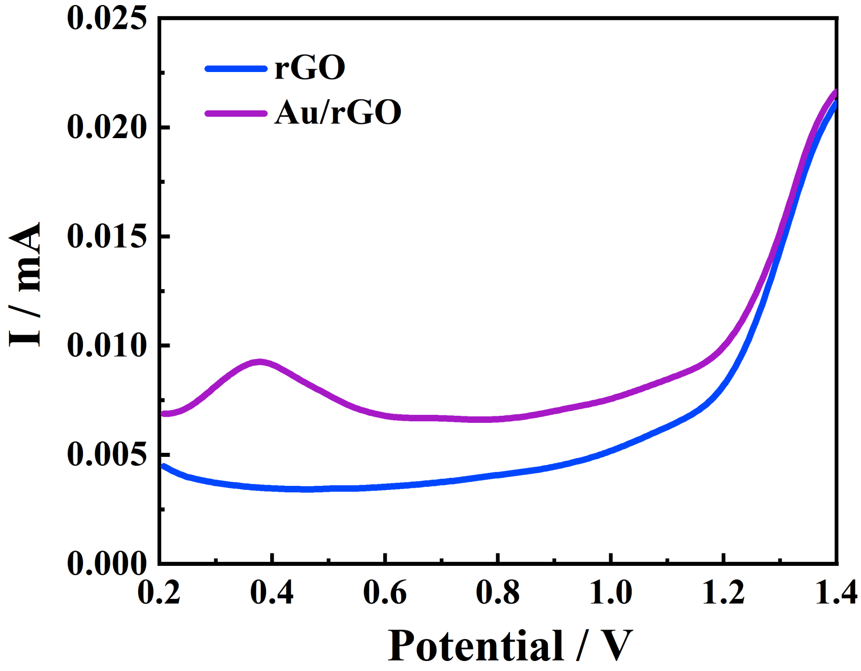


**Figure S2.** DPV lines of rGO (dark blue line) and Au/rGO (purple line) on GCE in PBS.

The analysis of chemical component was conducted by EDS mapping. The presence of AuNPs on rGO was further confirmed by EDS mapping Au/rGO composite which exhibited the characteristic peaks of C, N, O and Au, consistent with chemical components of graphene and AuNPs. The presence of oxygen characteristic peaks in EDS date demonstrated that the oxygen-containing groups on graphene oxide were not completely reduced.

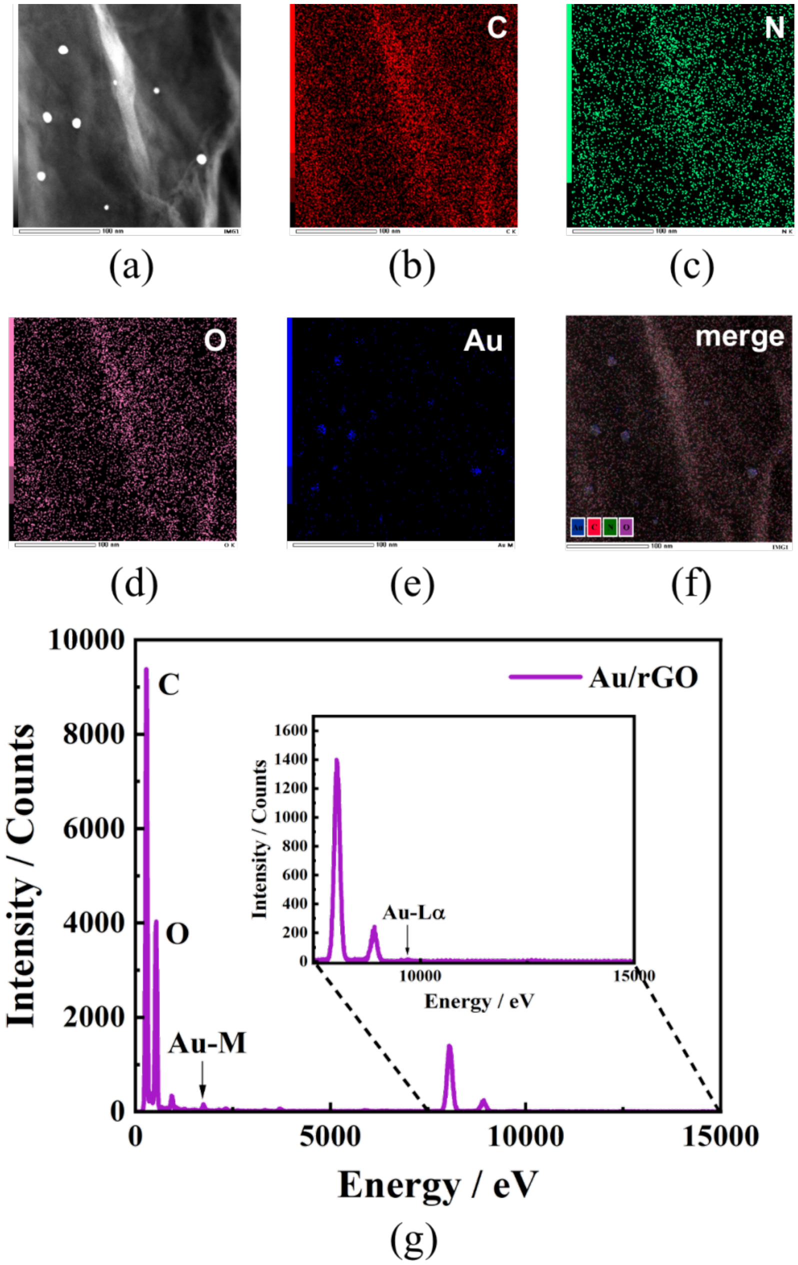


**Figure S3.** EDS mappings of Au/rGO.

X-ray diffraction (XRD) was used to confirm the composition of Au on rGO (Figure S4). The characterized diffraction peak at 29.0(0 0 2) of the dark blue line shows the existence of rGO, which also emerges in the diffraction spectrum Au/rGO in company with a distinctive characteristic diffraction peak at 38.3° and two peaks with inferior intensity at 44.5° and 82.4°corresponding

with Au (1 1 1), Au (2 0 0) and Au (2 2 2), respectively.[4] The results illustrate the composite of a face centered cubic (fcc) structure of AuNPs on the rGO.[5]

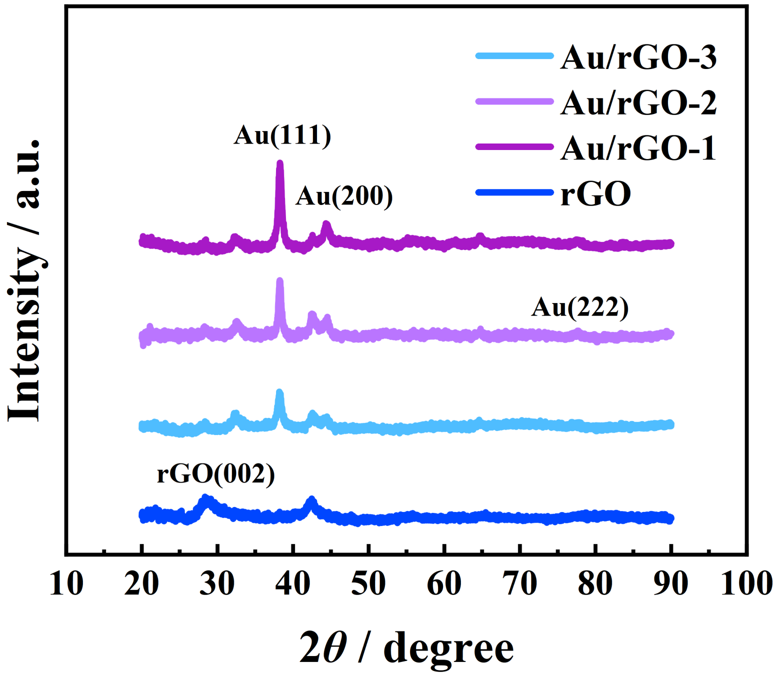


**Figure S4.** XRD patterns of rGO (dark blue line), Au/rGO-1 (dark purple line), Au/rGO-2 (light purple line), and Au/rGO-3 (light blue line).

The Zeta potential measurement of GO, rGOs and Au/rGO was conducted to inspect the surface charge characteristics. The average zeta potential of GO is approximately −44 mV originating from the negatively charged oxygen-containing functional groups such as carboxyl and epoxy groups at the surface of GO.[6] This value becomes more negative with −55 mV for rGO after the reduction process, which may be because the functionalization of rGOs during partial reduction resulted in higher surface negative charge densities compared to GO. This results in the steady dispersion of rGOs in an aqueous solution by virtue of electrostatic repulsion with water molecules.[7] An estimated value of -36 mV was found for the Zeta potential of Au/rGO. The

decrease in negative charge of Au/rGO compared to rGO demonstrated that AuNPs were successfully synthesized on rGO.[8]

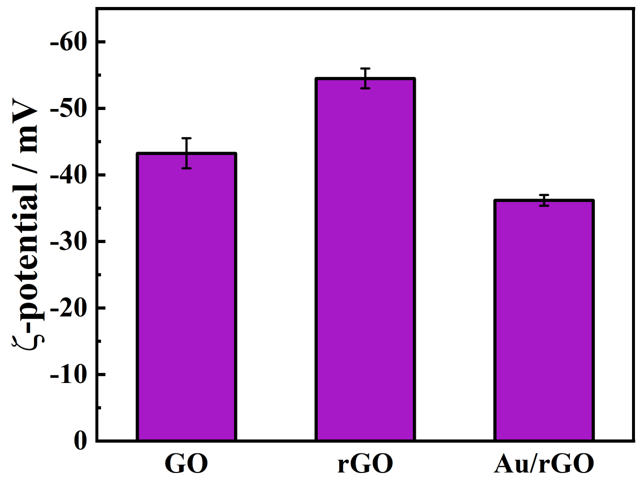


**Figure S5.** Zeta potential of GO, rGO and Au/rGO.

The modulated chemical structure of the rGO and Au/rGO were further confirmed by X-ray photoelectron spectra (XPS). It is evident from Figure S6a that carbon (C) and oxygen (O) are the primary constituents of rGO while Au/rGO is composed of C, O, and gold (Au). Nitrogen (N) take a very small proportion in rGO and Au/rGO.[9] Figure S6b illustrates the high-resolution C1s deconvolution spectrum of Au/rGO, which can be divided into four peaks centered at 284.6 eV (C-C/C=C in aromatic ring), 286.3 eV (C-O), 287.2 eV (C=O) and 288.4 eV (O-C=O),[10] the relatively high C=O peaks illustrate that rGO was not completely reduced. The high-resolution N 1s spectrum in Figure S6c can be deconvoluted into three Gaussian-Lorenzian peaks with the binding energies at 398.6 eV ($sp^2$-bonded nitrogen in N-containing aromatic rings (C−N=C)), 399.7 eV (tertiary nitrogen N−(C)3 groups) and 400.7 eV (amino group (C−N−H)),[11] which confirms the presence of amino groups on the Au/rGO. High-resolution Au 4f spectra obtained from an Au-rGO sample are shown in Figure S6d, with more dominant Au 4f7/2 and Au 4f5/2 spin-orbit doublets found at 88.3 eV and 84.5 eV in the binding energy scale attributing to Au(I), as well as the weaker Au

4f5/2 and Au 4f7/2 at 87.2 eV and 83.4 eV due to Au(0). Therefore, the chemical nature of gold within Au-rGO has been concluded to the combination of the metallic-Au(0)[12] and Au(I) presenting in Au–N bond.[13] The slight negative shift (0.4 eV) shown in region of the Au 4f spin-orbit doublet relative to the conventional reference positions of metallic-Au implies a robust interaction between Au and the rGO framework.[14]

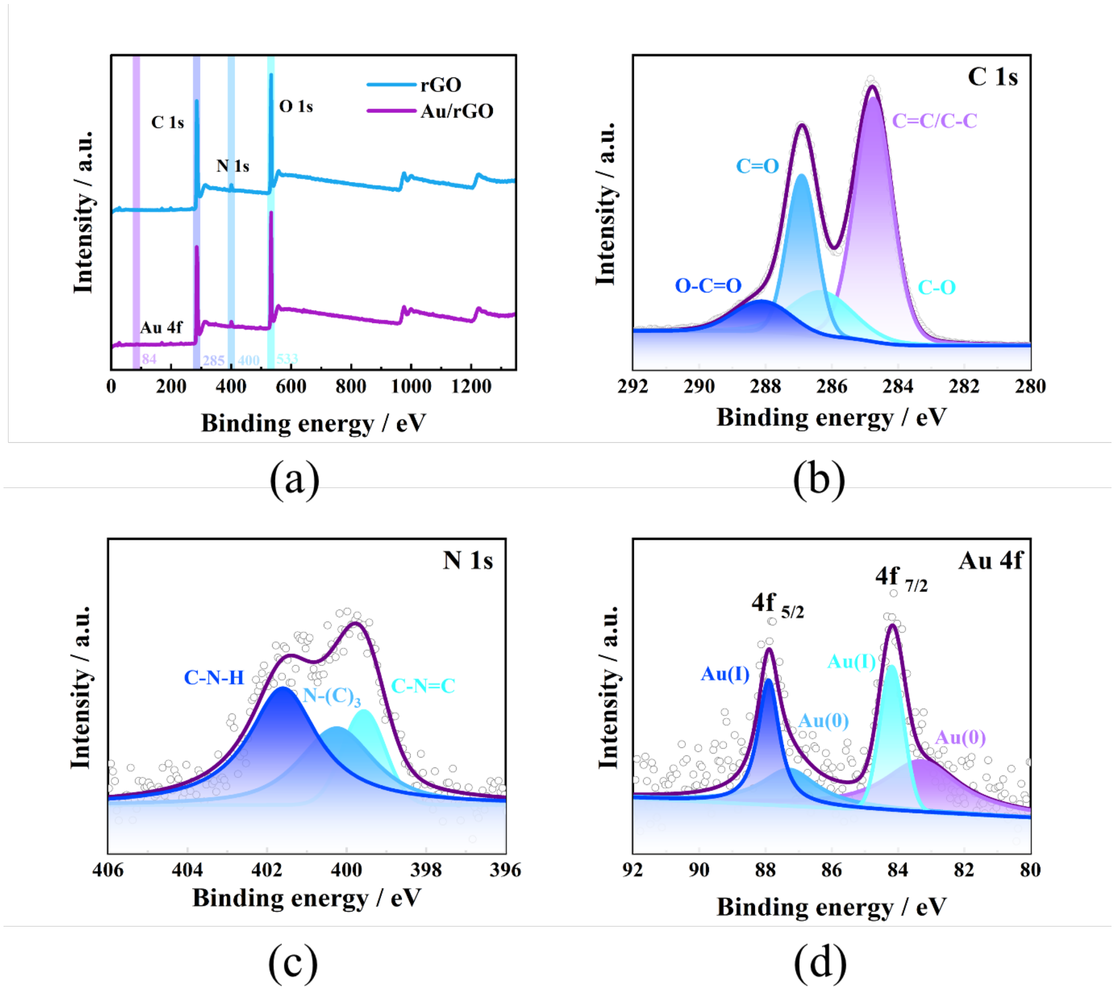


**Figure S6.** XPS survey spectra of (a) rGO and Au/rGO. (b) C1s deconvolution spectrum, (c) N 1s peak and (d) Au 4f region of Au/rGO.

Considering the nearly identical Raman spectra between Au/rGO and rGO, it is reasonable to assume that the formation of AuNPs has little effect on graphene's in-plane $sp^2$ domain sizes,

which is probably because the low load volume of AuNPs (0.01 atom %) (Table S2) does not appear to be sufficient to damage the rGO structurally.[15]

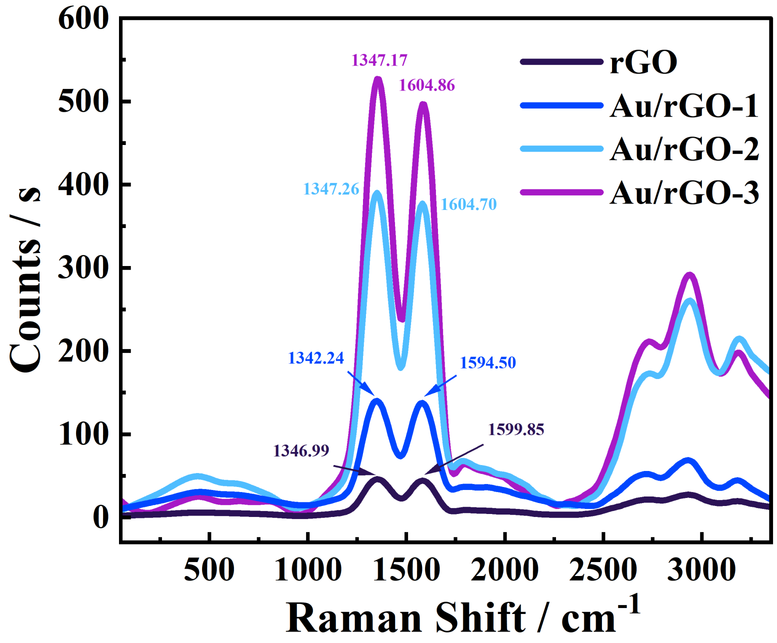


**Figure S7.** Raman spectrum of rGO, Au/rGO-1, Au/rGO-2 and Au/rGO-3.

According to the characterization results of TEM, EDS, XRD, XPS, and Zeta potential, AuNPs were successfully formed on the rGO surface by our one-step dual-reduction process. The negatively charged oxygen functional groups in rGO plays an important role in adsorbing positively charged Au ions via electrostatic interaction and act as reactive sites for the nucleation and growth of Au.[16]

The catalytic activities of Au/rGO with different AuNP particle sizes were characterized by linear sweep voltammetry (LSV) recording the corresponding rotating disk electrode (RDE) lines (1600 rpm) for the ORR. Figure S8 shows that the ORR onset potential of Au/rGO-2 (−0.20 V) is more positive than that of Au/rGO-3 (−0.25 V) but more negative than that of Au/rGO-1 (−0.18 V). The ORR current density (e.g., at −0.8 V vs Ag/AgCl) of Au/rGO-2 is also significantly greater than

that of Au/rGO-3, whereas it is lower than that of Au/rGO-1. The LSV result indicate that Au/rGO with smallest AuNPs particle size exhibits the best ORR catalytic performance, while as the AuNPs particle size increased, Au/rGO's ORR catalytic ability deteriorated.

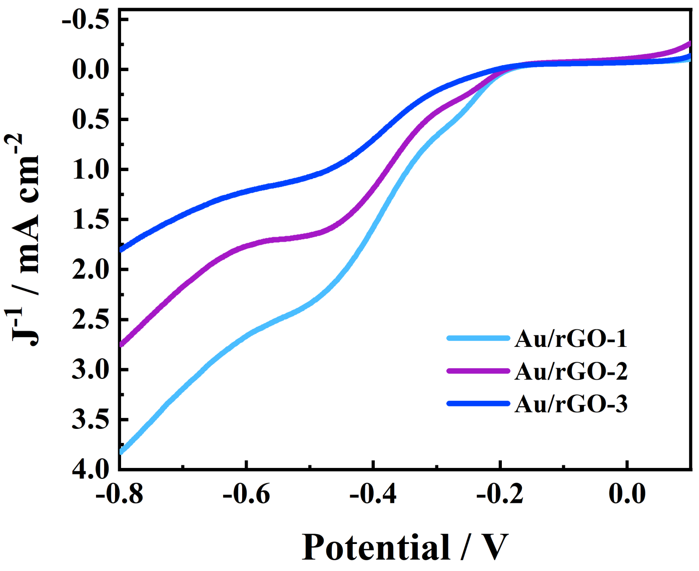


**Figure S8.** RDE lines of Au/rGO-1, Au/rGO-2, and Au/rGO-3 in $O_2$-saturated 0.1M KOH at a scanning rate of 50 mV/s at 1600 rpm.

The behavior of several other noble metal NP (Ag, Pt)/rGO electrocatalysts was investigated. The synthesis of these substances was comparable to that of Au/rGO, as explained in Supporting Information. Figure S9(a) demonstrates that the ORR onset potential of Au/rGO-2 (−0.20 V) is more negative than that of Ag/rGO (−0.15 V) and Pt/rGO (−0.10 V), indicating that the ORR catalytic performance of Au/rGO is relatively weak. Figure S9(b) compares the ECL performance of the metal NP/graphene as the co-reactant of $Ru(bpy)_3^{2+}$. The superior cathodic co-reactant properties of Au/rGO-2 can be explained by its moderate ORR catalytic activity.

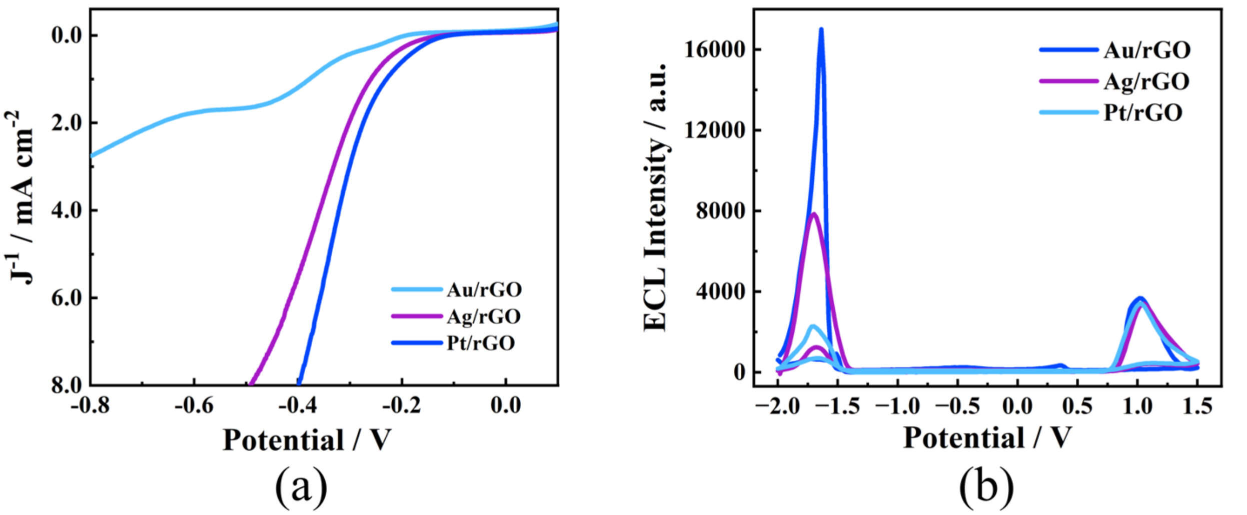


**Figure S9.** (a) RDE lines in $O_2$-saturated 0.1 M KOH at a scanning rate of 50 mV/s at 1600 rpm, and (b) ECL line in 0.1 M $Ru(bpy)_3^{2+}$ of Au/rGO, Ag/rGO, and Pt/rGO.

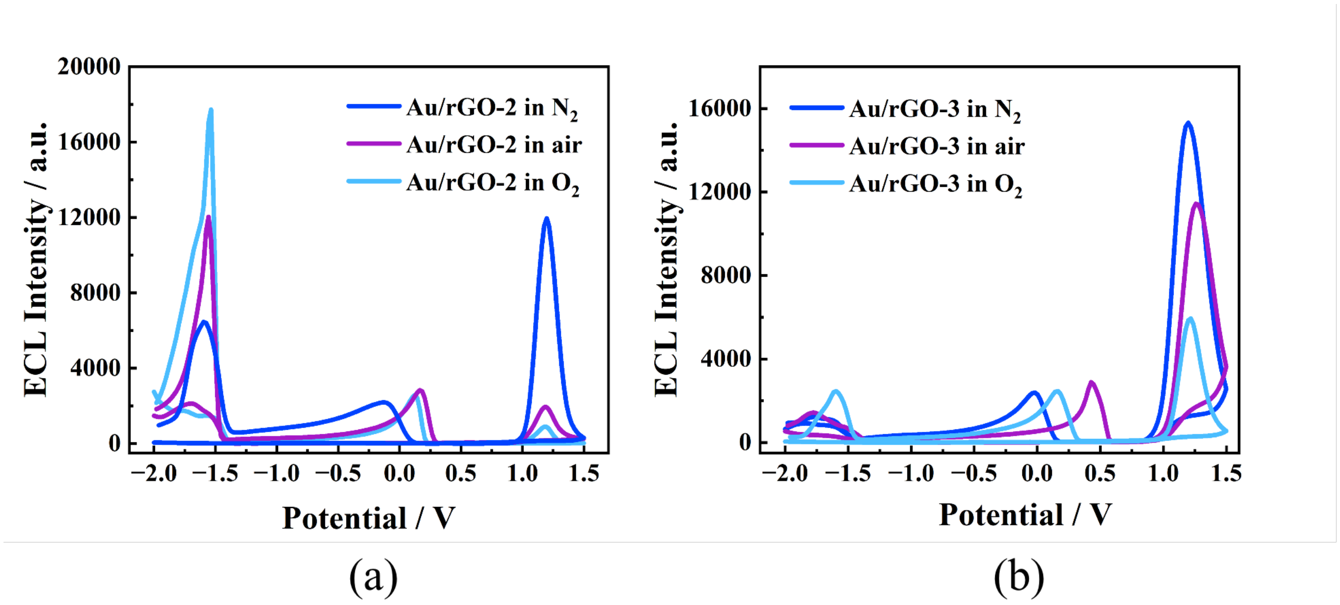


**Figure S10.** The comparison of the ECL performance of (a) Au/rGO-2 and (b) Au/rGO-3 modified on GCE in 0.1 M $Ru(bpy)_3^{2+}$ under the $O_2$, air and $N_2$ atmospheres.

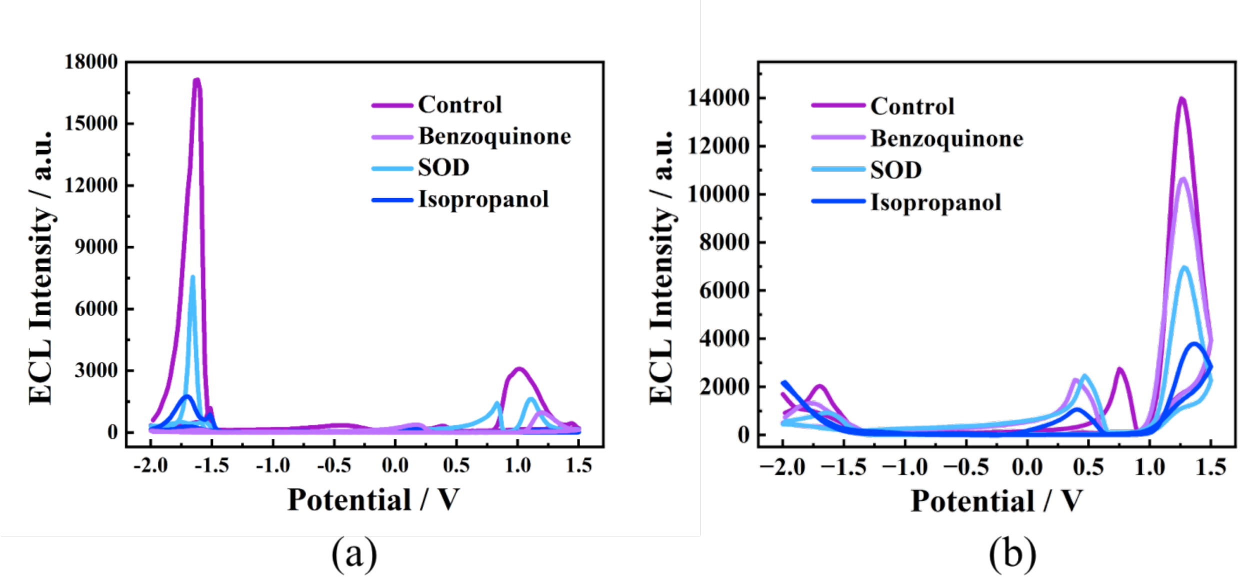


**Figure S11.** ECL responses of (a) Au/rGO-2/GCE and (b) Au/rGO-3/GCE in 0.1 M $Ru(bpy)_3^{2+}$ containing 0.05 mM benzoquinone (light purple line), 0.05 mM SOD (light blue line), 0.05 mM isopropanol (dark blue line) and control group.

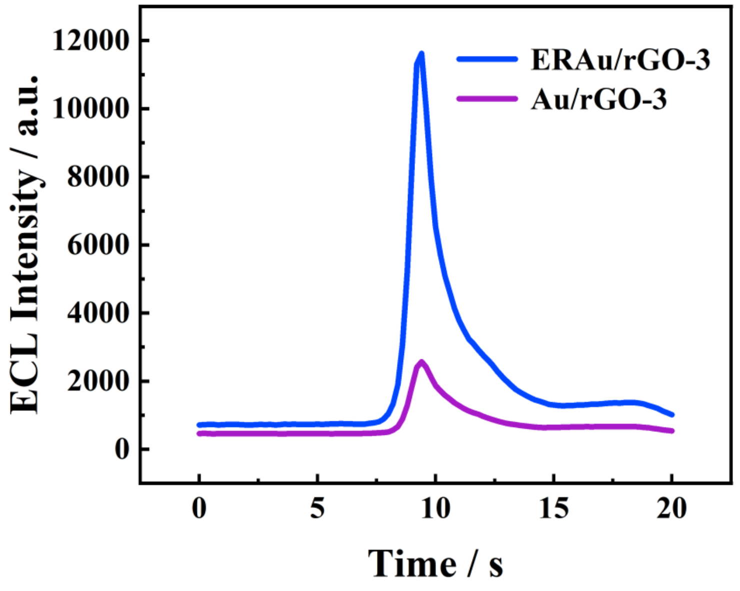


**Figure S12.** ECL curves of Au/rGO-3/GCE in 1 mM $Ru(bpy)_3^{2+}$ before and after Au/rGO-3 was electrochemical reduced (ER) at -1.0 V for 20 minutes.

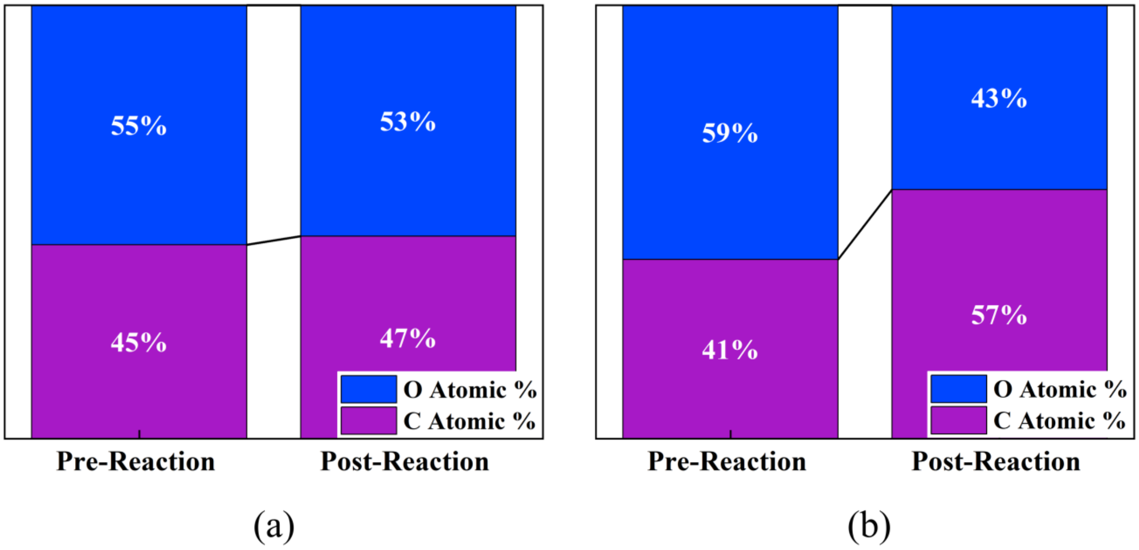


**Figure S13.** Ratio of oxygen and carbon atoms to the total amount of carbon and oxygen atoms of (a) Au/rGO-2 (b) Au/rGO-3 on the GCE electrode before and after a 1 hour reaction with 1 mM $Ru(bpy)_3^{2+}$ (measured by XPS).

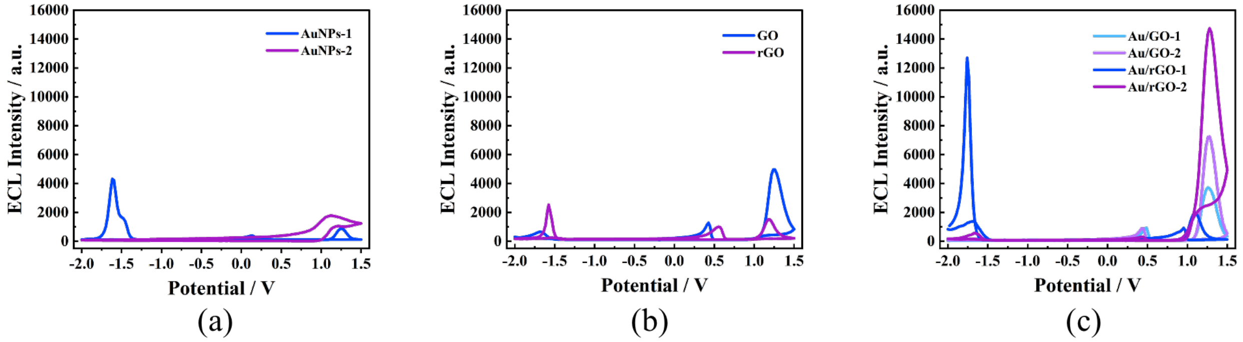


**Figure S14.** ECL performance of (a) AuNPs-1 (with the diameter of about 3 nm, dark blue line) and AuNPs-2 (with the diameter of about 13 nm, dark purple line), (b) GO (dark blue line) and rGO (dark purple line), (c) Au/GO-1 (with AuNPs' diameter of about 3 nm, light blue line), Au/GO-2 (with AuNPs' diameter of about 13 nm, light purple line), Au/rGO-1 (with AuNPs'

diameter of about 3 nm, dark blue line), and Au/rGO-2 (with AuNPs' diameter of about 13 nm, dark purple line) modified on GCE in 0.1 M $Ru(bpy)_3^{2+}$.

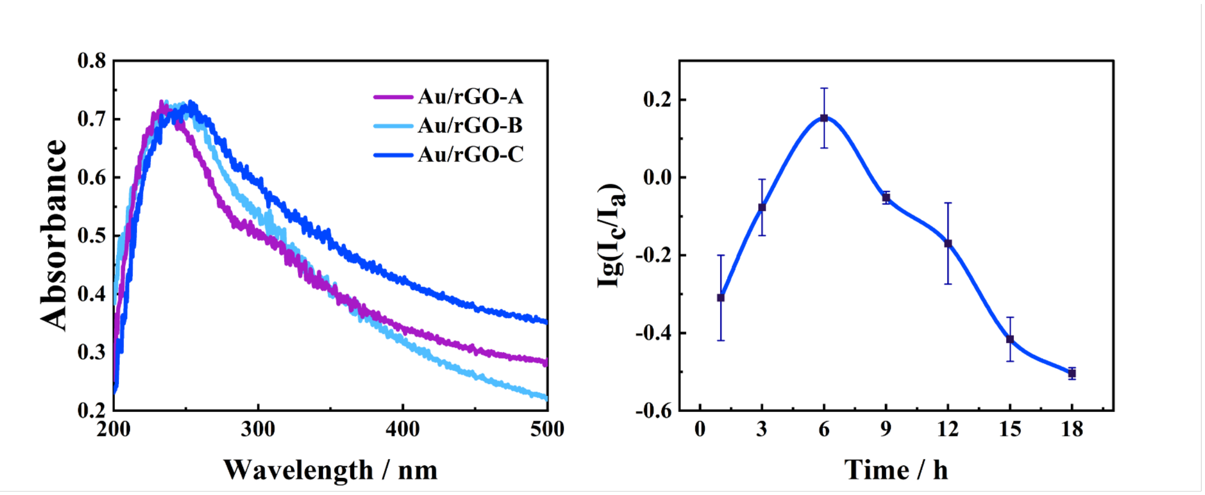


(a) (b)

**Figure S15.** (a) UV-vis absorption spectra of Au/rGO-A (reduce GO by hydrazine hydrate for 2h) (purple line), Au/rGO-B (reduce GO by hydrazine hydrate for 6h) (light blue line), and Au/rGO-C (reduce GO by hydrazine hydrate for 15h) (dark blue line). (b) The logarithm of the cathodic to anodic ECL luminescence intensity of 0.1 M $Ru(bpy)_3^{2+}$ reacting with Au/rGO on the electrode surface with different rGO reduction times.

In the single-luminescent ratiometric ECL immunosensor proposed in this study, the cathode accelerators Au/rGO-2 compete with anode promoter Au/rGO-3 for $Ru(bpy)_3^{2+}$ consumption. So, an improper label-to-substrate concentration ratio and excessively high or low $Ru(bpy)_3^{2+}$'s concentration would impede the inversion of anode to cathodic luminescence from being effectively achieved with the introduction of antigens and Au/rGO-2. This renders the $I_c/I_a$ detection value too minimal, which is detrimental to the sensor's sensitivity and detection limit. Consequently, the goal of this optimization experiment is to identify the optimal $C_{[Au/rGO-2]}/C_{[Au/rGO-3]}$ and concentrations of $Ru(bpy)_3^{2+}$ that yield the maximal immunosensor's output value $lg(I_c/I_a)$.

For the optimization of $C_{[Au/rGO-2]}/C_{[Au/rGO-3]}$, when the concentration of Au/rGO-2 is relatively low, the majority of $Ru(bpy)_3^{2+}$ in the solution is consumed by Au/rGO-3, and the cathodic luminescence effect of Au/rGO-2 reacting with $Ru(bpy)_3^{2+}$ is not readily apparent, resulting in a low $lg(I_c/I_a)$. With an increase in $C_{[Au/rGO-2]}/C_{[Au/rGO-3]}$, the fraction of $Ru(bpy)_3^{2+}$ interacting with Au/rGO-2 rises, and the introduction of a modest number of antigens is reflected clearly by an increase in cathode signal. The output signal of $lg(I_c/I_a)$ nearly peaks until the concentrations of Au/rGO-2 and Au/rGO-3 are equal, that is, until $C_{[Au/rGO-2]}/C_{[Au/rGO-3]} = 1$. When $C_{[Au/rGO-2]}/C_{[Au/rGO-3]}$ further increases, the elevation of $lg(I_c/I_a)$ is no longer obvious, as the reaction between Au/rGO-2 and $Ru(bpy)_3^{2+}$ has reached the limit of chemical reaction, and the excess Au/rGO-2 has little effect on the promotion of cathode signal. Worst still, an excessively high concentration of Au/rGO-2 may result in premature anode-to-cathode inversion of $Ru(bpy)_3^{2+}$'s luminescence as the concentration of biomarker rises. This will result in the cathode signal being excessively high and easily surpasses the range when detecting a high concentration of antigen, which is not favorable to achieving a wider detection limit. Based on the above, the optimal label-to-substrate concentration ratio is determined to be $C_{[Au/rGO-2]}/C_{[Au/rGO-3]}=1$ when $lg(I_c/I_a)$ approaches the inflection point to the maximum value.

For the optimization of $Ru(bpy)_3^{2+}$'s concentration, due to competition between the two luminescence promoters Au/rGO-2 and 3, a rise in $Ru(bpy)_3^{2+}$ concentration allows more Au/rGO-2 distant from the electrode surface to react with $Ru(bpy)_3^{2+}$, leading to a rapid increase in $lg(I_c/I_a)$. When $Ru(bpy)_3^{2+}$ concentration is 1 mM, $lg(I_c/I_a)$ achieves its highest value. As the concentration of $Ru(bpy)_3^{2+}$ continues to rise, the co-reactant pathways of Au/rGO-2 and 3 that promote $Ru(bpy)_3^{2+}$'s luminescence reach their maximum, and the excess $Ru(bpy)_3^{2+}$ emits light via the annihilation pathway, which is primarily anodic luminescence, causing the $lg(I_c/I_a)$ value to fall. Therefore, the ideal luminous concentration was determined to be 1 mM of $Ru(bpy)_3^{2+}$, which occurs at maximal $lg(I_c/I_a)$.

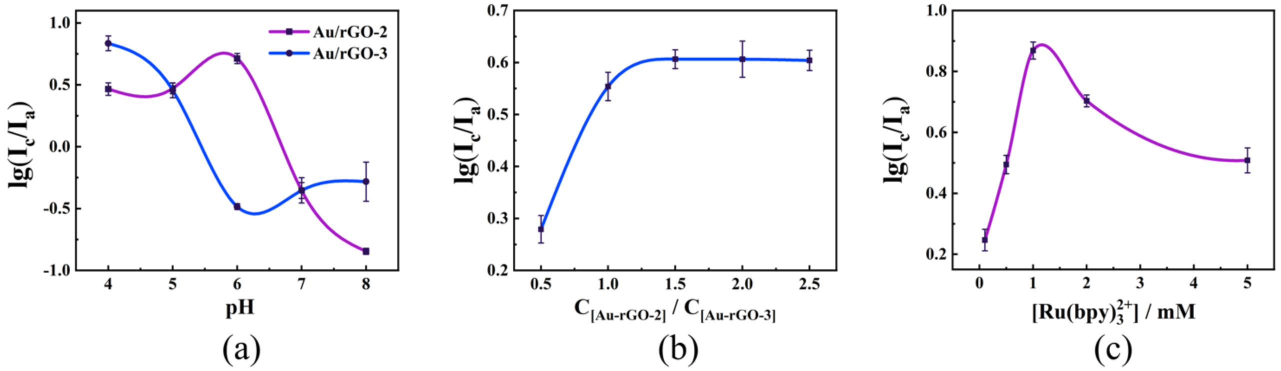


**Figure S16.** The effect of (a) detection pH, (b) concentration ratio of Au/rGO-2 and Au/rGO-3 modified on the substrates and labels of the immunosensor, and (c) concentration of $Ru(bpy)_3^{2+}$ on $lg(I_c/I_a)$ signal output of the immunosensor. When changing a parameter, the others are setting at their optimal conditions.

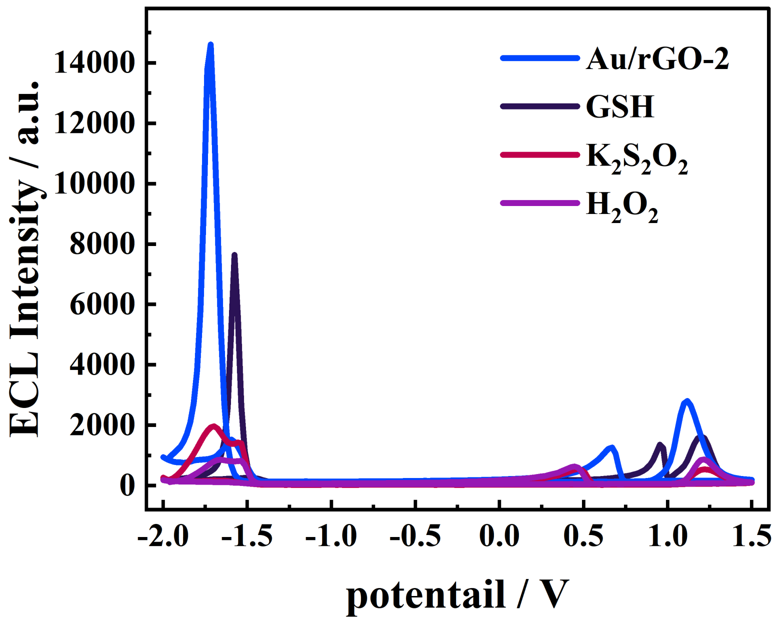


**Figure S17.** The comparison of the cathodic ECL performance of $Ru(bpy)_3^{2+}$ with Au/rGO-2 (blue line) and traditional $Ru(bpy)_3^{2+}$'s cathodic co-reactant GSH (black line), $K_2S_2O_2$(red line), $H_2O_2$ (purple line).

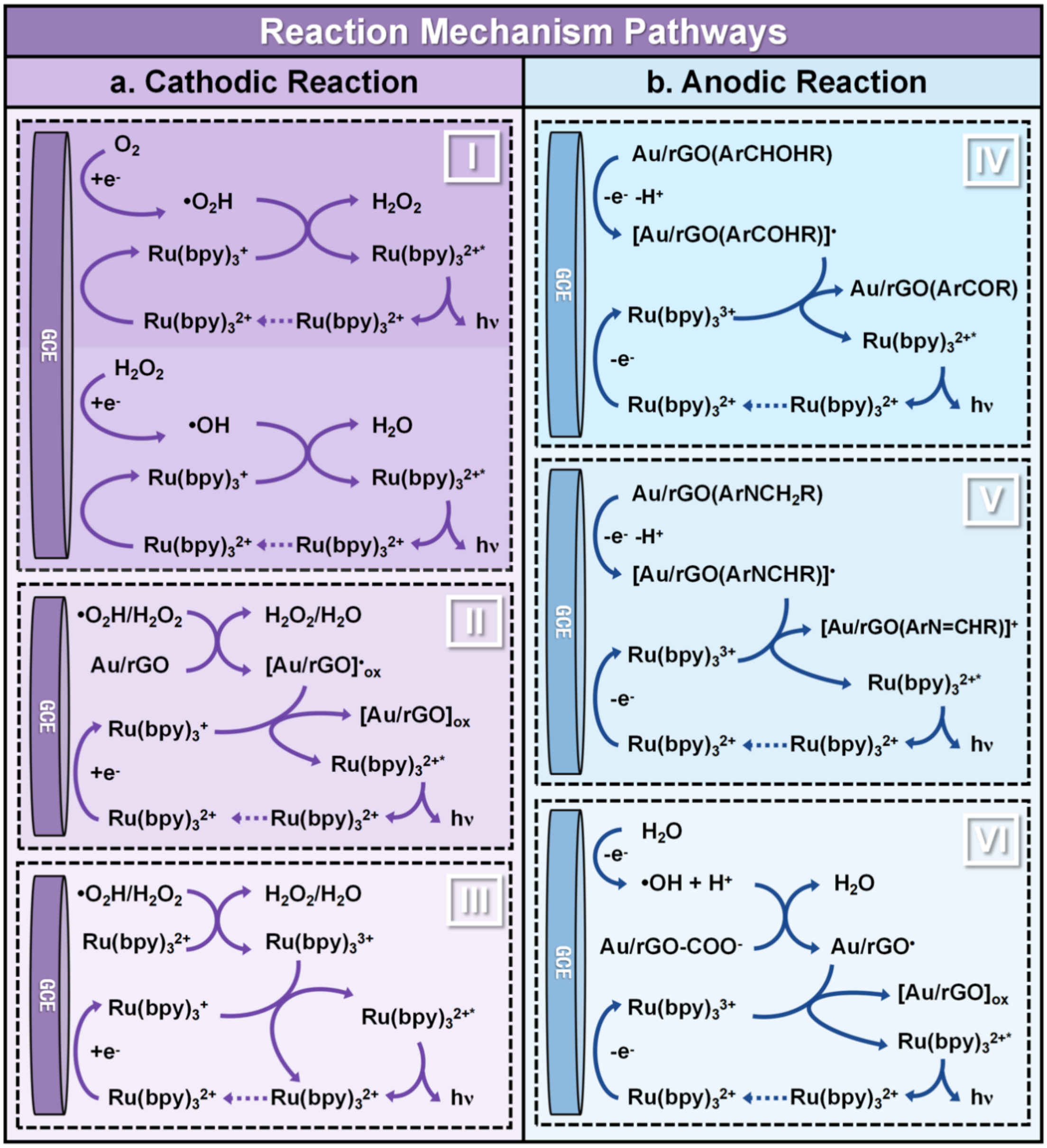


**Scheme S1.** The schematic illustration for reaction mechanism pathways of the Au/rGO-2/3. (a) cathodic ECL generation on Au/rGO-2/GCE and (b) anodic ECL generation on Au/rGO-3/GCE in $Ru(bpy)_3^{2+}$ with PBS at pH 6.

**Table S1.** Comparison of the different potential-resolved platforms for ratiometric ECL immunoassay.

| Detection strategy | Luminophores | Linear range | LOD | Target | Ref. |
|---|---|---|---|---|---|
| ECL-RET strategy | BODIPY/$K_2S_2O_8$ | $1.0 \times 10^{-13}$–$8.5 \times 10^{-7}$g/mL | $4.2 \times 10^{-14}$g/mL | Lactoferrin | [17] |
| | $CeO_2$ – AuNPs – g-CNQDs | $1.0 \times 10^{-14}$–$5.0 \times 10^{-8}$ g/mL | $4.5 \times 10^{-15}$ g/mL | PSA | [18] |
| Competition strategy | CdTe QDs/ABEI | $1.0 \times 10^{-13}$–$1.0 \times 10^{-8}$ g/mL | $3.0 \times 10^{-14}$ g/mL | Concanavalin A | [19] |
| | DBAE/lucigenin | $1.0 \times 10^{-14}$–$1.0 \times 10^{-8}$ g/ml | $3.3 \times 10^{-15}$ g/mL | Human epididymis protein 4 | [20] |
| Internal standard strategy | $Ru(bpy)_3^{2+}$@RuSi NP/SQD | $1.0 \times 10^{-13}$–$1.0 \times 10^{-7}$ g/mL | $5.0 \times 10^{-14}$ g/mL | myoglobin | [21] |
| | Ferrocene/$[Ru(NH_3)_6]^{3+}$ | $1.0 \times 10^{-12}$–$1.0 \times 10^{-6}$ g/mL | $6.2 \times 10^{-13}$ g/mL | CEA | [22] |
| single luminophore strategy | g-$C_3N_4$ | $3.0 \times 10^{-16}$–$1.0 \times 10^{-11}$ g/mL | $1.0 \times 10^{-16}$ g/mL | AFP | [23] |
| | $Ru(bpy)_3^{2+}$ | $1.0 \times 10^{-16}$–$1.0 \times 10^{-10}$ g/mL | $3.3 \times 10^{-17}$ g/mL | CEA | This work |

Abbreviations: BODIPY: Fluoroboron dipyrrole; $K_2S_2O_8$: potassium persulfate; $CeO_2$–AuNPs–g-CNQDs: nano-$CeO_2$ and AuNPs decorated graphitic carbon nitride quantum dots; g-$C_3N_4$: graphite-like carbon nitride; CdTe QDs: CdTe Quantum Dots; ABEI: N-(4-Aminobutyl)-N-ethylisoluminol; DBAE: 2-(Butylamino)ethanol; RuSiNP/SQD: $Ru(bpy)_3^{2+}$-conjugated silica nanoparticles.

**Table S2.** The XPS atomic of C1s, N1s, O1s and Au4f on Au/rGO synthesized at different concentrations of $HAuCl_4$

| $HAuCl_4$ Concentrations (mM) | Atomic of C1s (%) | Atomic of N1s (%) | Atomic of O1s (%) | Atomic of Au4f (%) |
|---|---|---|---|---|
| 60 | 69.15 | 2.68 | 28.16 | 0.01 |
| 180 | 69.63 | 2.60 | 27.73 | 0.04 |

## REFERENCES


[1] S.-P. Cao, Q.-X. Luo, Y.-J. Li, R.-P. Liang, J.-D. Qiu, *Chem. Commun.* **2020**, *56*, 5625-5628.
[2] R. A. Masitas, F. P. Zamborini, *Journal of the American Chemical Society* **2012**, *134*, 5014-5017.
[3] M. M. Walczak, D. A. Dryer, D. D. Jacobson, M. G. Foss, N. T. Flynn, *Journal of chemical education* **1997**, *74*, 1195.
[4] L. Di, X. Zhang, B. Lee, P. Lu, W.-S. Ahn, D.-W. Park, *Plasma Chemistry and Plasma Processing* **2017**, *37*, 1535-1549.
[5] W. Xin, I. M. De Rosa, P. Ye, J. Severino, C. Li, X. Yin, M. S. Goorsky, L. Carlson, J.-M. Yang, *Nanoscale* **2018**, *10*, 2764-2773.
[6] I. Roy, A. Bhattacharyya, G. Sarkar, N. R. Saha, D. Rana, P. P. Ghosh, M. Palit, A. R. Das, D. Chattopadhyay, *RSC Advances* **2014**, *4*, 52044-52052.
[7] Y. Wang, S. J. Zhen, Y. Zhang, Y. F. Li, C. Z. Huang, *The Journal of Physical Chemistry C* **2011**, *115*, 12815-12821.
[8] S. Jafarirad, M. Kosari-Nasab, R. M. Tavana, S. Mahjouri, R. Ebadollahi, *Ecotoxicology and Environmental Safety* **2021**, *209*, 111841.
[9] R.-P. Liang, L.-D. Yu, Y.-J. Tong, S.-H. Wen, S.-P. Cao, J.-D. Qiu, *Chem. Commun.* **2018**, *54*, 14001-14004.
[10] T. Fan, W. Zeng, W. Tang, C. Yuan, S. Tong, K. Cai, Y. Liu, W. Huang, Y. Min, A. J. Epstein, *Nanoscale research letters* **2015**, *10*, 1-8.
[11] Z. Zhou, Q. Shang, Y. Shen, L. Zhang, Y. Zhang, Y. Lv, Y. Li, S. Liu, Y. Zhang, *Analytical chemistry* **2016**, *88*, 6004-6010.
[12] F. Ren, C. Wang, C. Zhai, F. Jiang, R. Yue, Y. Du, P. Yang, J. Xu, *Journal of Materials Chemistry A* **2013**, *1*, 7255-7261.
[13] Q.-M. Feng, Y.-Z. Shen, M.-X. Li, Z.-L. Zhang, W. Zhao, J.-J. Xu, H.-Y. Chen, *Analytical chemistry* **2016**, *88*, 937-944.
[14] H. Li, Z. Bian, J. Zhu, Y. Huo, H. Li, Y. Lu, *Journal of the American Chemical Society* **2007**, *129*, 4538-4539.
[15] K. Subrahmanyam, A. K. Manna, S. K. Pati, C. Rao, *Chemical Physics Letters* **2010**, *497*, 70-75.
[16] G. Goncalves, P. A. Marques, C. M. Granadeiro, H. I. Nogueira, M. Singh, J. Gracio, *Chemistry of Materials* **2009**, *21*, 4796-4802.
[17] Y. Lu, H. Ke, Y. Wang, Y. Zhang, H. Li, C. Huang, N. Jia, *Biosensors and Bioelectronics* **2020**, *170*, 112664.
[18] Z. Liu, J. Wang, C. Cui, L. Zheng, L. Hu, *Talanta* **2022**, 123886.
