# Supplementary figures and images for: Particle Size-Controlled Oxygen Reduction and Evolution Reaction Nanocatalysts Regulate Ru(bpy)32+’s Dual-potential Electrochemiluminescence for Sandwich Immunoassay
Source: Research (Wash D C). 2023 Apr 14;6:0117. doi: 10.34133/research.0117 (PMC10243198; doi:10.34133/research.0117)

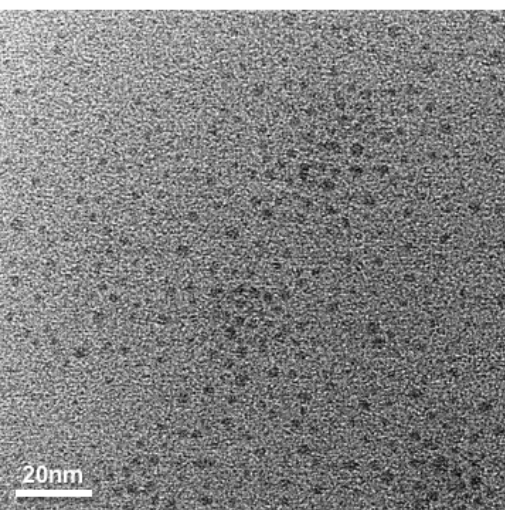

(a)

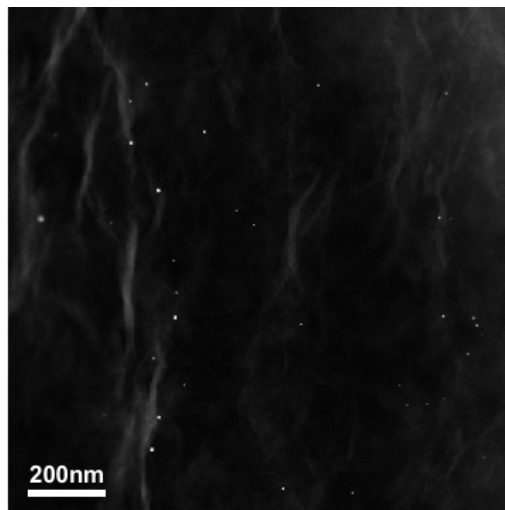

(b)

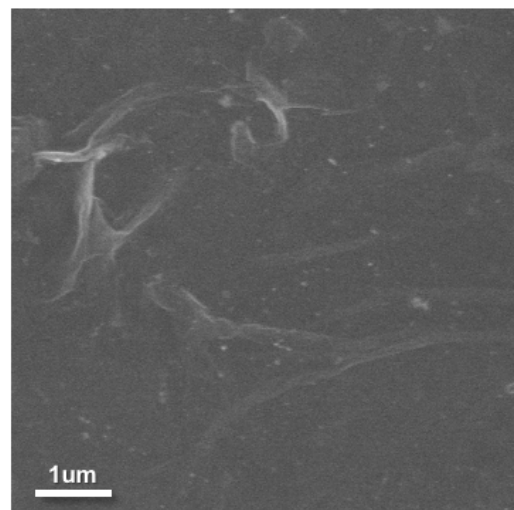

(c)

Supplement: Supplementary 1 — Fig. S1. (A) TEM image, (B) HAADF image, and (C) SEM image of Au/rGO. Fig. S2. DPV lines of rGO and Au/rGO on GCE in PBS. Fig. S3. EDS mappings of Au/rGO. Fig. S4. XRD patterns of rGO and Au/rGOs. Fig. S5. Zeta potential of GO, rGO, and Au/rGO. Fig. S6. XPS survey spectra of rGO (A), Au/rGO (B), C1s deconvolution spectrum N1s peak (C), and Au4f region of Au/rGO (D). Fig. S7. Raman spectrum of rGO and Au/rGOs. Fig. S8. RDE lines of Au/rGO-1, Au/rGO-2, and Au/rGO-3 in O2-saturated 0.1 M KOH. Fig. S9. (A) RDE lines in O2-saturated 0.1 M KOH at 1,600 rpm and (B) ECL line in 0.1 M Ru(bpy)32+ of Au/rGO, Ag/rGO, and Pt/rGO. Fig. S10. ECL performance of Au/rGO-2 and Au/rGO-3 under O2, air, and N2 atmospheres. Fig. S11. ECL responses of Au/rGO-2 and Au/rGO-3 in Ru(bpy)32+ with ROS inhibitor BQ, SOD, and isopropanol. Fig. S12. ECL curves of Au/rGO-3/GCE in Ru(bpy)32+ before and after it was electrochemically reduced. Fig. S13. Oxygen and carbon atoms ratio of (A) Au/rGO-2 and (B) Au/rGO-3 before and after the reaction with Ru(bpy)32+. Fig. S14. ECL performance of AuNPs with different diameters (A), GO and rGO (B), and Au/GO-1 and Au/GO-2 as well as Au/rGO-1 and Au/rGO-2 (C) in Ru(bpy)32+. Fig. S15. Ultraviolet-visible absorption spectra of Au/rGO with different GO reduction degrees (A). The logarithm of the anodic to cathodic ECL luminescence intensity of Ru(bpy)32+ reacting with Au/rGO with different rGO reduction times (B). Fig. S16. The effect of (A) pH, (B) C[Au/rGO-2]/C[Au/rGO-3], and (C) Ru(bpy)32+’s concentration on lg(Ic/Ia) signal output of the immunosensor. Fig. S17. The comparison of Au/rGO-2 with traditional Ru(bpy)32+’s cathodic co-reactant GSH, K2S2O8, and H2O2. Scheme S1. The schematic illustration for cathodic and anodic ECL reaction pathways. Table S1. Comparison of the different potential-resolved platforms for ratiometric ECL immunoassay. Table S2. The XPS atomic of C1s, N1s, O1s, and Au4f on Au/rGO synthesized at different concentrations of HAuCl4 [file research.0117.f1.zip › Figure S1.pdf]

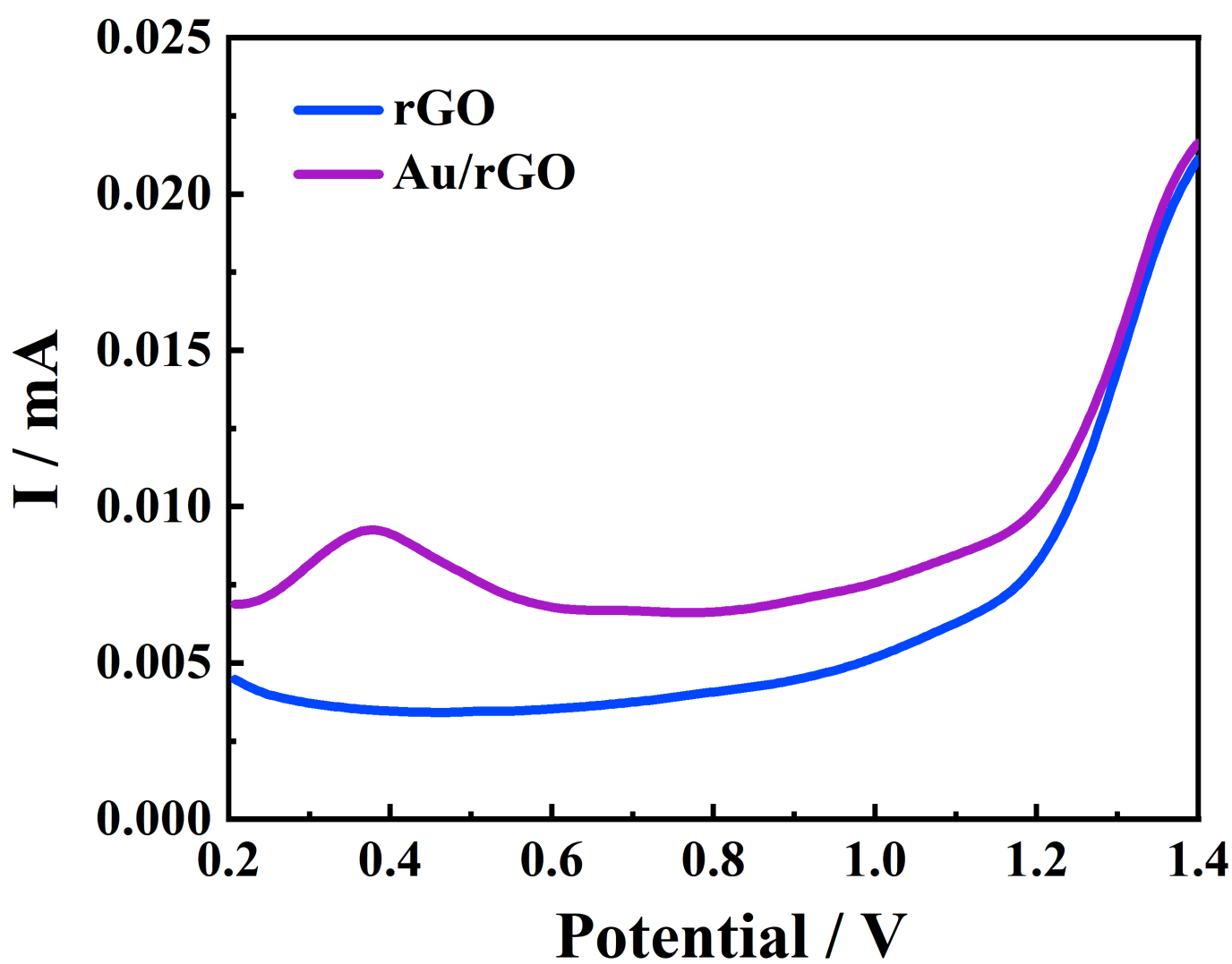

Supplement: Supplementary 1 — Fig. S1. (A) TEM image, (B) HAADF image, and (C) SEM image of Au/rGO. Fig. S2. DPV lines of rGO and Au/rGO on GCE in PBS. Fig. S3. EDS mappings of Au/rGO. Fig. S4. XRD patterns of rGO and Au/rGOs. Fig. S5. Zeta potential of GO, rGO, and Au/rGO. Fig. S6. XPS survey spectra of rGO (A), Au/rGO (B), C1s deconvolution spectrum N1s peak (C), and Au4f region of Au/rGO (D). Fig. S7. Raman spectrum of rGO and Au/rGOs. Fig. S8. RDE lines of Au/rGO-1, Au/rGO-2, and Au/rGO-3 in O2-saturated 0.1 M KOH. Fig. S9. (A) RDE lines in O2-saturated 0.1 M KOH at 1,600 rpm and (B) ECL line in 0.1 M Ru(bpy)32+ of Au/rGO, Ag/rGO, and Pt/rGO. Fig. S10. ECL performance of Au/rGO-2 and Au/rGO-3 under O2, air, and N2 atmospheres. Fig. S11. ECL responses of Au/rGO-2 and Au/rGO-3 in Ru(bpy)32+ with ROS inhibitor BQ, SOD, and isopropanol. Fig. S12. ECL curves of Au/rGO-3/GCE in Ru(bpy)32+ before and after it was electrochemically reduced. Fig. S13. Oxygen and carbon atoms ratio of (A) Au/rGO-2 and (B) Au/rGO-3 before and after the reaction with Ru(bpy)32+. Fig. S14. ECL performance of AuNPs with different diameters (A), GO and rGO (B), and Au/GO-1 and Au/GO-2 as well as Au/rGO-1 and Au/rGO-2 (C) in Ru(bpy)32+. Fig. S15. Ultraviolet-visible absorption spectra of Au/rGO with different GO reduction degrees (A). The logarithm of the anodic to cathodic ECL luminescence intensity of Ru(bpy)32+ reacting with Au/rGO with different rGO reduction times (B). Fig. S16. The effect of (A) pH, (B) C[Au/rGO-2]/C[Au/rGO-3], and (C) Ru(bpy)32+’s concentration on lg(Ic/Ia) signal output of the immunosensor. Fig. S17. The comparison of Au/rGO-2 with traditional Ru(bpy)32+’s cathodic co-reactant GSH, K2S2O8, and H2O2. Scheme S1. The schematic illustration for cathodic and anodic ECL reaction pathways. Table S1. Comparison of the different potential-resolved platforms for ratiometric ECL immunoassay. Table S2. The XPS atomic of C1s, N1s, O1s, and Au4f on Au/rGO synthesized at different concentrations of HAuCl4 [file research.0117.f1.zip › Figure S2.pdf]

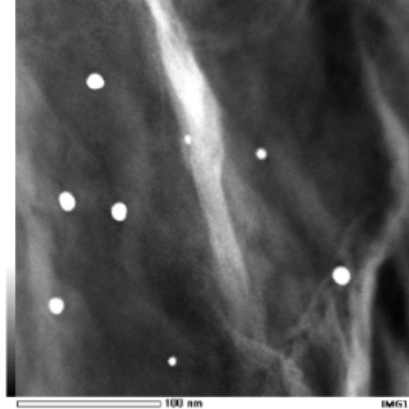

(a)

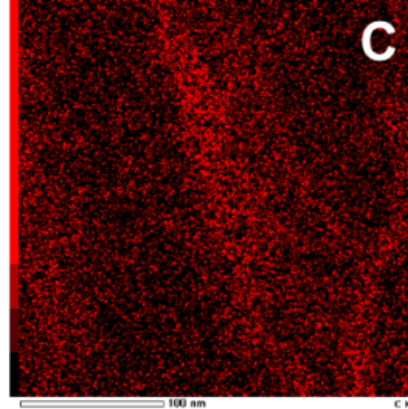

(b)

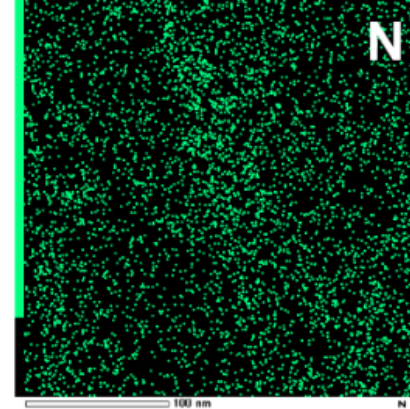

(c)

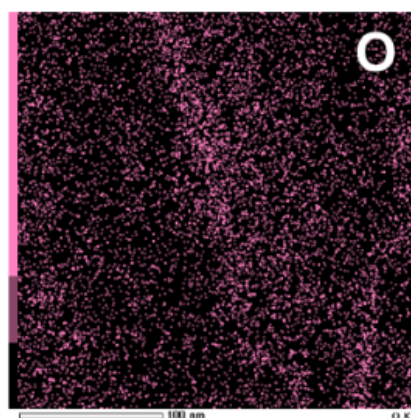

(d)

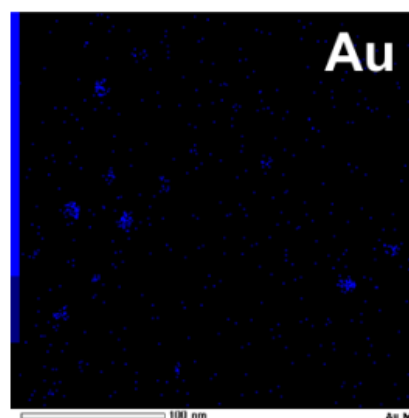

(e)

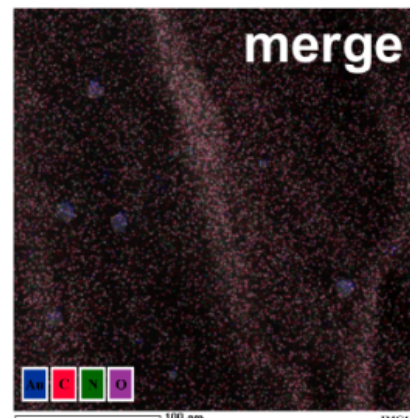

(f)

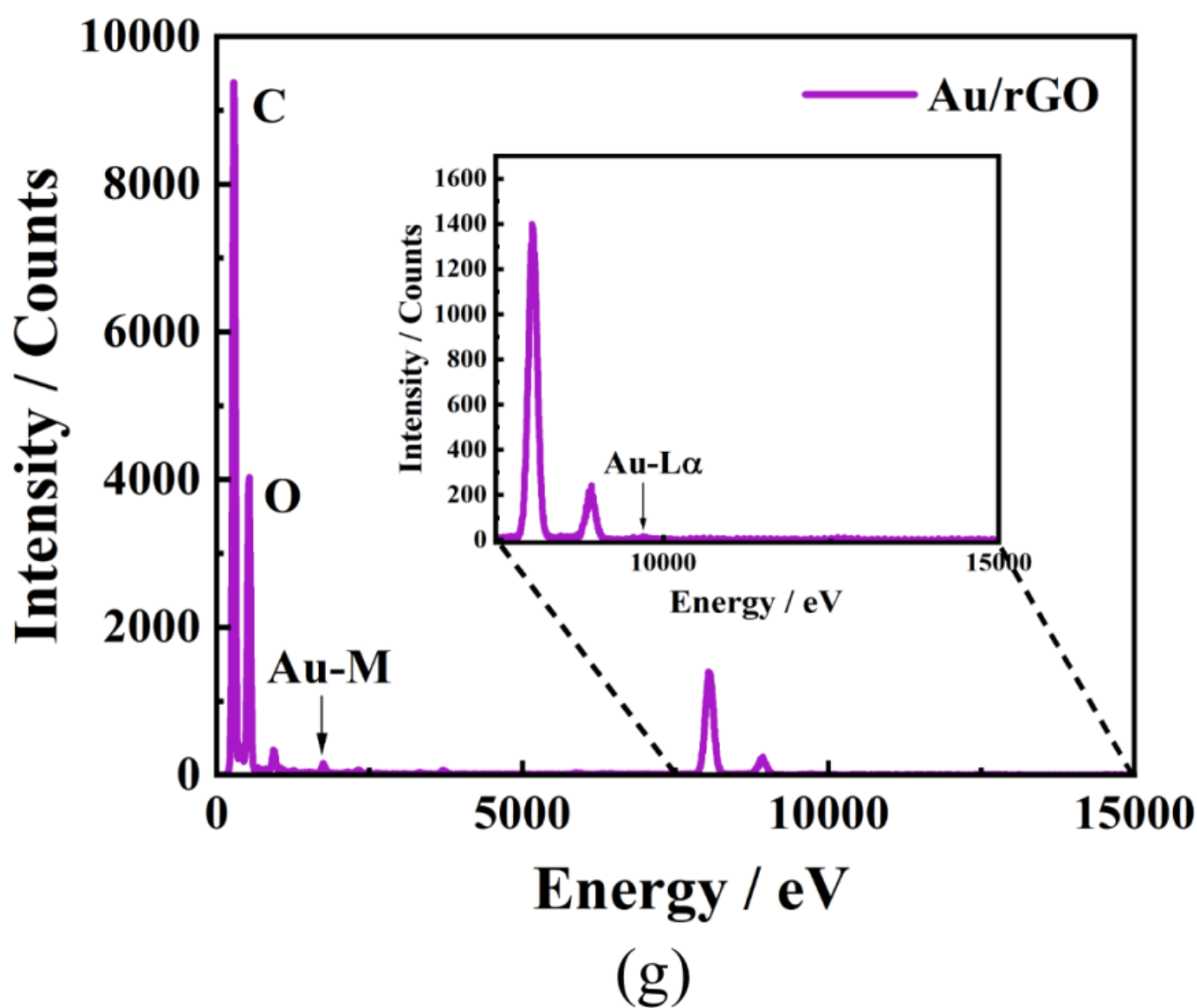

Supplement: Supplementary 1 — Fig. S1. (A) TEM image, (B) HAADF image, and (C) SEM image of Au/rGO. Fig. S2. DPV lines of rGO and Au/rGO on GCE in PBS. Fig. S3. EDS mappings of Au/rGO. Fig. S4. XRD patterns of rGO and Au/rGOs. Fig. S5. Zeta potential of GO, rGO, and Au/rGO. Fig. S6. XPS survey spectra of rGO (A), Au/rGO (B), C1s deconvolution spectrum N1s peak (C), and Au4f region of Au/rGO (D). Fig. S7. Raman spectrum of rGO and Au/rGOs. Fig. S8. RDE lines of Au/rGO-1, Au/rGO-2, and Au/rGO-3 in O2-saturated 0.1 M KOH. Fig. S9. (A) RDE lines in O2-saturated 0.1 M KOH at 1,600 rpm and (B) ECL line in 0.1 M Ru(bpy)32+ of Au/rGO, Ag/rGO, and Pt/rGO. Fig. S10. ECL performance of Au/rGO-2 and Au/rGO-3 under O2, air, and N2 atmospheres. Fig. S11. ECL responses of Au/rGO-2 and Au/rGO-3 in Ru(bpy)32+ with ROS inhibitor BQ, SOD, and isopropanol. Fig. S12. ECL curves of Au/rGO-3/GCE in Ru(bpy)32+ before and after it was electrochemically reduced. Fig. S13. Oxygen and carbon atoms ratio of (A) Au/rGO-2 and (B) Au/rGO-3 before and after the reaction with Ru(bpy)32+. Fig. S14. ECL performance of AuNPs with different diameters (A), GO and rGO (B), and Au/GO-1 and Au/GO-2 as well as Au/rGO-1 and Au/rGO-2 (C) in Ru(bpy)32+. Fig. S15. Ultraviolet-visible absorption spectra of Au/rGO with different GO reduction degrees (A). The logarithm of the anodic to cathodic ECL luminescence intensity of Ru(bpy)32+ reacting with Au/rGO with different rGO reduction times (B). Fig. S16. The effect of (A) pH, (B) C[Au/rGO-2]/C[Au/rGO-3], and (C) Ru(bpy)32+’s concentration on lg(Ic/Ia) signal output of the immunosensor. Fig. S17. The comparison of Au/rGO-2 with traditional Ru(bpy)32+’s cathodic co-reactant GSH, K2S2O8, and H2O2. Scheme S1. The schematic illustration for cathodic and anodic ECL reaction pathways. Table S1. Comparison of the different potential-resolved platforms for ratiometric ECL immunoassay. Table S2. The XPS atomic of C1s, N1s, O1s, and Au4f on Au/rGO synthesized at different concentrations of HAuCl4 [file research.0117.f1.zip › Figure S3.pdf]

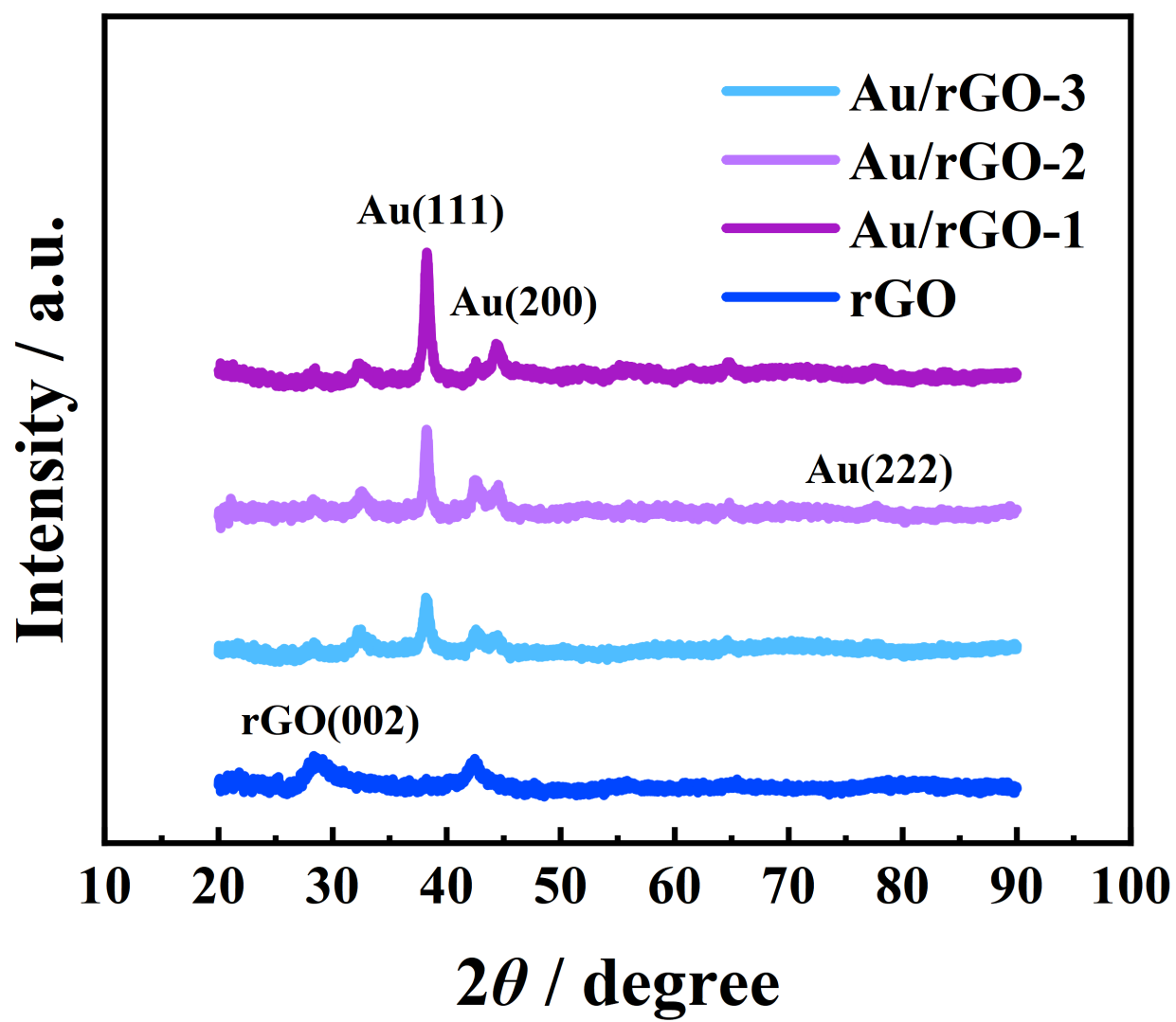

Supplement: Supplementary 1 — Fig. S1. (A) TEM image, (B) HAADF image, and (C) SEM image of Au/rGO. Fig. S2. DPV lines of rGO and Au/rGO on GCE in PBS. Fig. S3. EDS mappings of Au/rGO. Fig. S4. XRD patterns of rGO and Au/rGOs. Fig. S5. Zeta potential of GO, rGO, and Au/rGO. Fig. S6. XPS survey spectra of rGO (A), Au/rGO (B), C1s deconvolution spectrum N1s peak (C), and Au4f region of Au/rGO (D). Fig. S7. Raman spectrum of rGO and Au/rGOs. Fig. S8. RDE lines of Au/rGO-1, Au/rGO-2, and Au/rGO-3 in O2-saturated 0.1 M KOH. Fig. S9. (A) RDE lines in O2-saturated 0.1 M KOH at 1,600 rpm and (B) ECL line in 0.1 M Ru(bpy)32+ of Au/rGO, Ag/rGO, and Pt/rGO. Fig. S10. ECL performance of Au/rGO-2 and Au/rGO-3 under O2, air, and N2 atmospheres. Fig. S11. ECL responses of Au/rGO-2 and Au/rGO-3 in Ru(bpy)32+ with ROS inhibitor BQ, SOD, and isopropanol. Fig. S12. ECL curves of Au/rGO-3/GCE in Ru(bpy)32+ before and after it was electrochemically reduced. Fig. S13. Oxygen and carbon atoms ratio of (A) Au/rGO-2 and (B) Au/rGO-3 before and after the reaction with Ru(bpy)32+. Fig. S14. ECL performance of AuNPs with different diameters (A), GO and rGO (B), and Au/GO-1 and Au/GO-2 as well as Au/rGO-1 and Au/rGO-2 (C) in Ru(bpy)32+. Fig. S15. Ultraviolet-visible absorption spectra of Au/rGO with different GO reduction degrees (A). The logarithm of the anodic to cathodic ECL luminescence intensity of Ru(bpy)32+ reacting with Au/rGO with different rGO reduction times (B). Fig. S16. The effect of (A) pH, (B) C[Au/rGO-2]/C[Au/rGO-3], and (C) Ru(bpy)32+’s concentration on lg(Ic/Ia) signal output of the immunosensor. Fig. S17. The comparison of Au/rGO-2 with traditional Ru(bpy)32+’s cathodic co-reactant GSH, K2S2O8, and H2O2. Scheme S1. The schematic illustration for cathodic and anodic ECL reaction pathways. Table S1. Comparison of the different potential-resolved platforms for ratiometric ECL immunoassay. Table S2. The XPS atomic of C1s, N1s, O1s, and Au4f on Au/rGO synthesized at different concentrations of HAuCl4 [file research.0117.f1.zip › Figure S4.pdf]

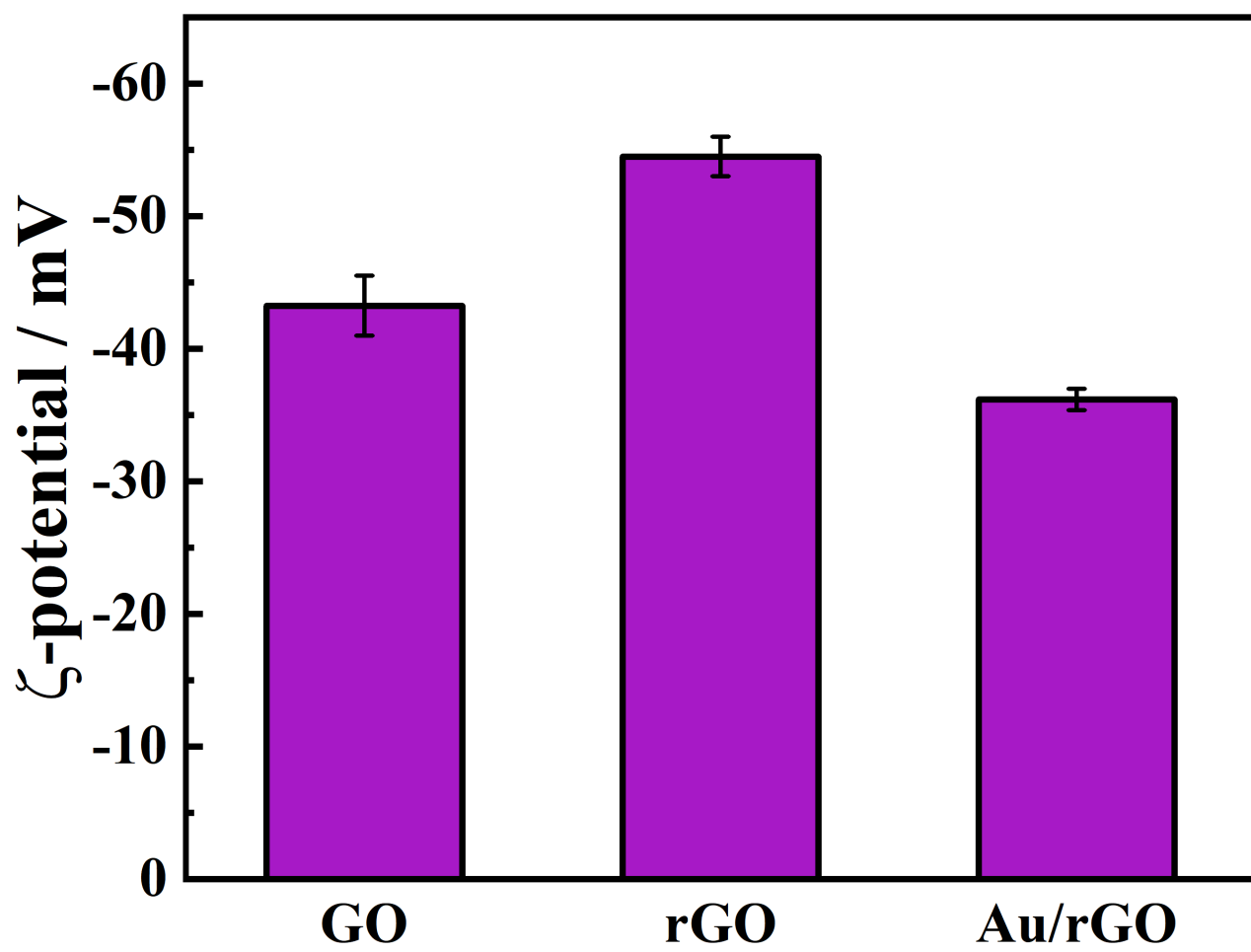

Supplement: Supplementary 1 — Fig. S1. (A) TEM image, (B) HAADF image, and (C) SEM image of Au/rGO. Fig. S2. DPV lines of rGO and Au/rGO on GCE in PBS. Fig. S3. EDS mappings of Au/rGO. Fig. S4. XRD patterns of rGO and Au/rGOs. Fig. S5. Zeta potential of GO, rGO, and Au/rGO. Fig. S6. XPS survey spectra of rGO (A), Au/rGO (B), C1s deconvolution spectrum N1s peak (C), and Au4f region of Au/rGO (D). Fig. S7. Raman spectrum of rGO and Au/rGOs. Fig. S8. RDE lines of Au/rGO-1, Au/rGO-2, and Au/rGO-3 in O2-saturated 0.1 M KOH. Fig. S9. (A) RDE lines in O2-saturated 0.1 M KOH at 1,600 rpm and (B) ECL line in 0.1 M Ru(bpy)32+ of Au/rGO, Ag/rGO, and Pt/rGO. Fig. S10. ECL performance of Au/rGO-2 and Au/rGO-3 under O2, air, and N2 atmospheres. Fig. S11. ECL responses of Au/rGO-2 and Au/rGO-3 in Ru(bpy)32+ with ROS inhibitor BQ, SOD, and isopropanol. Fig. S12. ECL curves of Au/rGO-3/GCE in Ru(bpy)32+ before and after it was electrochemically reduced. Fig. S13. Oxygen and carbon atoms ratio of (A) Au/rGO-2 and (B) Au/rGO-3 before and after the reaction with Ru(bpy)32+. Fig. S14. ECL performance of AuNPs with different diameters (A), GO and rGO (B), and Au/GO-1 and Au/GO-2 as well as Au/rGO-1 and Au/rGO-2 (C) in Ru(bpy)32+. Fig. S15. Ultraviolet-visible absorption spectra of Au/rGO with different GO reduction degrees (A). The logarithm of the anodic to cathodic ECL luminescence intensity of Ru(bpy)32+ reacting with Au/rGO with different rGO reduction times (B). Fig. S16. The effect of (A) pH, (B) C[Au/rGO-2]/C[Au/rGO-3], and (C) Ru(bpy)32+’s concentration on lg(Ic/Ia) signal output of the immunosensor. Fig. S17. The comparison of Au/rGO-2 with traditional Ru(bpy)32+’s cathodic co-reactant GSH, K2S2O8, and H2O2. Scheme S1. The schematic illustration for cathodic and anodic ECL reaction pathways. Table S1. Comparison of the different potential-resolved platforms for ratiometric ECL immunoassay. Table S2. The XPS atomic of C1s, N1s, O1s, and Au4f on Au/rGO synthesized at different concentrations of HAuCl4 [file research.0117.f1.zip › Figure S5.pdf]

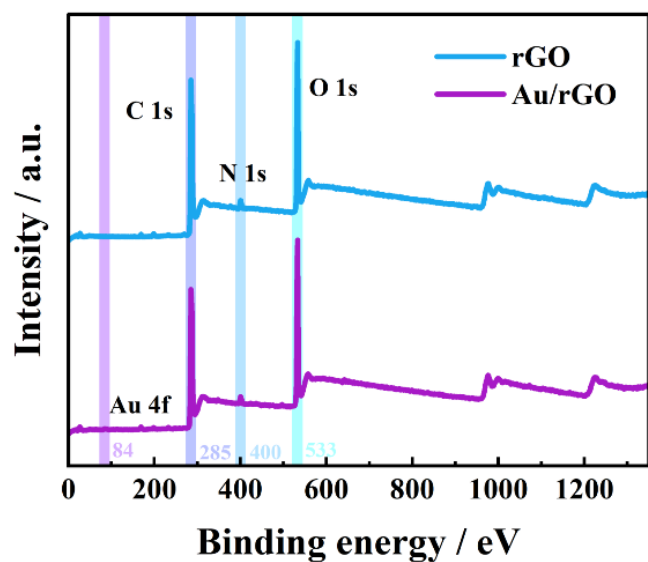

(a)

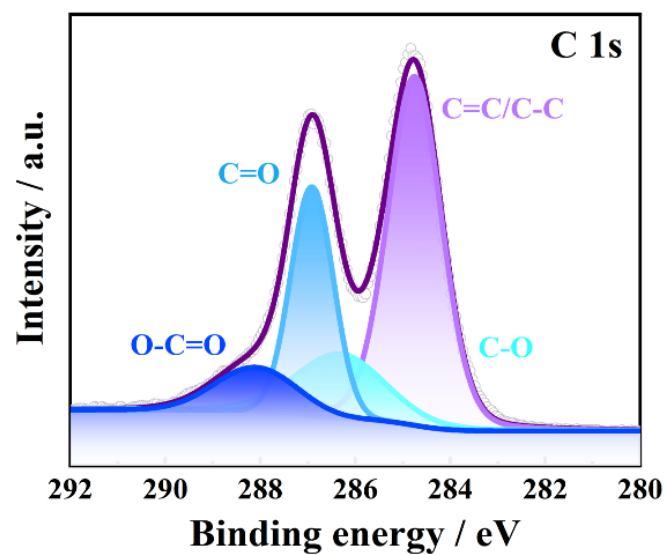

(b)

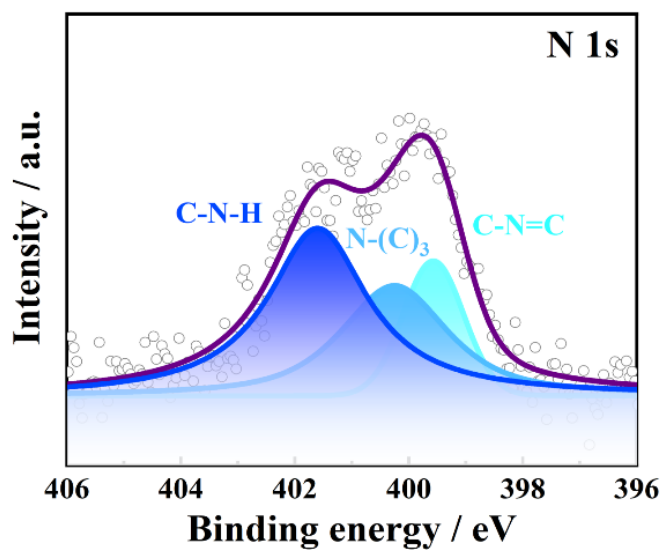

(c)

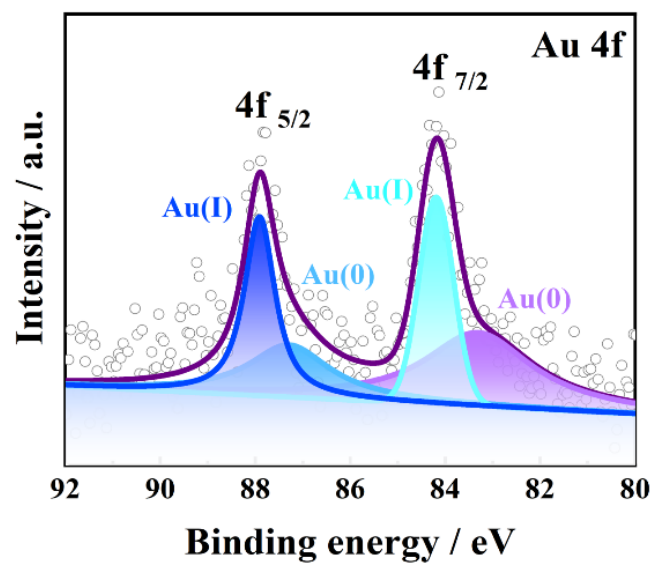

(d)

Supplement: Supplementary 1 — Fig. S1. (A) TEM image, (B) HAADF image, and (C) SEM image of Au/rGO. Fig. S2. DPV lines of rGO and Au/rGO on GCE in PBS. Fig. S3. EDS mappings of Au/rGO. Fig. S4. XRD patterns of rGO and Au/rGOs. Fig. S5. Zeta potential of GO, rGO, and Au/rGO. Fig. S6. XPS survey spectra of rGO (A), Au/rGO (B), C1s deconvolution spectrum N1s peak (C), and Au4f region of Au/rGO (D). Fig. S7. Raman spectrum of rGO and Au/rGOs. Fig. S8. RDE lines of Au/rGO-1, Au/rGO-2, and Au/rGO-3 in O2-saturated 0.1 M KOH. Fig. S9. (A) RDE lines in O2-saturated 0.1 M KOH at 1,600 rpm and (B) ECL line in 0.1 M Ru(bpy)32+ of Au/rGO, Ag/rGO, and Pt/rGO. Fig. S10. ECL performance of Au/rGO-2 and Au/rGO-3 under O2, air, and N2 atmospheres. Fig. S11. ECL responses of Au/rGO-2 and Au/rGO-3 in Ru(bpy)32+ with ROS inhibitor BQ, SOD, and isopropanol. Fig. S12. ECL curves of Au/rGO-3/GCE in Ru(bpy)32+ before and after it was electrochemically reduced. Fig. S13. Oxygen and carbon atoms ratio of (A) Au/rGO-2 and (B) Au/rGO-3 before and after the reaction with Ru(bpy)32+. Fig. S14. ECL performance of AuNPs with different diameters (A), GO and rGO (B), and Au/GO-1 and Au/GO-2 as well as Au/rGO-1 and Au/rGO-2 (C) in Ru(bpy)32+. Fig. S15. Ultraviolet-visible absorption spectra of Au/rGO with different GO reduction degrees (A). The logarithm of the anodic to cathodic ECL luminescence intensity of Ru(bpy)32+ reacting with Au/rGO with different rGO reduction times (B). Fig. S16. The effect of (A) pH, (B) C[Au/rGO-2]/C[Au/rGO-3], and (C) Ru(bpy)32+’s concentration on lg(Ic/Ia) signal output of the immunosensor. Fig. S17. The comparison of Au/rGO-2 with traditional Ru(bpy)32+’s cathodic co-reactant GSH, K2S2O8, and H2O2. Scheme S1. The schematic illustration for cathodic and anodic ECL reaction pathways. Table S1. Comparison of the different potential-resolved platforms for ratiometric ECL immunoassay. Table S2. The XPS atomic of C1s, N1s, O1s, and Au4f on Au/rGO synthesized at different concentrations of HAuCl4 [file research.0117.f1.zip › Figure S6.pdf]

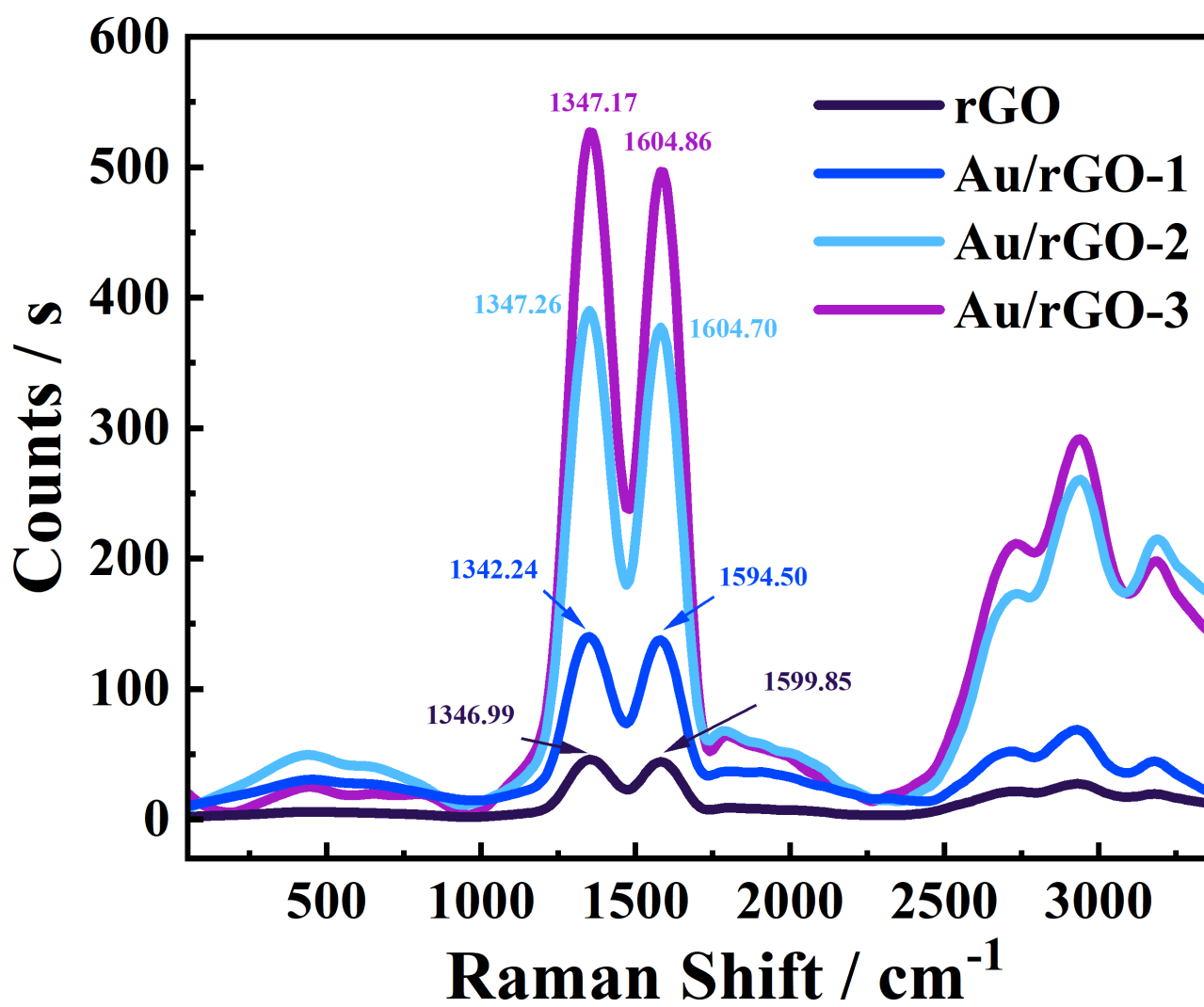

Supplement: Supplementary 1 — Fig. S1. (A) TEM image, (B) HAADF image, and (C) SEM image of Au/rGO. Fig. S2. DPV lines of rGO and Au/rGO on GCE in PBS. Fig. S3. EDS mappings of Au/rGO. Fig. S4. XRD patterns of rGO and Au/rGOs. Fig. S5. Zeta potential of GO, rGO, and Au/rGO. Fig. S6. XPS survey spectra of rGO (A), Au/rGO (B), C1s deconvolution spectrum N1s peak (C), and Au4f region of Au/rGO (D). Fig. S7. Raman spectrum of rGO and Au/rGOs. Fig. S8. RDE lines of Au/rGO-1, Au/rGO-2, and Au/rGO-3 in O2-saturated 0.1 M KOH. Fig. S9. (A) RDE lines in O2-saturated 0.1 M KOH at 1,600 rpm and (B) ECL line in 0.1 M Ru(bpy)32+ of Au/rGO, Ag/rGO, and Pt/rGO. Fig. S10. ECL performance of Au/rGO-2 and Au/rGO-3 under O2, air, and N2 atmospheres. Fig. S11. ECL responses of Au/rGO-2 and Au/rGO-3 in Ru(bpy)32+ with ROS inhibitor BQ, SOD, and isopropanol. Fig. S12. ECL curves of Au/rGO-3/GCE in Ru(bpy)32+ before and after it was electrochemically reduced. Fig. S13. Oxygen and carbon atoms ratio of (A) Au/rGO-2 and (B) Au/rGO-3 before and after the reaction with Ru(bpy)32+. Fig. S14. ECL performance of AuNPs with different diameters (A), GO and rGO (B), and Au/GO-1 and Au/GO-2 as well as Au/rGO-1 and Au/rGO-2 (C) in Ru(bpy)32+. Fig. S15. Ultraviolet-visible absorption spectra of Au/rGO with different GO reduction degrees (A). The logarithm of the anodic to cathodic ECL luminescence intensity of Ru(bpy)32+ reacting with Au/rGO with different rGO reduction times (B). Fig. S16. The effect of (A) pH, (B) C[Au/rGO-2]/C[Au/rGO-3], and (C) Ru(bpy)32+’s concentration on lg(Ic/Ia) signal output of the immunosensor. Fig. S17. The comparison of Au/rGO-2 with traditional Ru(bpy)32+’s cathodic co-reactant GSH, K2S2O8, and H2O2. Scheme S1. The schematic illustration for cathodic and anodic ECL reaction pathways. Table S1. Comparison of the different potential-resolved platforms for ratiometric ECL immunoassay. Table S2. The XPS atomic of C1s, N1s, O1s, and Au4f on Au/rGO synthesized at different concentrations of HAuCl4 [file research.0117.f1.zip › Figure S7.pdf]

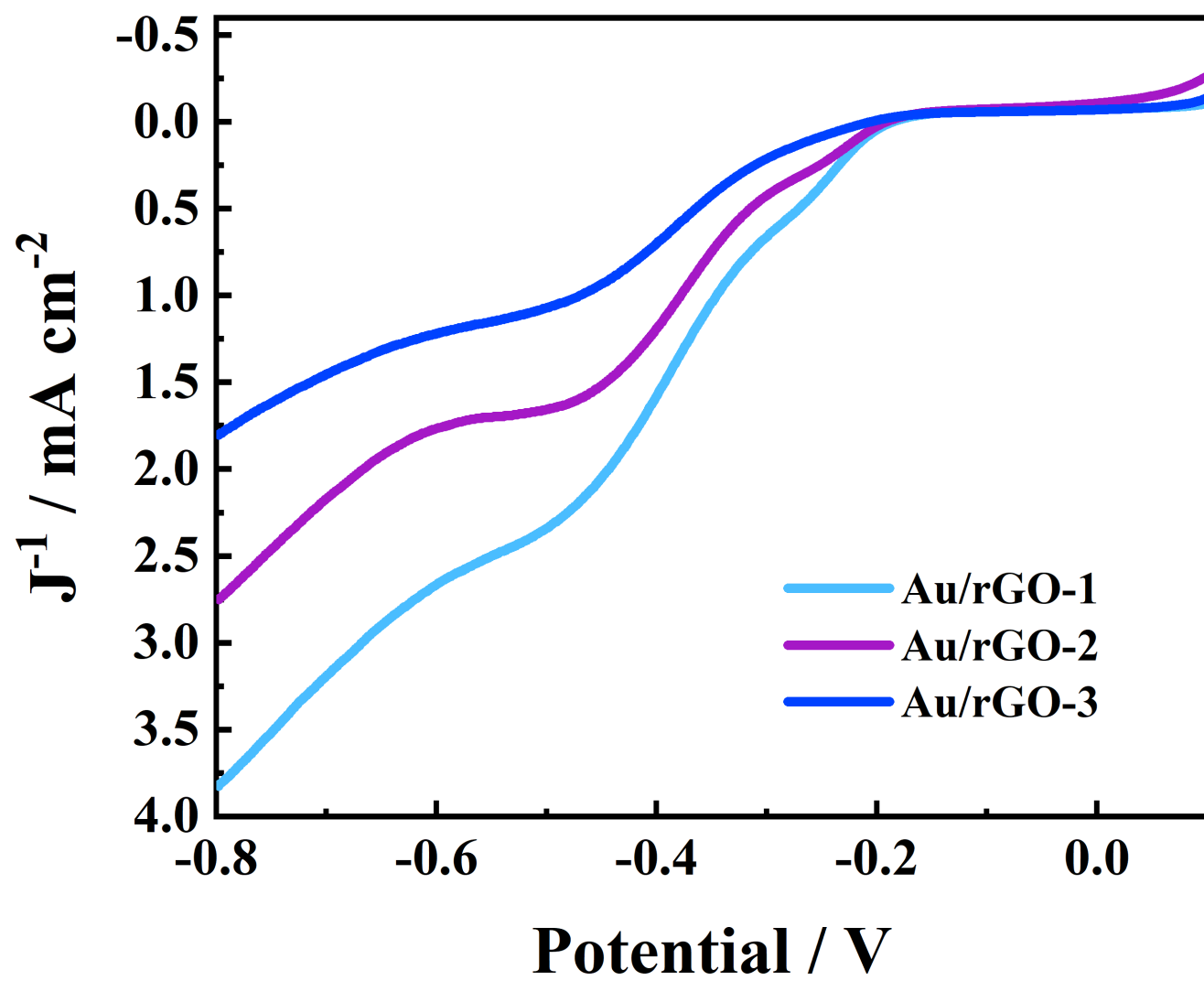

Supplement: Supplementary 1 — Fig. S1. (A) TEM image, (B) HAADF image, and (C) SEM image of Au/rGO. Fig. S2. DPV lines of rGO and Au/rGO on GCE in PBS. Fig. S3. EDS mappings of Au/rGO. Fig. S4. XRD patterns of rGO and Au/rGOs. Fig. S5. Zeta potential of GO, rGO, and Au/rGO. Fig. S6. XPS survey spectra of rGO (A), Au/rGO (B), C1s deconvolution spectrum N1s peak (C), and Au4f region of Au/rGO (D). Fig. S7. Raman spectrum of rGO and Au/rGOs. Fig. S8. RDE lines of Au/rGO-1, Au/rGO-2, and Au/rGO-3 in O2-saturated 0.1 M KOH. Fig. S9. (A) RDE lines in O2-saturated 0.1 M KOH at 1,600 rpm and (B) ECL line in 0.1 M Ru(bpy)32+ of Au/rGO, Ag/rGO, and Pt/rGO. Fig. S10. ECL performance of Au/rGO-2 and Au/rGO-3 under O2, air, and N2 atmospheres. Fig. S11. ECL responses of Au/rGO-2 and Au/rGO-3 in Ru(bpy)32+ with ROS inhibitor BQ, SOD, and isopropanol. Fig. S12. ECL curves of Au/rGO-3/GCE in Ru(bpy)32+ before and after it was electrochemically reduced. Fig. S13. Oxygen and carbon atoms ratio of (A) Au/rGO-2 and (B) Au/rGO-3 before and after the reaction with Ru(bpy)32+. Fig. S14. ECL performance of AuNPs with different diameters (A), GO and rGO (B), and Au/GO-1 and Au/GO-2 as well as Au/rGO-1 and Au/rGO-2 (C) in Ru(bpy)32+. Fig. S15. Ultraviolet-visible absorption spectra of Au/rGO with different GO reduction degrees (A). The logarithm of the anodic to cathodic ECL luminescence intensity of Ru(bpy)32+ reacting with Au/rGO with different rGO reduction times (B). Fig. S16. The effect of (A) pH, (B) C[Au/rGO-2]/C[Au/rGO-3], and (C) Ru(bpy)32+’s concentration on lg(Ic/Ia) signal output of the immunosensor. Fig. S17. The comparison of Au/rGO-2 with traditional Ru(bpy)32+’s cathodic co-reactant GSH, K2S2O8, and H2O2. Scheme S1. The schematic illustration for cathodic and anodic ECL reaction pathways. Table S1. Comparison of the different potential-resolved platforms for ratiometric ECL immunoassay. Table S2. The XPS atomic of C1s, N1s, O1s, and Au4f on Au/rGO synthesized at different concentrations of HAuCl4 [file research.0117.f1.zip › Figure S8.pdf]

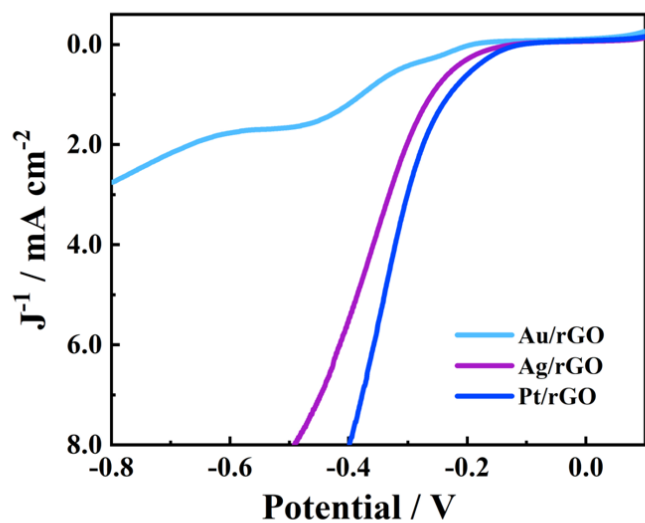

(a)

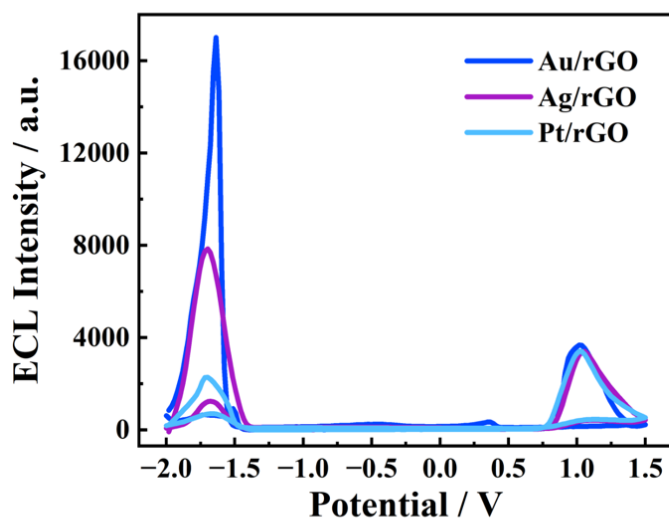

(b)

Supplement: Supplementary 1 — Fig. S1. (A) TEM image, (B) HAADF image, and (C) SEM image of Au/rGO. Fig. S2. DPV lines of rGO and Au/rGO on GCE in PBS. Fig. S3. EDS mappings of Au/rGO. Fig. S4. XRD patterns of rGO and Au/rGOs. Fig. S5. Zeta potential of GO, rGO, and Au/rGO. Fig. S6. XPS survey spectra of rGO (A), Au/rGO (B), C1s deconvolution spectrum N1s peak (C), and Au4f region of Au/rGO (D). Fig. S7. Raman spectrum of rGO and Au/rGOs. Fig. S8. RDE lines of Au/rGO-1, Au/rGO-2, and Au/rGO-3 in O2-saturated 0.1 M KOH. Fig. S9. (A) RDE lines in O2-saturated 0.1 M KOH at 1,600 rpm and (B) ECL line in 0.1 M Ru(bpy)32+ of Au/rGO, Ag/rGO, and Pt/rGO. Fig. S10. ECL performance of Au/rGO-2 and Au/rGO-3 under O2, air, and N2 atmospheres. Fig. S11. ECL responses of Au/rGO-2 and Au/rGO-3 in Ru(bpy)32+ with ROS inhibitor BQ, SOD, and isopropanol. Fig. S12. ECL curves of Au/rGO-3/GCE in Ru(bpy)32+ before and after it was electrochemically reduced. Fig. S13. Oxygen and carbon atoms ratio of (A) Au/rGO-2 and (B) Au/rGO-3 before and after the reaction with Ru(bpy)32+. Fig. S14. ECL performance of AuNPs with different diameters (A), GO and rGO (B), and Au/GO-1 and Au/GO-2 as well as Au/rGO-1 and Au/rGO-2 (C) in Ru(bpy)32+. Fig. S15. Ultraviolet-visible absorption spectra of Au/rGO with different GO reduction degrees (A). The logarithm of the anodic to cathodic ECL luminescence intensity of Ru(bpy)32+ reacting with Au/rGO with different rGO reduction times (B). Fig. S16. The effect of (A) pH, (B) C[Au/rGO-2]/C[Au/rGO-3], and (C) Ru(bpy)32+’s concentration on lg(Ic/Ia) signal output of the immunosensor. Fig. S17. The comparison of Au/rGO-2 with traditional Ru(bpy)32+’s cathodic co-reactant GSH, K2S2O8, and H2O2. Scheme S1. The schematic illustration for cathodic and anodic ECL reaction pathways. Table S1. Comparison of the different potential-resolved platforms for ratiometric ECL immunoassay. Table S2. The XPS atomic of C1s, N1s, O1s, and Au4f on Au/rGO synthesized at different concentrations of HAuCl4 [file research.0117.f1.zip › Figure S9.pdf]

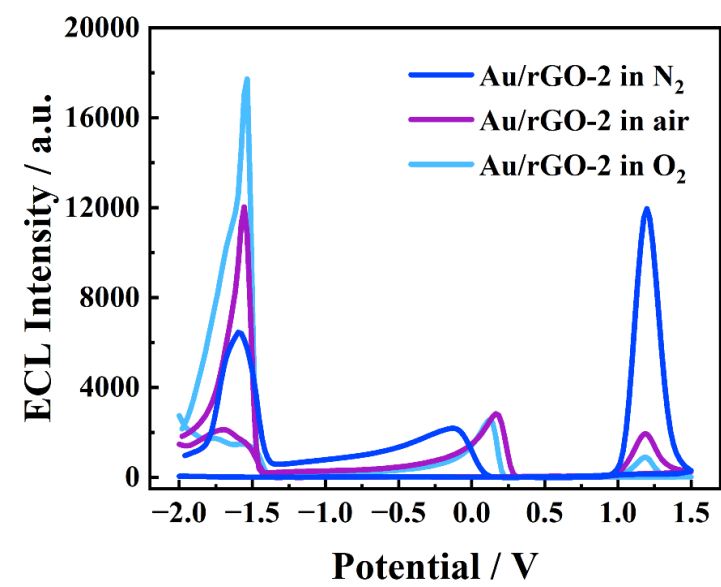

(a)

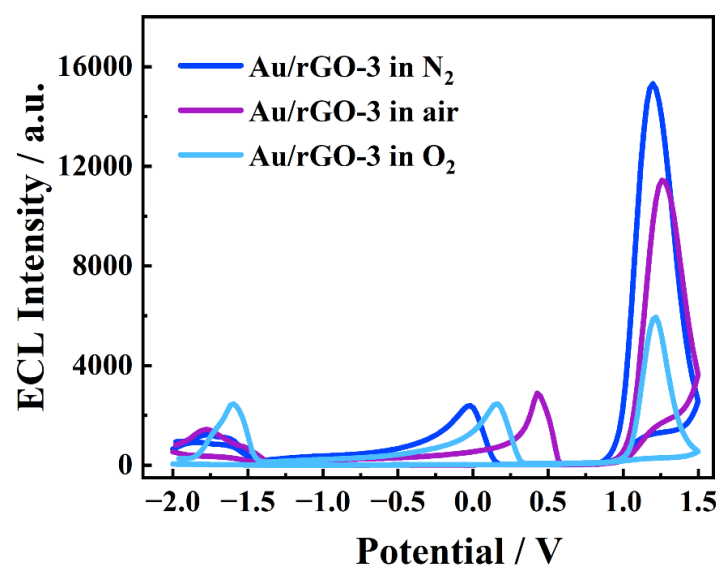

(b)

Supplement: Supplementary 1 — Fig. S1. (A) TEM image, (B) HAADF image, and (C) SEM image of Au/rGO. Fig. S2. DPV lines of rGO and Au/rGO on GCE in PBS. Fig. S3. EDS mappings of Au/rGO. Fig. S4. XRD patterns of rGO and Au/rGOs. Fig. S5. Zeta potential of GO, rGO, and Au/rGO. Fig. S6. XPS survey spectra of rGO (A), Au/rGO (B), C1s deconvolution spectrum N1s peak (C), and Au4f region of Au/rGO (D). Fig. S7. Raman spectrum of rGO and Au/rGOs. Fig. S8. RDE lines of Au/rGO-1, Au/rGO-2, and Au/rGO-3 in O2-saturated 0.1 M KOH. Fig. S9. (A) RDE lines in O2-saturated 0.1 M KOH at 1,600 rpm and (B) ECL line in 0.1 M Ru(bpy)32+ of Au/rGO, Ag/rGO, and Pt/rGO. Fig. S10. ECL performance of Au/rGO-2 and Au/rGO-3 under O2, air, and N2 atmospheres. Fig. S11. ECL responses of Au/rGO-2 and Au/rGO-3 in Ru(bpy)32+ with ROS inhibitor BQ, SOD, and isopropanol. Fig. S12. ECL curves of Au/rGO-3/GCE in Ru(bpy)32+ before and after it was electrochemically reduced. Fig. S13. Oxygen and carbon atoms ratio of (A) Au/rGO-2 and (B) Au/rGO-3 before and after the reaction with Ru(bpy)32+. Fig. S14. ECL performance of AuNPs with different diameters (A), GO and rGO (B), and Au/GO-1 and Au/GO-2 as well as Au/rGO-1 and Au/rGO-2 (C) in Ru(bpy)32+. Fig. S15. Ultraviolet-visible absorption spectra of Au/rGO with different GO reduction degrees (A). The logarithm of the anodic to cathodic ECL luminescence intensity of Ru(bpy)32+ reacting with Au/rGO with different rGO reduction times (B). Fig. S16. The effect of (A) pH, (B) C[Au/rGO-2]/C[Au/rGO-3], and (C) Ru(bpy)32+’s concentration on lg(Ic/Ia) signal output of the immunosensor. Fig. S17. The comparison of Au/rGO-2 with traditional Ru(bpy)32+’s cathodic co-reactant GSH, K2S2O8, and H2O2. Scheme S1. The schematic illustration for cathodic and anodic ECL reaction pathways. Table S1. Comparison of the different potential-resolved platforms for ratiometric ECL immunoassay. Table S2. The XPS atomic of C1s, N1s, O1s, and Au4f on Au/rGO synthesized at different concentrations of HAuCl4 [file research.0117.f1.zip › Figure S10.pdf]

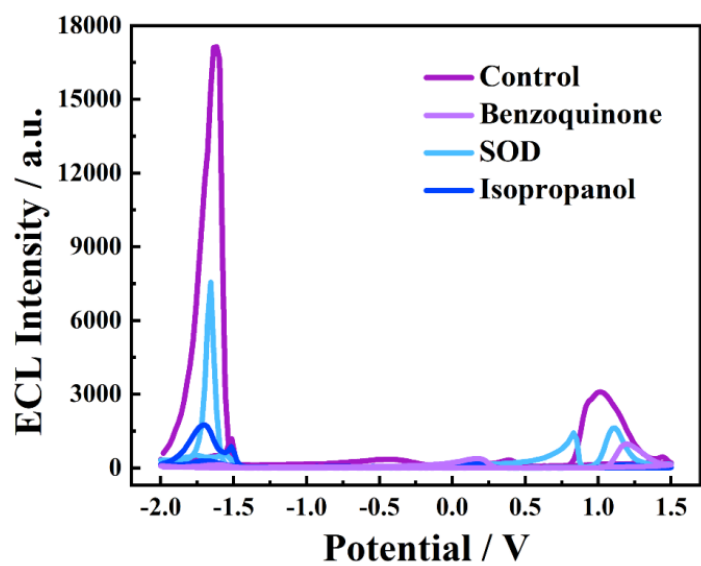

(a)

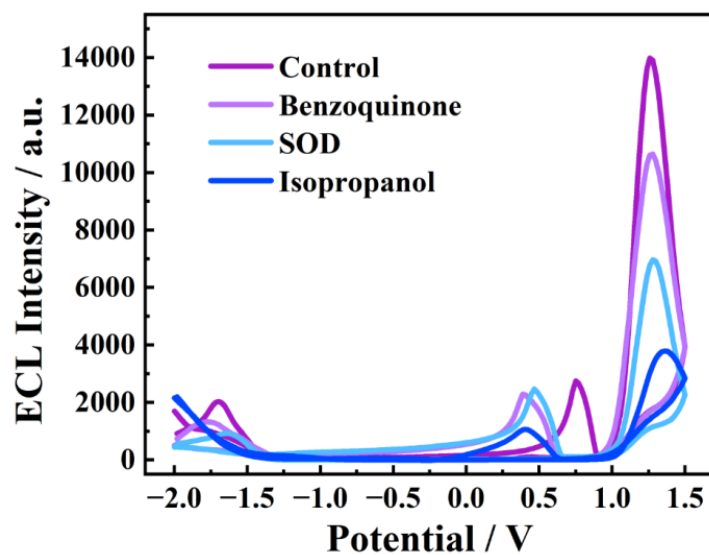

(b)

Supplement: Supplementary 1 — Fig. S1. (A) TEM image, (B) HAADF image, and (C) SEM image of Au/rGO. Fig. S2. DPV lines of rGO and Au/rGO on GCE in PBS. Fig. S3. EDS mappings of Au/rGO. Fig. S4. XRD patterns of rGO and Au/rGOs. Fig. S5. Zeta potential of GO, rGO, and Au/rGO. Fig. S6. XPS survey spectra of rGO (A), Au/rGO (B), C1s deconvolution spectrum N1s peak (C), and Au4f region of Au/rGO (D). Fig. S7. Raman spectrum of rGO and Au/rGOs. Fig. S8. RDE lines of Au/rGO-1, Au/rGO-2, and Au/rGO-3 in O2-saturated 0.1 M KOH. Fig. S9. (A) RDE lines in O2-saturated 0.1 M KOH at 1,600 rpm and (B) ECL line in 0.1 M Ru(bpy)32+ of Au/rGO, Ag/rGO, and Pt/rGO. Fig. S10. ECL performance of Au/rGO-2 and Au/rGO-3 under O2, air, and N2 atmospheres. Fig. S11. ECL responses of Au/rGO-2 and Au/rGO-3 in Ru(bpy)32+ with ROS inhibitor BQ, SOD, and isopropanol. Fig. S12. ECL curves of Au/rGO-3/GCE in Ru(bpy)32+ before and after it was electrochemically reduced. Fig. S13. Oxygen and carbon atoms ratio of (A) Au/rGO-2 and (B) Au/rGO-3 before and after the reaction with Ru(bpy)32+. Fig. S14. ECL performance of AuNPs with different diameters (A), GO and rGO (B), and Au/GO-1 and Au/GO-2 as well as Au/rGO-1 and Au/rGO-2 (C) in Ru(bpy)32+. Fig. S15. Ultraviolet-visible absorption spectra of Au/rGO with different GO reduction degrees (A). The logarithm of the anodic to cathodic ECL luminescence intensity of Ru(bpy)32+ reacting with Au/rGO with different rGO reduction times (B). Fig. S16. The effect of (A) pH, (B) C[Au/rGO-2]/C[Au/rGO-3], and (C) Ru(bpy)32+’s concentration on lg(Ic/Ia) signal output of the immunosensor. Fig. S17. The comparison of Au/rGO-2 with traditional Ru(bpy)32+’s cathodic co-reactant GSH, K2S2O8, and H2O2. Scheme S1. The schematic illustration for cathodic and anodic ECL reaction pathways. Table S1. Comparison of the different potential-resolved platforms for ratiometric ECL immunoassay. Table S2. The XPS atomic of C1s, N1s, O1s, and Au4f on Au/rGO synthesized at different concentrations of HAuCl4 [file research.0117.f1.zip › Figure S11.pdf]

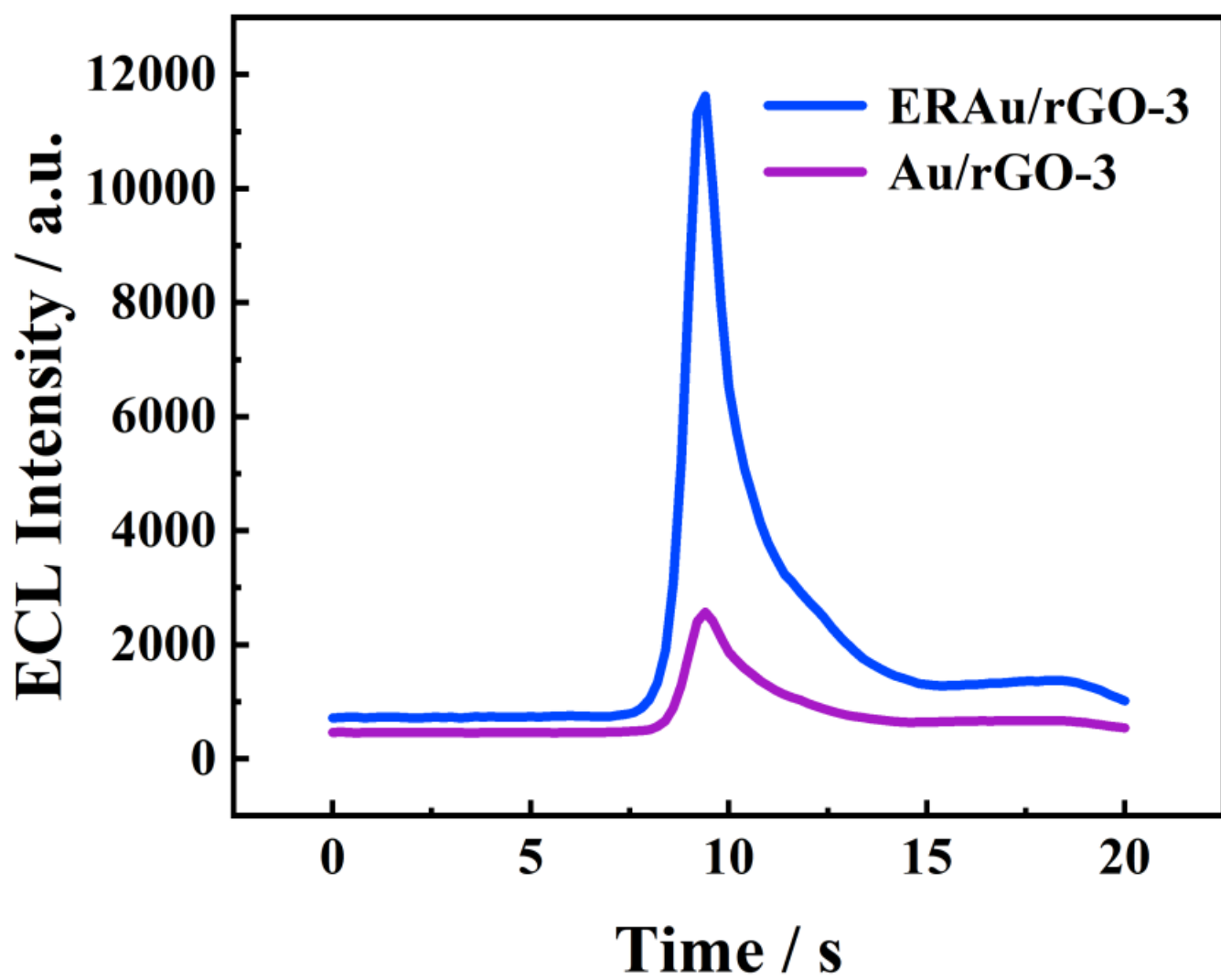

Supplement: Supplementary 1 — Fig. S1. (A) TEM image, (B) HAADF image, and (C) SEM image of Au/rGO. Fig. S2. DPV lines of rGO and Au/rGO on GCE in PBS. Fig. S3. EDS mappings of Au/rGO. Fig. S4. XRD patterns of rGO and Au/rGOs. Fig. S5. Zeta potential of GO, rGO, and Au/rGO. Fig. S6. XPS survey spectra of rGO (A), Au/rGO (B), C1s deconvolution spectrum N1s peak (C), and Au4f region of Au/rGO (D). Fig. S7. Raman spectrum of rGO and Au/rGOs. Fig. S8. RDE lines of Au/rGO-1, Au/rGO-2, and Au/rGO-3 in O2-saturated 0.1 M KOH. Fig. S9. (A) RDE lines in O2-saturated 0.1 M KOH at 1,600 rpm and (B) ECL line in 0.1 M Ru(bpy)32+ of Au/rGO, Ag/rGO, and Pt/rGO. Fig. S10. ECL performance of Au/rGO-2 and Au/rGO-3 under O2, air, and N2 atmospheres. Fig. S11. ECL responses of Au/rGO-2 and Au/rGO-3 in Ru(bpy)32+ with ROS inhibitor BQ, SOD, and isopropanol. Fig. S12. ECL curves of Au/rGO-3/GCE in Ru(bpy)32+ before and after it was electrochemically reduced. Fig. S13. Oxygen and carbon atoms ratio of (A) Au/rGO-2 and (B) Au/rGO-3 before and after the reaction with Ru(bpy)32+. Fig. S14. ECL performance of AuNPs with different diameters (A), GO and rGO (B), and Au/GO-1 and Au/GO-2 as well as Au/rGO-1 and Au/rGO-2 (C) in Ru(bpy)32+. Fig. S15. Ultraviolet-visible absorption spectra of Au/rGO with different GO reduction degrees (A). The logarithm of the anodic to cathodic ECL luminescence intensity of Ru(bpy)32+ reacting with Au/rGO with different rGO reduction times (B). Fig. S16. The effect of (A) pH, (B) C[Au/rGO-2]/C[Au/rGO-3], and (C) Ru(bpy)32+’s concentration on lg(Ic/Ia) signal output of the immunosensor. Fig. S17. The comparison of Au/rGO-2 with traditional Ru(bpy)32+’s cathodic co-reactant GSH, K2S2O8, and H2O2. Scheme S1. The schematic illustration for cathodic and anodic ECL reaction pathways. Table S1. Comparison of the different potential-resolved platforms for ratiometric ECL immunoassay. Table S2. The XPS atomic of C1s, N1s, O1s, and Au4f on Au/rGO synthesized at different concentrations of HAuCl4 [file research.0117.f1.zip › Figure S12.pdf]

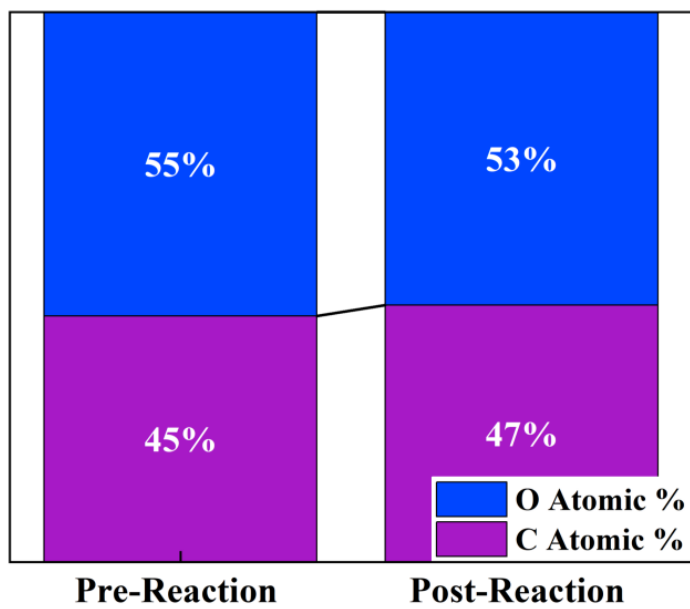

(a)

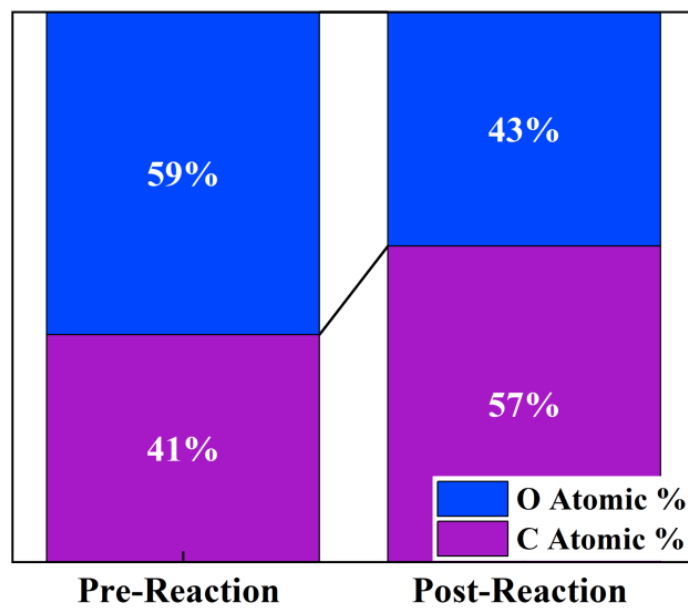

(b)

Supplement: Supplementary 1 — Fig. S1. (A) TEM image, (B) HAADF image, and (C) SEM image of Au/rGO. Fig. S2. DPV lines of rGO and Au/rGO on GCE in PBS. Fig. S3. EDS mappings of Au/rGO. Fig. S4. XRD patterns of rGO and Au/rGOs. Fig. S5. Zeta potential of GO, rGO, and Au/rGO. Fig. S6. XPS survey spectra of rGO (A), Au/rGO (B), C1s deconvolution spectrum N1s peak (C), and Au4f region of Au/rGO (D). Fig. S7. Raman spectrum of rGO and Au/rGOs. Fig. S8. RDE lines of Au/rGO-1, Au/rGO-2, and Au/rGO-3 in O2-saturated 0.1 M KOH. Fig. S9. (A) RDE lines in O2-saturated 0.1 M KOH at 1,600 rpm and (B) ECL line in 0.1 M Ru(bpy)32+ of Au/rGO, Ag/rGO, and Pt/rGO. Fig. S10. ECL performance of Au/rGO-2 and Au/rGO-3 under O2, air, and N2 atmospheres. Fig. S11. ECL responses of Au/rGO-2 and Au/rGO-3 in Ru(bpy)32+ with ROS inhibitor BQ, SOD, and isopropanol. Fig. S12. ECL curves of Au/rGO-3/GCE in Ru(bpy)32+ before and after it was electrochemically reduced. Fig. S13. Oxygen and carbon atoms ratio of (A) Au/rGO-2 and (B) Au/rGO-3 before and after the reaction with Ru(bpy)32+. Fig. S14. ECL performance of AuNPs with different diameters (A), GO and rGO (B), and Au/GO-1 and Au/GO-2 as well as Au/rGO-1 and Au/rGO-2 (C) in Ru(bpy)32+. Fig. S15. Ultraviolet-visible absorption spectra of Au/rGO with different GO reduction degrees (A). The logarithm of the anodic to cathodic ECL luminescence intensity of Ru(bpy)32+ reacting with Au/rGO with different rGO reduction times (B). Fig. S16. The effect of (A) pH, (B) C[Au/rGO-2]/C[Au/rGO-3], and (C) Ru(bpy)32+’s concentration on lg(Ic/Ia) signal output of the immunosensor. Fig. S17. The comparison of Au/rGO-2 with traditional Ru(bpy)32+’s cathodic co-reactant GSH, K2S2O8, and H2O2. Scheme S1. The schematic illustration for cathodic and anodic ECL reaction pathways. Table S1. Comparison of the different potential-resolved platforms for ratiometric ECL immunoassay. Table S2. The XPS atomic of C1s, N1s, O1s, and Au4f on Au/rGO synthesized at different concentrations of HAuCl4 [file research.0117.f1.zip › Figure S13.pdf]

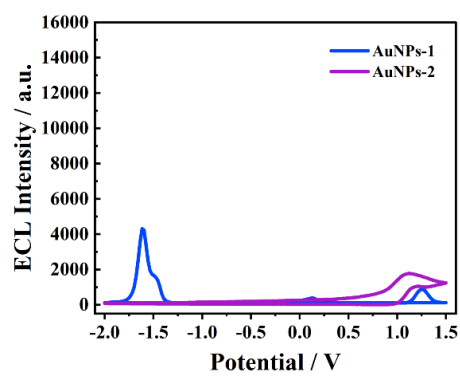

(a)

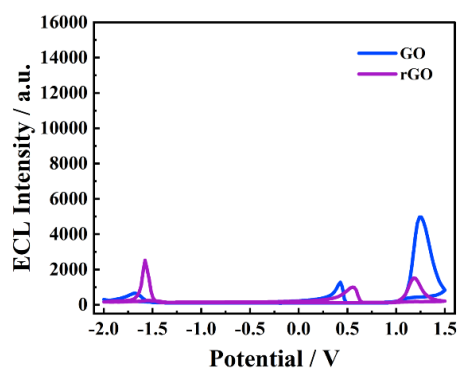

(b)

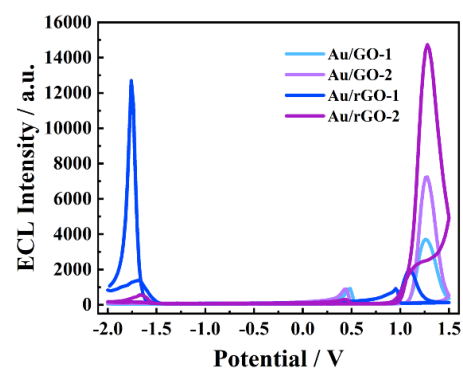

(c)

Supplement: Supplementary 1 — Fig. S1. (A) TEM image, (B) HAADF image, and (C) SEM image of Au/rGO. Fig. S2. DPV lines of rGO and Au/rGO on GCE in PBS. Fig. S3. EDS mappings of Au/rGO. Fig. S4. XRD patterns of rGO and Au/rGOs. Fig. S5. Zeta potential of GO, rGO, and Au/rGO. Fig. S6. XPS survey spectra of rGO (A), Au/rGO (B), C1s deconvolution spectrum N1s peak (C), and Au4f region of Au/rGO (D). Fig. S7. Raman spectrum of rGO and Au/rGOs. Fig. S8. RDE lines of Au/rGO-1, Au/rGO-2, and Au/rGO-3 in O2-saturated 0.1 M KOH. Fig. S9. (A) RDE lines in O2-saturated 0.1 M KOH at 1,600 rpm and (B) ECL line in 0.1 M Ru(bpy)32+ of Au/rGO, Ag/rGO, and Pt/rGO. Fig. S10. ECL performance of Au/rGO-2 and Au/rGO-3 under O2, air, and N2 atmospheres. Fig. S11. ECL responses of Au/rGO-2 and Au/rGO-3 in Ru(bpy)32+ with ROS inhibitor BQ, SOD, and isopropanol. Fig. S12. ECL curves of Au/rGO-3/GCE in Ru(bpy)32+ before and after it was electrochemically reduced. Fig. S13. Oxygen and carbon atoms ratio of (A) Au/rGO-2 and (B) Au/rGO-3 before and after the reaction with Ru(bpy)32+. Fig. S14. ECL performance of AuNPs with different diameters (A), GO and rGO (B), and Au/GO-1 and Au/GO-2 as well as Au/rGO-1 and Au/rGO-2 (C) in Ru(bpy)32+. Fig. S15. Ultraviolet-visible absorption spectra of Au/rGO with different GO reduction degrees (A). The logarithm of the anodic to cathodic ECL luminescence intensity of Ru(bpy)32+ reacting with Au/rGO with different rGO reduction times (B). Fig. S16. The effect of (A) pH, (B) C[Au/rGO-2]/C[Au/rGO-3], and (C) Ru(bpy)32+’s concentration on lg(Ic/Ia) signal output of the immunosensor. Fig. S17. The comparison of Au/rGO-2 with traditional Ru(bpy)32+’s cathodic co-reactant GSH, K2S2O8, and H2O2. Scheme S1. The schematic illustration for cathodic and anodic ECL reaction pathways. Table S1. Comparison of the different potential-resolved platforms for ratiometric ECL immunoassay. Table S2. The XPS atomic of C1s, N1s, O1s, and Au4f on Au/rGO synthesized at different concentrations of HAuCl4 [file research.0117.f1.zip › Figure S14.pdf]

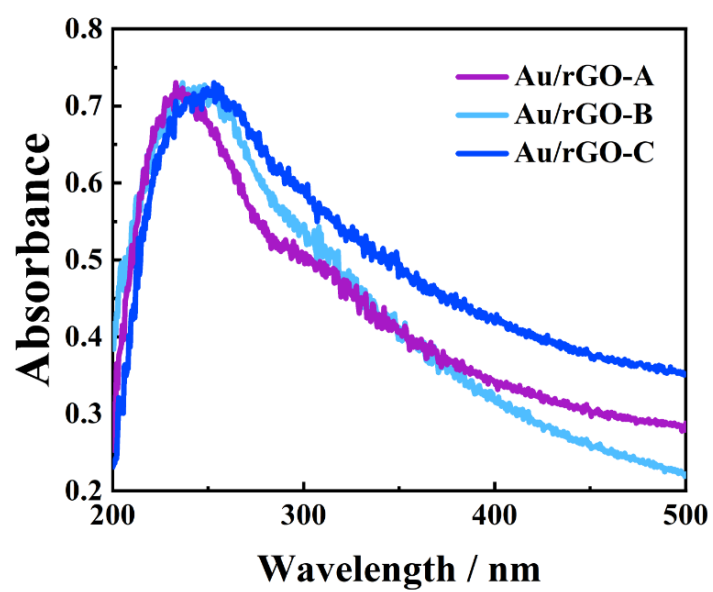

(a)

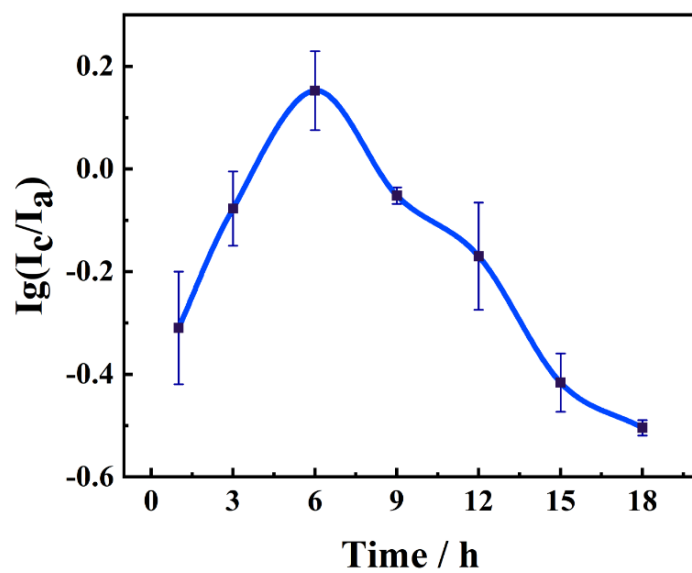

(b)

Supplement: Supplementary 1 — Fig. S1. (A) TEM image, (B) HAADF image, and (C) SEM image of Au/rGO. Fig. S2. DPV lines of rGO and Au/rGO on GCE in PBS. Fig. S3. EDS mappings of Au/rGO. Fig. S4. XRD patterns of rGO and Au/rGOs. Fig. S5. Zeta potential of GO, rGO, and Au/rGO. Fig. S6. XPS survey spectra of rGO (A), Au/rGO (B), C1s deconvolution spectrum N1s peak (C), and Au4f region of Au/rGO (D). Fig. S7. Raman spectrum of rGO and Au/rGOs. Fig. S8. RDE lines of Au/rGO-1, Au/rGO-2, and Au/rGO-3 in O2-saturated 0.1 M KOH. Fig. S9. (A) RDE lines in O2-saturated 0.1 M KOH at 1,600 rpm and (B) ECL line in 0.1 M Ru(bpy)32+ of Au/rGO, Ag/rGO, and Pt/rGO. Fig. S10. ECL performance of Au/rGO-2 and Au/rGO-3 under O2, air, and N2 atmospheres. Fig. S11. ECL responses of Au/rGO-2 and Au/rGO-3 in Ru(bpy)32+ with ROS inhibitor BQ, SOD, and isopropanol. Fig. S12. ECL curves of Au/rGO-3/GCE in Ru(bpy)32+ before and after it was electrochemically reduced. Fig. S13. Oxygen and carbon atoms ratio of (A) Au/rGO-2 and (B) Au/rGO-3 before and after the reaction with Ru(bpy)32+. Fig. S14. ECL performance of AuNPs with different diameters (A), GO and rGO (B), and Au/GO-1 and Au/GO-2 as well as Au/rGO-1 and Au/rGO-2 (C) in Ru(bpy)32+. Fig. S15. Ultraviolet-visible absorption spectra of Au/rGO with different GO reduction degrees (A). The logarithm of the anodic to cathodic ECL luminescence intensity of Ru(bpy)32+ reacting with Au/rGO with different rGO reduction times (B). Fig. S16. The effect of (A) pH, (B) C[Au/rGO-2]/C[Au/rGO-3], and (C) Ru(bpy)32+’s concentration on lg(Ic/Ia) signal output of the immunosensor. Fig. S17. The comparison of Au/rGO-2 with traditional Ru(bpy)32+’s cathodic co-reactant GSH, K2S2O8, and H2O2. Scheme S1. The schematic illustration for cathodic and anodic ECL reaction pathways. Table S1. Comparison of the different potential-resolved platforms for ratiometric ECL immunoassay. Table S2. The XPS atomic of C1s, N1s, O1s, and Au4f on Au/rGO synthesized at different concentrations of HAuCl4 [file research.0117.f1.zip › Figure S15.pdf]

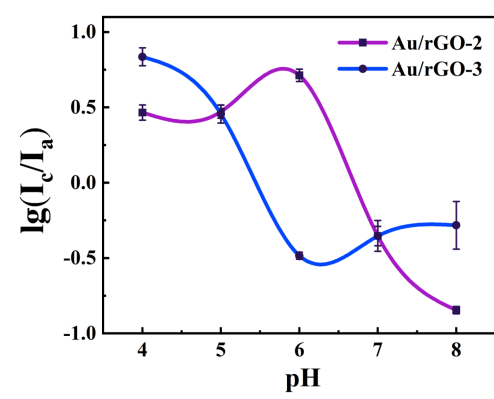

(e)

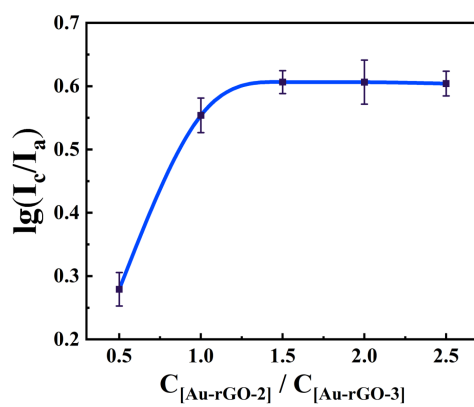

(f)

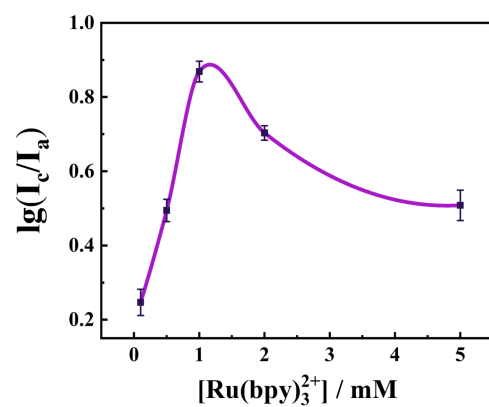

(g)

Supplement: Supplementary 1 — Fig. S1. (A) TEM image, (B) HAADF image, and (C) SEM image of Au/rGO. Fig. S2. DPV lines of rGO and Au/rGO on GCE in PBS. Fig. S3. EDS mappings of Au/rGO. Fig. S4. XRD patterns of rGO and Au/rGOs. Fig. S5. Zeta potential of GO, rGO, and Au/rGO. Fig. S6. XPS survey spectra of rGO (A), Au/rGO (B), C1s deconvolution spectrum N1s peak (C), and Au4f region of Au/rGO (D). Fig. S7. Raman spectrum of rGO and Au/rGOs. Fig. S8. RDE lines of Au/rGO-1, Au/rGO-2, and Au/rGO-3 in O2-saturated 0.1 M KOH. Fig. S9. (A) RDE lines in O2-saturated 0.1 M KOH at 1,600 rpm and (B) ECL line in 0.1 M Ru(bpy)32+ of Au/rGO, Ag/rGO, and Pt/rGO. Fig. S10. ECL performance of Au/rGO-2 and Au/rGO-3 under O2, air, and N2 atmospheres. Fig. S11. ECL responses of Au/rGO-2 and Au/rGO-3 in Ru(bpy)32+ with ROS inhibitor BQ, SOD, and isopropanol. Fig. S12. ECL curves of Au/rGO-3/GCE in Ru(bpy)32+ before and after it was electrochemically reduced. Fig. S13. Oxygen and carbon atoms ratio of (A) Au/rGO-2 and (B) Au/rGO-3 before and after the reaction with Ru(bpy)32+. Fig. S14. ECL performance of AuNPs with different diameters (A), GO and rGO (B), and Au/GO-1 and Au/GO-2 as well as Au/rGO-1 and Au/rGO-2 (C) in Ru(bpy)32+. Fig. S15. Ultraviolet-visible absorption spectra of Au/rGO with different GO reduction degrees (A). The logarithm of the anodic to cathodic ECL luminescence intensity of Ru(bpy)32+ reacting with Au/rGO with different rGO reduction times (B). Fig. S16. The effect of (A) pH, (B) C[Au/rGO-2]/C[Au/rGO-3], and (C) Ru(bpy)32+’s concentration on lg(Ic/Ia) signal output of the immunosensor. Fig. S17. The comparison of Au/rGO-2 with traditional Ru(bpy)32+’s cathodic co-reactant GSH, K2S2O8, and H2O2. Scheme S1. The schematic illustration for cathodic and anodic ECL reaction pathways. Table S1. Comparison of the different potential-resolved platforms for ratiometric ECL immunoassay. Table S2. The XPS atomic of C1s, N1s, O1s, and Au4f on Au/rGO synthesized at different concentrations of HAuCl4 [file research.0117.f1.zip › Figure S16.pdf]

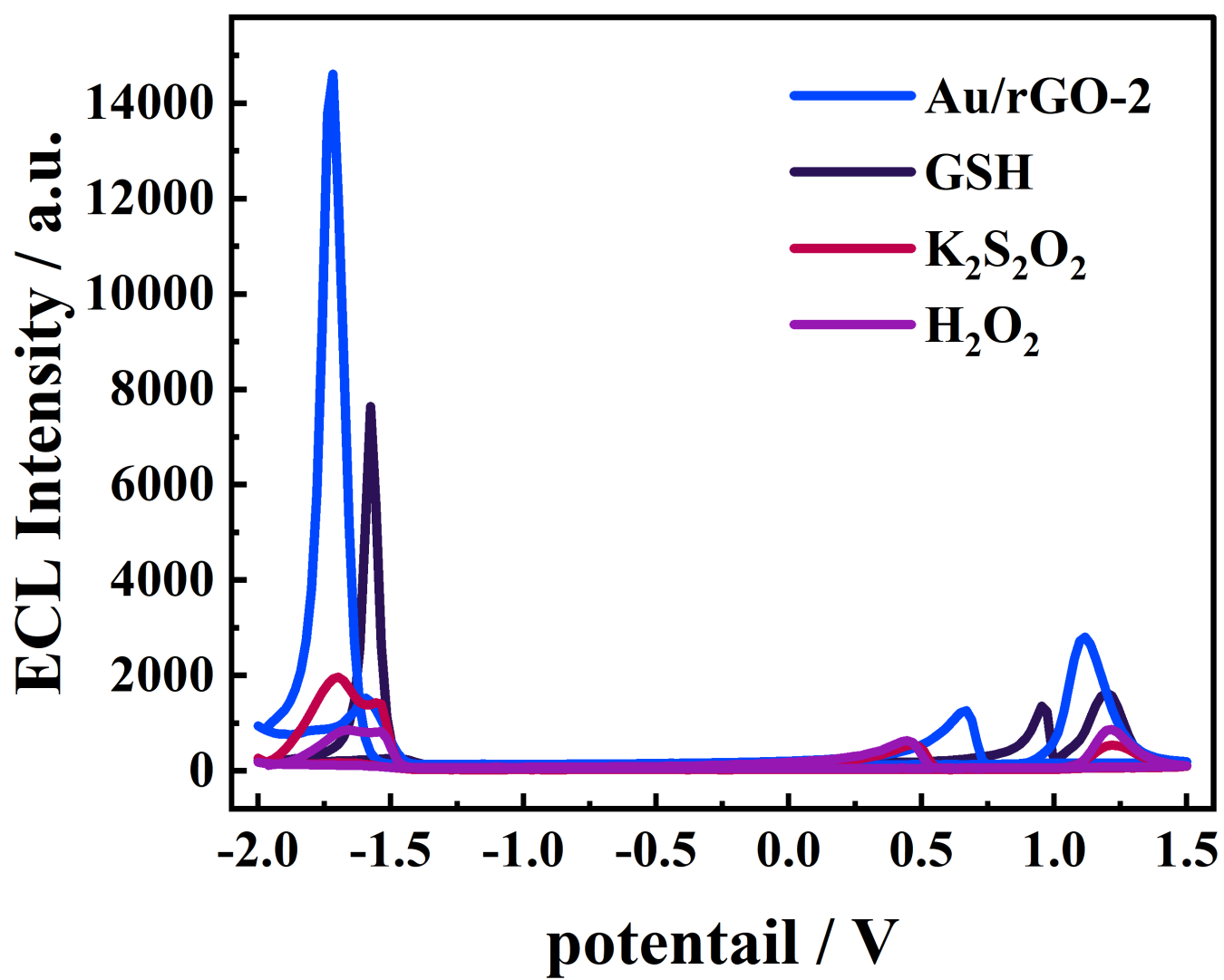

Supplement: Supplementary 1 — Fig. S1. (A) TEM image, (B) HAADF image, and (C) SEM image of Au/rGO. Fig. S2. DPV lines of rGO and Au/rGO on GCE in PBS. Fig. S3. EDS mappings of Au/rGO. Fig. S4. XRD patterns of rGO and Au/rGOs. Fig. S5. Zeta potential of GO, rGO, and Au/rGO. Fig. S6. XPS survey spectra of rGO (A), Au/rGO (B), C1s deconvolution spectrum N1s peak (C), and Au4f region of Au/rGO (D). Fig. S7. Raman spectrum of rGO and Au/rGOs. Fig. S8. RDE lines of Au/rGO-1, Au/rGO-2, and Au/rGO-3 in O2-saturated 0.1 M KOH. Fig. S9. (A) RDE lines in O2-saturated 0.1 M KOH at 1,600 rpm and (B) ECL line in 0.1 M Ru(bpy)32+ of Au/rGO, Ag/rGO, and Pt/rGO. Fig. S10. ECL performance of Au/rGO-2 and Au/rGO-3 under O2, air, and N2 atmospheres. Fig. S11. ECL responses of Au/rGO-2 and Au/rGO-3 in Ru(bpy)32+ with ROS inhibitor BQ, SOD, and isopropanol. Fig. S12. ECL curves of Au/rGO-3/GCE in Ru(bpy)32+ before and after it was electrochemically reduced. Fig. S13. Oxygen and carbon atoms ratio of (A) Au/rGO-2 and (B) Au/rGO-3 before and after the reaction with Ru(bpy)32+. Fig. S14. ECL performance of AuNPs with different diameters (A), GO and rGO (B), and Au/GO-1 and Au/GO-2 as well as Au/rGO-1 and Au/rGO-2 (C) in Ru(bpy)32+. Fig. S15. Ultraviolet-visible absorption spectra of Au/rGO with different GO reduction degrees (A). The logarithm of the anodic to cathodic ECL luminescence intensity of Ru(bpy)32+ reacting with Au/rGO with different rGO reduction times (B). Fig. S16. The effect of (A) pH, (B) C[Au/rGO-2]/C[Au/rGO-3], and (C) Ru(bpy)32+’s concentration on lg(Ic/Ia) signal output of the immunosensor. Fig. S17. The comparison of Au/rGO-2 with traditional Ru(bpy)32+’s cathodic co-reactant GSH, K2S2O8, and H2O2. Scheme S1. The schematic illustration for cathodic and anodic ECL reaction pathways. Table S1. Comparison of the different potential-resolved platforms for ratiometric ECL immunoassay. Table S2. The XPS atomic of C1s, N1s, O1s, and Au4f on Au/rGO synthesized at different concentrations of HAuCl4 [file research.0117.f1.zip › Figure S17.pdf]

# Reaction Mechanism Pathways

## a. Cathodic Reaction

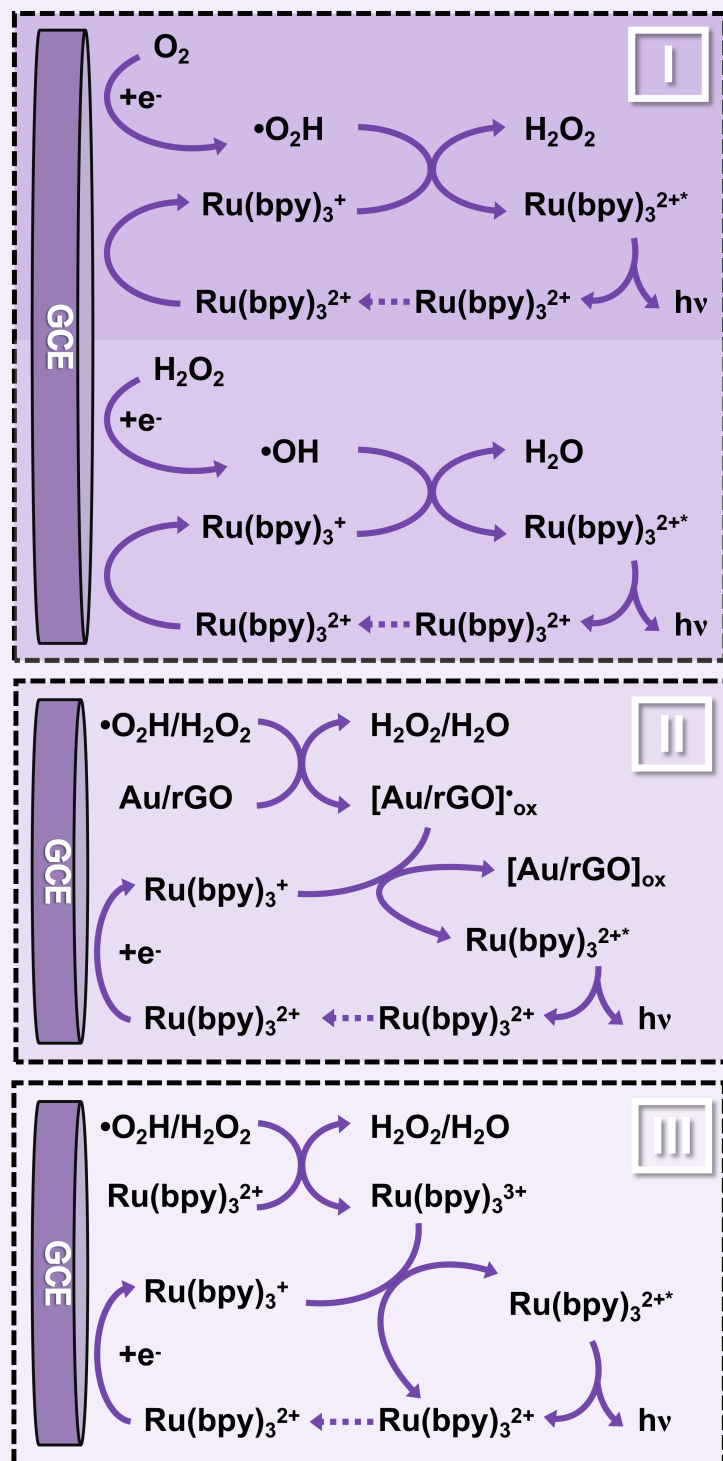

## b. Anodic Reaction

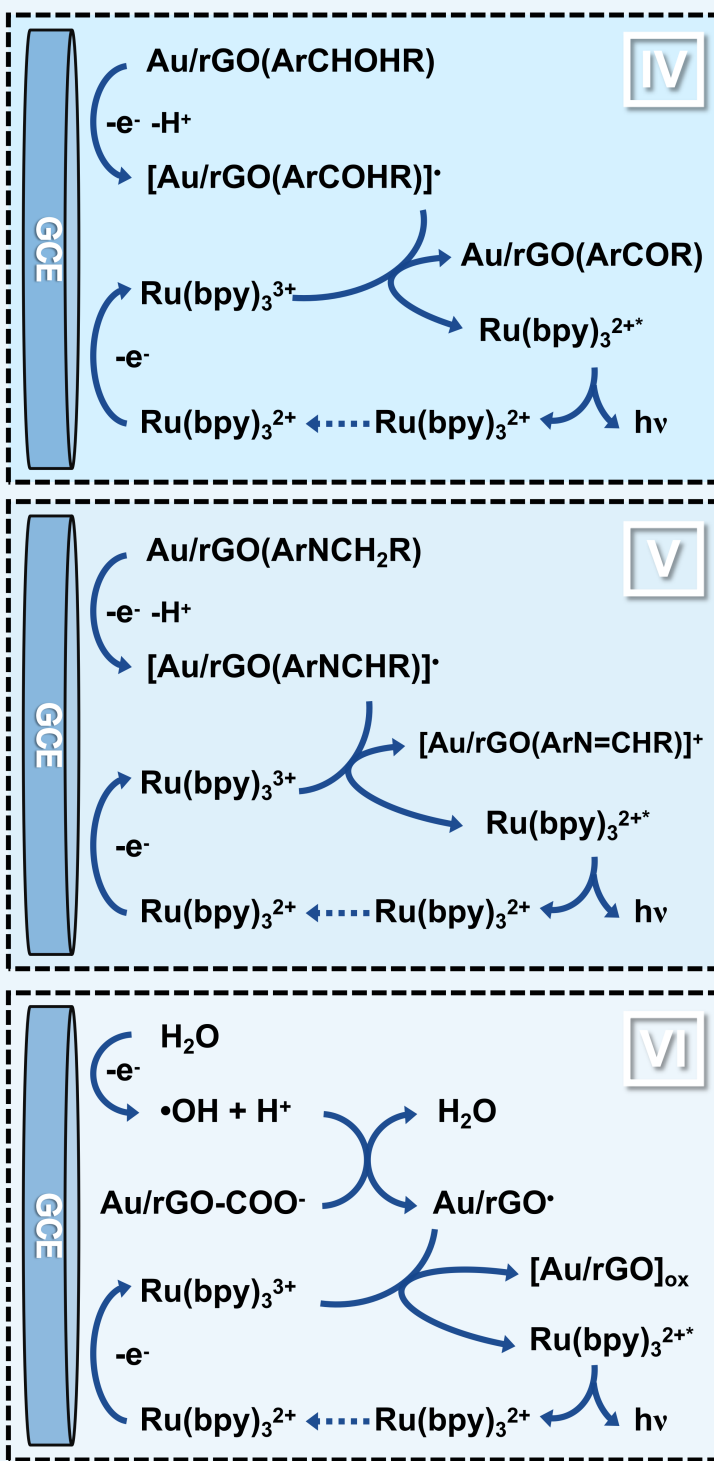

Supplement: Supplementary 1 — Fig. S1. (A) TEM image, (B) HAADF image, and (C) SEM image of Au/rGO. Fig. S2. DPV lines of rGO and Au/rGO on GCE in PBS. Fig. S3. EDS mappings of Au/rGO. Fig. S4. XRD patterns of rGO and Au/rGOs. Fig. S5. Zeta potential of GO, rGO, and Au/rGO. Fig. S6. XPS survey spectra of rGO (A), Au/rGO (B), C1s deconvolution spectrum N1s peak (C), and Au4f region of Au/rGO (D). Fig. S7. Raman spectrum of rGO and Au/rGOs. Fig. S8. RDE lines of Au/rGO-1, Au/rGO-2, and Au/rGO-3 in O2-saturated 0.1 M KOH. Fig. S9. (A) RDE lines in O2-saturated 0.1 M KOH at 1,600 rpm and (B) ECL line in 0.1 M Ru(bpy)32+ of Au/rGO, Ag/rGO, and Pt/rGO. Fig. S10. ECL performance of Au/rGO-2 and Au/rGO-3 under O2, air, and N2 atmospheres. Fig. S11. ECL responses of Au/rGO-2 and Au/rGO-3 in Ru(bpy)32+ with ROS inhibitor BQ, SOD, and isopropanol. Fig. S12. ECL curves of Au/rGO-3/GCE in Ru(bpy)32+ before and after it was electrochemically reduced. Fig. S13. Oxygen and carbon atoms ratio of (A) Au/rGO-2 and (B) Au/rGO-3 before and after the reaction with Ru(bpy)32+. Fig. S14. ECL performance of AuNPs with different diameters (A), GO and rGO (B), and Au/GO-1 and Au/GO-2 as well as Au/rGO-1 and Au/rGO-2 (C) in Ru(bpy)32+. Fig. S15. Ultraviolet-visible absorption spectra of Au/rGO with different GO reduction degrees (A). The logarithm of the anodic to cathodic ECL luminescence intensity of Ru(bpy)32+ reacting with Au/rGO with different rGO reduction times (B). Fig. S16. The effect of (A) pH, (B) C[Au/rGO-2]/C[Au/rGO-3], and (C) Ru(bpy)32+’s concentration on lg(Ic/Ia) signal output of the immunosensor. Fig. S17. The comparison of Au/rGO-2 with traditional Ru(bpy)32+’s cathodic co-reactant GSH, K2S2O8, and H2O2. Scheme S1. The schematic illustration for cathodic and anodic ECL reaction pathways. Table S1. Comparison of the different potential-resolved platforms for ratiometric ECL immunoassay. Table S2. The XPS atomic of C1s, N1s, O1s, and Au4f on Au/rGO synthesized at different concentrations of HAuCl4 [file research.0117.f1.zip › Scheme S1.pdf]
